# Software using artificial intelligence for nodule and cancer detection in CT lung cancer screening: systematic review of test accuracy studies

**DOI:** 10.1136/thorax-2024-221662

**Published:** 2024-09-25

**Authors:** Julia Geppert, Asra Asgharzadeh, Anna Brown, Chris Stinton, Emma J Helm, Surangi Jayakody, Daniel Todkill, Daniel Gallacher, Hesam Ghiasvand, Mubarak Patel, Peter Auguste, Alexander Tsertsvadze, Yen-Fu Chen, Amy Grove, Bethany Shinkins, Aileen Clarke, Sian Taylor-Phillips

**Affiliations:** 1 Warwick Screening & Warwick Evidence, Warwick Medical School, University of Warwick, Coventry, UK; 2 Population Health Science, University of Bristol, Bristol, UK; 3 Warwick Evidence, Warwick Medical School, University of Warwick, Coventry, UK; 4 Department of Radiology, University Hospitals Coventry and Warwickshire NHS Trust, Coventry, UK; 5 Research Centre for Healthcare and Communities, Coventry University, Coventry, UK; 6 Warwick Screening, Warwick Medical School, University of Warwick, Coventry, UK

**Keywords:** Imaging/CT MRI etc, Lung Cancer, Non-Small Cell Lung Cancer, Clinical Epidemiology

## Abstract

**ABSTRACT:**

**Objectives:**

To examine the accuracy and impact of artificial intelligence (AI) software assistance in lung cancer screening using CT.

**Methods:**

A systematic review of CE-marked, AI-based software for automated detection and analysis of nodules in CT lung cancer screening was conducted. Multiple databases including Medline, Embase and Cochrane CENTRAL were searched from 2012 to March 2023. Primary research reporting test accuracy or impact on reading time or clinical management was included. QUADAS-2 and QUADAS-C were used to assess risk of bias. We undertook narrative synthesis.

**Results:**

Eleven studies evaluating six different AI-based software and reporting on 19 770 patients were eligible. All were at high risk of bias with multiple applicability concerns. Compared with unaided reading, AI-assisted reading was faster and generally improved sensitivity (+5% to +20% for detecting/categorising actionable nodules; +3% to +15% for detecting/categorising malignant nodules), with lower specificity (−7% to −3% for correctly detecting/categorising people without actionable nodules; −8% to −6% for correctly detecting/categorising people without malignant nodules). AI assistance tended to increase the proportion of nodules allocated to higher risk categories. Assuming 0.5% cancer prevalence, these results would translate into additional 150–750 cancers detected per million people attending screening but lead to an additional 59 700 to 79 600 people attending screening without cancer receiving unnecessary CT surveillance.

**Conclusions:**

AI assistance in lung cancer screening may improve sensitivity but increases the number of false-positive results and unnecessary surveillance. Future research needs to increase the specificity of AI-assisted reading and minimise risk of bias and applicability concerns through improved study design.

**PROSPERO registration number:**

CRD42021298449.

**WHAT IS ALREADY KNOWN ON THIS TOPIC**

- Artificial intelligence (AI)-based software is increasingly used to assist the detection and measurement of pulmonary nodules as part of lung cancer screening, but its impact on test accuracy and clinical management has not been comprehensively critiqued and summarised.

**WHAT THIS STUDY ADDS**

- AI assistance in lung cancer screening tends to increase sensitivity (detecting more cancers) but at the cost of reduced specificity (resulting in significant additional surveillance of nodules, which would never develop into cancer).
- Evidence was mostly from retrospective studies conducted in research settings with high risk of bias and applicability concerns.

**HOW THIS STUDY MIGHT AFFECT RESEARCH, PRACTICE OR POLICY**

- Adoption of AI software and further research should focus on improving the specificity of AI assistance and prospective collection of evidence from in-practice settings using robust study design.

## Introduction

 Early detection, assessment, monitoring and timely intervention of pulmonary nodules are the key approach to reducing lung cancer morbidity and mortality. Lung cancer screening programmes have been established in several countries including the USA, Croatia, Czech Republic and Taiwan following growing evidence demonstrating survival benefits.^1 2^ In September 2022, the UK National Screening Committee recommended targeted lung cancer screening using low-dose CT for people aged 55–74 identified as being at high risk of lung cancer.^3^

Recommendations for nodule management differ across guidelines internationally,^4^ but most rely on measuring the diameter or the volume of the nodule to help determine next steps. Many individuals with nodules are placed under regular CT surveillance to assess whether the nodule is growing. Obtaining an accurate manual measurement of nodules can be challenging; nodules present in a wide range of different shapes and sizes. There is evidence of substantial inter-reader and intra-reader variability, and that variability increases the more complex the nodule morphology is.^5^ In the recently published Dutch–Belgian lung cancer screening trial (NELSON), 9.2% of the CT scans were indeterminate (ie, showed either a solid nodule with a volume of 50–500 mm³, pleural-based solid nodules with a minimal diameter of 5–10 mm or a solid nodule with a non-solid component with a mean diameter of ≥8 mm).^6^ All these individuals required a repeat CT scan in 3 months to calculate volume-doubling time. As the proportion of people with nodules detected on CT scans is high, the accurate measurement and appropriate management of nodules have significant implications for radiologist time and potential patient anxiety.

Computer-aided detection (CAD) systems for assisting radiologists in reading CT scans, which rely on predefined rules, thresholds and patterns, have been available for many years. They were used in the NELSON trial,^6^ the UKLS trial,^7^ the Multicentric Italian Lung Detection trial^8^ and the ongoing Yorkshire Lung Screening Trial.^9^ Different types of software using modern forms of artificial intelligence (AI) capable of automatically detecting and measuring pulmonary nodules have become available and could potentially reduce the screening workload and reading time for radiologists. These operate differently to traditional CAD systems; they do not rely on predefined rules and instead learn task-relevant features and generate algorithms from raw input data.

We aimed to examine the accuracy of CE-marked (compliant with relevant European Union regulations), AI-based software use for automated detection and analysis of pulmonary nodules in chest CT scans as part of lung cancer screening. As secondary outcomes, we analysed the reading time and the provided information on the impact of AI assistance on Lung CT Screening Reporting & Data System (Lung-RADS) categorisation.

## Methods

### Protocol and registration

This systematic review is an update of part of a diagnostic technology assessment for the National Institute for Health and Care Excellence.^10^ The protocol for the original systematic review was registered with PROSPEROCRD42021298449. This paper is reported according to the Preferred Reporting Items for Systematic Reviews and Meta-Analyses (PRISMA) extension for diagnostic test accuracy studies.^11^

### Data sources

We conducted literature searches on 17–19 January 2022 and updated these on 6 March 2023. The search strategy was based on three themes: lung cancer/nodules, AI and computer tomography/mass screening/early detection of cancer. Databases searched were MEDLINE, Embase, Cochrane Database of Systematic Reviews, Cochrane CENTRAL, Health Technology Assessment (HTA) database (CRD), International HTA database (INAHTA), Science Citation Index Expanded (Web of Science), Conference Proceedings—Science (Web of Science). Endnote V.20 was used to identify and remove duplicate results.

We searched or reviewed websites of selected conference proceedings, health technology assessment organisations, device manufacturers and devices@FDA between 24 January and 16 February 2022. Forward citation tracking from key publications of included studies was also undertaken in May 2022, using Science Citation Index (Web of Science) and Google Scholar. Details of the search strategies are provided in online supplemental material 1. Reference lists of included studies and recent, relevant systematic reviews identified via the database searches were checked.

### Study selection

Two reviewers independently reviewed titles and abstracts of all retrieved records and all potentially eligible full-text publications against inclusion criteria. Disagreements were resolved by consensus or discussion with a third reviewer. Studies were eligible for inclusion if they reported test accuracy of AI-based software for automated detection and analysis of lung nodules from CT images performed for lung cancer screening or secondary outcomes relating to the impact on clinical management and practical implications. We included all AI-based software which had (or was anticipated to have) an appropriate regulatory approval (CE mark) across the UK and the EU by December 2021 and was near-market—that is, with anticipated availability for commercial use by 2023. The reference standard for lung nodule presence/absence was experienced radiologist reading. Lung cancer presence was confirmed by histological analysis of lung biopsy or health record review; lung cancer absence was confirmed by CT surveillance (imaging follow-up) without significant nodule growth or follow-up without lung cancer diagnosis. Eligible outcomes included test accuracy for nodule detection and/or risk categorisation based on size (any nodules, actionable nodules and malignant nodules, respectively), impact on clinical management and practical implications. Eligible study designs were test accuracy studies, randomised controlled trials, cohort studies, historically controlled trials, before–after studies and retrospective multireader multicase (MRMC) studies. We included peer-reviewed papers; conference abstracts and manufacturer data were only included if they were related to an eligible peer-reviewed full-text paper and reported additional outcome data.

We excluded studies using PET-CT scan images, lung phantom images or where less than 90% were CT images taken for lung cancer screening. We excluded studies if traditional CAD systems without deep learning were used, or they had no relevant test accuracy or clinical management outcomes, and non-human studies along with letters, editorials and communications unless they reported outcome data not reported elsewhere, in which case they were handled in the same way as conference abstracts. We excluded articles not available in English or published before 2012.

### Data extraction and quality assessment

Detailed information related to study design, sampling of patients or CT scan images, AI-based software, reference standard and test accuracy outcomes was collected from each included study. Data allowing construction of 2×2 tables were extracted where possible, to calculate sensitivity and specificity. The unit of analyses (per person or per nodule) and features of detected/missed nodules were noted. Comparative data on the potential or actual impact of AI assistance on clinical management (eg, risk categorisation of lung nodules according to clinical guidelines based on measured nodule sizes) and time required by readers to interpret and report findings of the CT scans were also collected.

One reviewer extracted data into a predesigned electronic data collection form (online supplemental material 2). Data extraction sheets were checked by a second reviewer. Any disagreements were resolved through discussion, with the inclusion of a third reviewer when required. Study quality was assessed independently by two reviewers using QUADAS-2^12^ combined with the QUADAS-C tool for comparative studies,^13^ tailored to the review question (online supplemental material 3). Assessment of applicability was based on a UK/EU frame of reference. Disagreements were resolved through consensus, with the inclusion of a third reviewer if required.

### Data analysis

We focused on comparisons between trained human readers (radiologists or other trained healthcare professionals) assisted by AI-based software and those undertaking unassisted reading of CT scan images as this reflects current use of the technology in clinical practice. Supplementary evidence from other comparisons (ie, performance of stand-alone software vs unassisted reading) or non-comparative test accuracy studies (ie, AI-assisted reading or stand-alone software vs reference standard) were also reported where available. We calculated sensitivities and specificities in paired forest plots for the detection of any nodules, actionable nodules and malignant nodules. Where data allowed, we plotted our findings in receiver operating characteristic (ROC) space. Given the substantial heterogeneity in study populations, technologies, reader specialty and experiences, reference standards, test accuracy outcomes used and other study design features, no meta-analysis was carried out and findings are summarised narratively. Secondary outcomes such as reading time and impact on Lung-RADS ratings were summarised narratively.

## Results

### Study selection

We retrieved 6330 unique results in January 2022, of which 4886 were published since 2012. Nine records were judged to be relevant,^14^,^22^ and two records were identified from other sources.^23 24^ Update searches in March 2023 yielded an additional 1687 results, only one was identified as potentially eligible^25^ but was subsequently excluded. Eleven studies were, therefore, included (see online supplemental material 4 for full PRISMA flow diagram). Reasons for exclusions at full-text level are listed in online supplemental material 5.

### Study characteristics

Characteristics of included studies are presented in table 1.^14^,^24^ They comprised 19 770 screened participants. There is potential for overlap as some studies may have sampled the same patients while using the same databases. Two studies used data from the Korean Lung Cancer Screening Project^15 16^ and four studies used US National Lung Screening Trial (NLST) data.^18^,^2022^ Three studies were conducted in the USA.^14 18 20^ Two studies reported data from the same screening programme in South Korea.^15 16^ One study was conducted in each of the UK,^23^ Taiwan^17^ and China.^21^ Two studies conducted in the Netherlands and Denmark^22^ and in South Korea,^19^ respectively, utilised CT scan images from the US NLST. The remaining reader study was conducted in the Netherlands using ultra-low-dose CT images from Russia.^24^ Eight studies adopted an MRMC design.^17^,^24^ Two of these used unaided reading originally carried out as part of clinical practice for the comparators.^21 23^ Four studies sampled consecutive patients,^15 16 21 23^ and six used nodule-enriched samples,^17^,^2022 24^ while the remaining study adopted random sampling.^14^

**Table 1:** Characteristics of included studies

Study, country, software, time period for CT scan	Design, setting, sampling method and sample size	Index test and comparator(s) *	Reader details/reading conditions	Reference standard	Reported outcomes
Chamberlin *et al*USAAI-Rad Companion, prototype VA10A (Siemens Healthineers)January 2018–July 2019^14^	Retrospective test accuracy study (non-comparative).Random sample from 1 US centre;117 LDCT scans.	(A) Stand-alone AI	NA	**Nodules**: Consensus of two expert radiologists	**Accuracy for detecting nodules >6 mm (per person and per nodule analysis**)
Hall *et al*UKVeolity, version 1.2 (MeVis)Date unclear: LSUTNovember 2015–July 2017^23^	Retrospective test accuracy study and MRMC study (fully paired).Consecutive sample from UK-based LSUT;735 LDCT scans.	(C) Concurrent AI(E) Original unaided reader	(C) MRMC: Two radiographers without prior experience in thoracic CT reporting.(E) Clinical practice (LSUT): Five radiologists with 5–28 years of experience in thoracic imaging (5% double reading).	**Clinically significant nodules**: Original radiologist reading or consensus of two independent radiologists after reviewing discrepant readings between study radiographers and original radiologists.**Cancer**: NR	**Accuracy for detecting clinically significant lung nodules ≥5 mm;accuracy for detecting malignant nodules;**reading time
Hsu *et al*TaiwanClearRead CT, market version (Riverain Technologies)January–December 2017^17^	MRMC study (fully paired).Consecutive cases with nodules ≤10 mm or no nodules from one hospital in Taiwan;150† CT images (57 LDCT from lung cancer screening).	(B) Second-read AI(C) Concurrent AI(D) Unaided reader	MRMC: Three residents in radiology andthree experienced chest radiologists.Two reading sessions with 8-week washout period: first unaided reading followed by second-read AI, then concurrent AI; images in random order.	**Nodules**: Consensus of two thoracic radiologists with >15 years of experience	**Accuracy for detecting any nodules (stratified by seniority of readers**)
Hwang *et al*South KoreaAVIEW Lungscreen (Coreline Soft)April 2017–March 2018^15^	Before-and-after study (unpaired).Consecutive participants from K-LUCAS (11–14 institutions):1821 participants (before);4666 participants (after).	(C) Concurrent AI(E) Original unaided reader	Clinical practice:attending thoracic radiologists from 14 institutions (unpaired design)	**Cancer**: Review of medical records	**Accuracy of detecting and categorising actionable nodules to detect lung cancer (Lung-RADS category ≥3**)
Hwang *et al*South KoreaAVIEW Lungscreen (Coreline Soft)April 2017–December 2018^16^	Retrospective analysis of prospective cohort study (non-comparative).10 424 consecutive participants from K-LUCAS (14 institutions)	(C) Concurrent AI	Clinical practice:25 radiologists from 14 institutions with 5–38 years of experience;no comparator.	**Cancer**: Review of medical records; lung cancer diagnosed within 1 year (primary outcome) or any time after LDCT (secondary outcome)	**Accuracy of detecting and categorising actionable nodules to detect lung cancer (Lung-RADS category ≥3**)
Jacobs *et al*USA/Denmark/ NetherlandsVeolity, version 1.5 (MeVis)August 2002–August 2004^22^	MRMC study (fully paired).Random sample from NLST (baseline and round 1),40 cases from each Lung-RADS category;160 LDCT scans.	(C) Concurrent AI(D) Unaided reader	MRMC: Three radiologists with >5 years of experience and four radiology residents.Two reading sessions with ≥2 weeks washout period: Half with AI support and half unaided per session; images in random order.	NA	Shift in Lung-RADS categorisation;Reading time
Lancaster *et al*Russia/NetherlandsAVIEW LCS, version 1.0.34 (Coreline Soft)February 2017–2018^24^	MRMC study (fully paired).Nodule-enriched: ≥1 solid nodule, no lung cancer diagnosed within 2 years from MLCS baseline scan;283 ultra-LDCT scans.	(A) Stand-alone AI(C) Concurrent AI(D) Unaided reader	(C) MRMC: Three thoracic radiologists with >7 years of experience in lung cancer screening.(D) MRMC: Two different thoracic radiologists with >7 years of experience in lung cancer screening (using other semi-automated volume measurement software).	**Nodules**: Independent consensus of three experienced radiologists and one IT technologist	**Accuracy of nodule volume measurement and categorisation (<100 mm^3^, ≥100 mm^3^**)
Lo *et al*USAClearRead CT (first generation, pre-market) (Riverain Technologies)Date unclear: NLST screened from August 2002 to September 2007^18^	MRMC study (fully paired).Nodule-enriched: 2 normal cases for each case with nodules from NLST and 2 US hospitals;324 LDCT scans.	(A) Stand-alone AI(C) Concurrent AI(D) Unaided reader	MRMC: 12 general radiologists with 6–26 years of experience.Two reading sessions with a minimum interval of 37 days (mean, 57 days): first unaided; then AI-assisted.	**Actionable nodules**: Consensus of three expert thoracic radiologist assisted by corresponding NLST or source documentation.**Cancer**: Histological findings (presence) or long-term follow-up (absence).	**Accuracy for detecting actionable nodules;accuracy for detecting malignant nodules;**reading time
Park *et al*USA/South KoreaVUNO Med-LungCT AI, v.1.0.1 (VUNO)Date unclear: NLST baseline screen from August 2002^19^	MRMC study (fully paired).Nodule-enriched from NLST baseline screens;200 LDCT scans.	(A) Stand-alone AI(C) Concurrent AI(D) Unaided reader	MRMC: One radiology resident and four radiologists with 1–20 years of experience.Two reading sessions with 6-week washout period: first unaided, then AI-assisted; images in random order.	**Cancer**: NR(Lung cancer diagnosed within 1 year in the NLST)	**Accuracy of detecting and categorising actionable nodules to detect lung cancer (Lung-RADS category ≥3**)Change in Lung-RADS category
Singh *et al*USAClearRead CT, market version (Riverain Technologies)Date unclear: NLST screened from August 2002 to September 2007^20^	MRMC study (fully paired).Nodule-enriched: 100 with SSNs and 23 without SSNs from NLST;123 LDCT scans.	(A) Stand-alone AI(C) Concurrent AI(vessel suppression only)(D) Unaided reader	MRMC: Two radiologists with 5 and 10 years of thoracic CT experience; sequential interpretation of unprocessed CT images alone, then vessel-suppressed images without washout period.	**Sub-solid nodules**: Consensus of two experienced thoracic radiologists (11 and 27 years of experience) with adjudication of conflicts by a third radiologist	**Accuracy for detecting sub-solid nodules ≥6 mm;**Change in Lung-RADS category
Zhang *et al*ChinaInferRead CT Lung, market version (Infervision)November to December 2019^21^	Retrospective test accuracy study and MRMC study (fully paired).Consecutive sample from one hospital in China;860 LDCT scans.	(C) Concurrent AI (MRMC study)(E) Original unaided reader (clinical practice)	(C) MRMC: One resident with 5 years of experience with supervision by one radiologist with 20 years of experience.(E) Clinical practice: One resident drafted report with supervision by radiologist (14 residents and 15 radiologists).	**Nodules**: Consensus of two radiologists with 20 and 31 years of experience	**Accuracy for detecting any nodules (stratified by types and sizes of nodules**)

* Index test and comparators: ([A)] Stand-alone AI: analysis of CT scan image by AI-based software without human input; ([B)] Second-read AI: CT scan image was firstlyfirst reviewed by an unaided human reader, then was re-interpreted after analysis by AI-based software was shown; ([C)] Concurrent AI: CT scan image was reviewed by a human reader assisted by concurrent display of analysis by AI-based software; ([D)] Unaided reader: CT scan image was reviewed by a human reader without assisted by AI-based software; ([E)] Original unaided reader: CT scan image was interpreted by a human reader as part of clinical practice, and therefore the reader was different from the human reader who interpret the CT scan image in the reader study.

† The study included mixed populations. Only those who underwent CT scans for screening were included in this systematic review.

AI, artificial intelligence; K-LUCAS, Korean Lung Cancer Screening; LDCT, low-dose CT; LSUT, Lung Screen Uptake Trial; Lung-RADS, Lung CT Screening Reporting & Data System; MLCS, Moscow Lung Cancer Screening; MRMC, multi-reader, multi-case study; NA, not applicable; NLST, National Lung Screening Trial

Six different AI-based software programs were used in the studies: *AI-Rad Companion* (Siemens Healthineers),^14^ *AVIEW Lungscreen* (Coreline Soft),^15 16 24^ *ClearRead* (Riverain Technologies),^17 18 20^ *InferRead CT Lung* (Infervision),^21^ *VUNO Med LungCT AI* (VUNO)^19^ and *Veolity* (MeVis).^22 23^

### Risk of bias and applicability

The evidence is of low quality. There were problems in most studies in almost all domains in terms of risk of bias and applicability, given the design and operationalisation of the studies and our UK/EU frame of reference (table 2 and online supplemental material 6). Risk of bias according to QUADAS-C was considered ‘high’ in three or more domains in five of the eight comparative studies.^17 18 20 23 24^ These issues included no consecutive or random sampling, test set laboratory studies in which radiologist behaviour is known to differ from clinical practice,^26^ unpaired design (before/after study or different radiologists with and without AI) and/or suboptimal or biased reference standard.

**Table 2:** Limitations of the included studies

Reference and country	Applicability concerns regardingEuropean screening population	Design concerns	Laboratory effect^26^	Applicability concerns to European screening LDCT reading practice	Applicability concerns—actionable nodule definition	Bias in reference standard	Nodule detection or measurement only	Accuracy reported per nodule only
Chamberlin *et al,*USA^14^	**Yes—**1 US centre; 55–80 years.Excluded images rejected by software due to slice thickness >3 mm or poor image quality.	**Yes—**Non-comparative (A)	(A) NA	**Yes—**Stand-alone AI	**Yes—**>6 mm	**Nodules: Yes—**<3 experienced chest radiologist; not blinded to index test	**Yes—**Detection of actionable nodules	**No**
Hall *et al*,UK^23^	**No**	**Yes—**Fully paired CT images but not the same readers with and without AI.	(C) **Yes—**MRMC	**Yes—**(C) Two inexperienced radiographers.(E) 5% double reading.	**No**	**Nodules: Yes—**Original radiologist report (E) plus radiologist review of scans with additional nodules detected by (C).	**Yes—**Detection of actionable nodules.Detection of malignant nodules ((C) only).	**No**
			(E) **No**			**Cancer: Unclear—**Cancers detected either from baseline scan or nodule surveillance.		
Hsu *et al,*Taiwan^17^	**Yes—**One centre in Taiwan; 31/57 never smoked.Selected cases with nodule≤1 cm or no nodules.Exclusion of cases with severe pulmonary fibrosis, diffuse bronchiectasis, extensive inflammatory consolidation, pneumothorax, and massive pleural effusion. 2.5 mm slice thickness.	**No**	(A) NA	**Yes—**(A) Stand-alone AI.(B) (C) (D) Three residents in radiology; three experienced chest radiologists.	**Yes—**No size cut-off used.	**Nodules: Yes—**<3 experienced chest radiologists; not blinded to index test.	**Yes—**Detection of any nodules.	**Yes—**Per-nodule sensitivity; per-person specificity.
			(B) (C) (D) **Yes—**MRMC					
Hwang *et al,*South Korea^15^	**Yes—**11 (before) and 14 (after) institutions in Korea (K-LUCAS).Only 145/6,487 (2.2%) women.	**Yes—**(A) Non-comparative.(C) (E) Unpaired non-randomised; not the same readers with and without AI.	(A) NA	(A) **Yes—**Stand-alone AI.	(A) **Yes—**No size cut-off used.	**Nodules: Yes—**Single radiologist with second-read AI.**Cancer: Yes—**Medical record review.	(A) **Yes—**Nodule detection (any, actionable, malignant).	(A) **Yes—**Per-nodule sensitivity.
			(C) (E) **No**	(C) (E) **No**	(C) (E) **No**		(C) (E) **No**	(C) (E) **No**
Hwang *et al,*South Korea^33^	**Yes—**14 institutions in Korea (K-LUCAS).Only 283/10,424 (2.7%) women.	**Yes—**Non-comparative (C)	**No**	**No**	**No**	**Cancer: Yes—**Medical record review.	**No**	**No**
Jacobs *et al,*NL, USA, Denmark^22^	**Yes –**US NLST (baseline and round 1).Nodule-enriched; Lung-RADS ≥3 120/160 (75%).Slice thickness 1.0 to 3.2 mm.	**No**	(C) (D)**Yes—**MRMC	**Yes—**Three radiologists with >5 year of experience and four radiology residents (fifth year).	**No**	**NA**	**NA**	**NA**
Lancaster *et al,*NL, Russia^24^	**Yes—**Ultra-LDCT (≤1 mSv); 50–80 years.Selected participants who had ≥1 solid nodule and did not develop lung cancer in following 2 years.	**Yes—**Not the same readers with and without AI.	(A) NA	(A) **Yes—**Stand-alone AI	**No**	**Nodule size and categorisation: Yes—**2/4 consensus panel readers involved in index test.	**Yes**—Nodule measurement and risk category.	**No**
			(C) (D) **Yes—**MRMC	(C) (D) **No**				
Lo *et al,*USA^18^	**Yes—**US NLST and 2 US hospitals;nodule-enriched (1:2);3/178 nodules ≥3 mm.	**No**	(A) NA	**Yes—**(A) Stand-alone AI.(C) (D) 12 general radiologists.	**No**	**Nodules: NoCancer: No**	**Yes—**Detection of nodules (actionable, malignant).	**Yes—**Per-nodule sensitivity; per-person specificity.
			(C) (D) **Yes—**MRMC					
Park *et al,*South Korea, USA^19^	**Yes –**US NLST;Nodule and cancer-enriched:Prevalence of Lung-RADS≥3 127/200 (64%);lung cancer prevalence 31/200 (16%).	**No**	(A) NA	**Yes—**(A) Stand-alone AI.(C) (D) 1 of 5 readers was a fourth-year radiology resident.	**No**	**Cancer: Unclear—**Same-year positive cancer diagnosis (not stated how diagnosed).	**No**	**No**
			(C) (D) **Yes—**MRMC					
Singh *et al,*USA^20^	**Yes—**US NLST;enriched for sub-solid nodules:prevalence of sub-solid nodules100/123 (81%).	**No**	(A) NA	(A) **Yes—**Stand-alone AI.	**No**	**Nodules: No**	**Yes—**Detection of actionable nodules.	**Yes—**Per-nodule sensitivity;Per-person specificity.
			(C) (D) **Yes—**MRMC	(C) **Unclear—**AI for vessel suppression.				
				(D) **No**				
Zhang *et al*China^21^	**Yes—**One hospital in China (part of NELCIN-B3);general population aged 45–74 years.	**Yes—**Not the same readers with and without AI.	(C) **Yes—**MRMC	**Yes—**(C) One resident supervised by 1 radiologist;(E) 1 of 14 residents supervised by 1 of 15 radiologists.	**Yes—**No size cut-off.	**Nodules: Yes—**<3 experienced chest radiologists; not blinded to index test.	**Yes—**Detection of any nodules.	**No**
			(E) **No**					
Legend	**No** =Random or consecutive screening LDCT images from heavy current or former smokers aged 50–75 years^34^ living in Europe; no inappropriate exclusions; ≤2 mm slice thickness.	**No** =Comparative, fully paired design; same readers with and without AI.	**No** =CT images assessed in clinical practice.	**No** =Single reading by experienced chest radiologist with (C) or without AI support (D) or (E).	**No** =In agreement with BTS^35^, Lung-RADS^36^ or EUPS^37^ guidelines.	**No** =Nodules: ≥3 blinded, experienced chest radiologists.Cancer: Histopathology after biopsy/excision or 2-year follow-up without cancer diagnosis.	**No** =Accuracy of detection + risk categorisation + recall for lung cancer diagnosis.	**No** =Per-person sensitivity and specificity.

Index test and comparators: ([A)] Stand-alone AI: Analysis of CT scan image by AI-based software without human input; ([B)] Second-read AI: CT scan image firstlyfirst reviewed by an unaided human reader, then re-interpreted after analysis by AI-based software was shown; ([C)] Concurrent AI: CT scan image reviewed by a human reader assisted by concurrent display of analysis by AI-based software; ([D)] Unaided reader: CT scan image reviewed by a human reader without AI-based software assistance; ([E)] Original unaided reader: CT scan image interpreted by a human reader as part of clinical practice; different to the human reader who interpreted the CT scan image in the reader study.

AIartificial intelligenceBTSBritish Thoracic SocietyCategcategorisationEUPSEuropean Position StatementK-LUCASKorean Lung Cancer Screening ProjectLDCTlow-dose CTLSUTLung Screen Uptake TrialLung-RADSLung CT Screening Reporting & Data SystemMRMCmulti-reader, multi-case studyNAnot applicableNELCIN-B3Netherlands-China Big-3 disease screeningNLNetherlandsNLSTNational Lung Screening Trial

### Test accuracy

#### AI-assisted reading versus unaided reading

Eight studies reported on AI-assisted reading, where AI-based software was used concurrently (seven studies^1518^,^21 23 24^) or in addition sequentially (also referred to as ‘second-read AI’)^17^ to re-interpret images.

**Figure 1:**
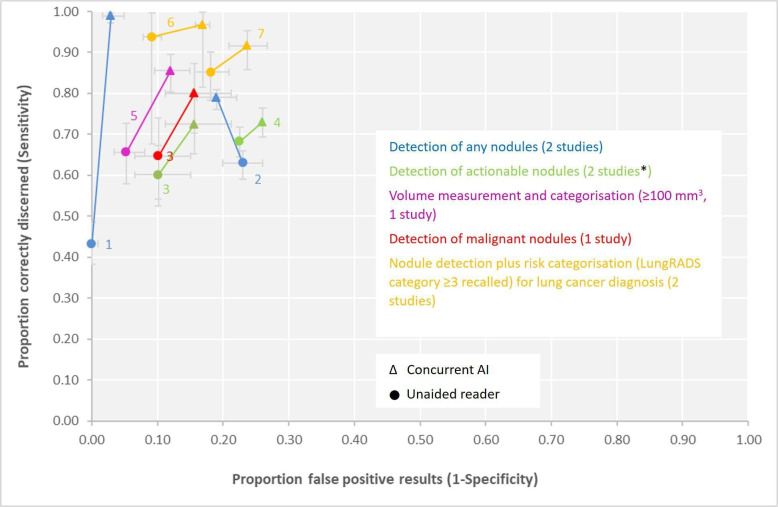
Accuracy of readers (nodule detection; nodule categorisation based on volume measurement; or nodule detection plus risk categorisation and recall decision for lung cancer diagnosis) both with and without concurrent AI use (seven studies with comparative data). Estimates connected with a line are from the same study. ^1^ Zhang *et al*^21^; ^2^ Hsu *et al*^17^; ^3^ Lo *et al*^18^; ^4^ Singh *et al*^20^; ^5^ Lancaster *et al*^24^; ^6^ Hwang *et al*^15^; ^7^ Park *et al.*^19^ *Data from Hall *et al*^23^ are not presented as the study compared AI-assisted reading by radiographers against unaided radiologists, which differed in nature from the other studies. AI, artificial intelligence; Lung-RADS, Lung CT Screening Reporting & Data System.

One study (described later) compared AI assisted radiographers (without prior experience in thoracic CT reporting) with unaided, experienced radiologists.^23^ Across all remaining seven studies, the addition of concurrent AI to trained radiologists increased sensitivity and decreased specificity compared with unaided, trained radiologists. Two studies reported detection of actionable nodules (range: +5% to +13% for sensitivity; −3% to −6% for specificity)^18 20^ and one for detecting malignant nodules (+15% for sensitivity, −6% for specificity).^18^ Two studies reported detection of lung cancer through Lung-RADS category ≥3 (range, +3% to +7% for sensitivity; −8% to −6% for specificity),^15 19^ see figure 1 and online supplemental material 7. Concurrent AI-assistance also increased sensitivity (+20%) and decreased specificity (−7%) in nodule measurement and categorisation using a volume cut-off of 100 mm^3^.^24^ For detection of nodules of any size, including nodules too small to be considered clinically actionable, radiologists’ sensitivity was increased with concurrent AI use (range, +16% to +56%), with an unclear impact on specificity (range, −3% to +4%).^17 21^ One of these studies^17^ evaluated both concurrent AI and second-read AI and found very similar sensitivity (79% vs 80%) and specificity (81% vs 82%), see online supplemental material 7 and 8.

For illustrative purposes (ie, the examples given here are plausible but hypothetical, given that test accuracy often changes as the screened population and disease prevalence varies, and the data were based on individual studies that used different AI software), if the changes in sensitivity and specificity for the detection of malignant nodules with concurrent AI assistance was in the range of those observed in the large screening programme reported by Hwang *et al*^15^ or in the MRMC study by Lo *et al*,^18^ and if the prevalence of lung cancer among the screening population was similar to that observed in the NELSON trial (ie, 0.5%),^6^ AI assistance would allow an additional 150–750 people attending screening with cancers to be detected but an additional 59 700 to 79 600 people attending screening without cancer would be placed on CT surveillance and/or further investigations per million people screened (equivalent to a reduction in positive predictive value of screening from 5% to 3%^15^ or from 3% to 2%, respectively^18^; online supplemental material 9).

### Impact on Lung-RADS categorisation

Three MRMC studies provided comparative data on the impact of AI assistance on Lung-RADS categorisation of nodules.^19 20 22^ The proportion of actionable nodules identified (Lung-RADS categories 3–4) was higher when images were assessed with AI assistance in all three studies (66% vs 53%,^22^ 34.2% vs 28.5%,^19^ 55% vs 50%^20^). However, no reference standards were used, so it is not possible to know whether the additional actionable nodules were malignant.

### Impact on CT scan reading time

Three comparative MRMC studies reported on the impact of AI assistance on reading times.^18 22 23^ Reading times were significantly faster with AI assistance compared with unaided readers: median 86 (IQR 51–141) seconds vs 160 (IQR 96–245) seconds (p<0.001)^22^ and mean 98.0 seconds vs 132.3 seconds per case (p<0.01)^18^ for radiologists, and median 3 (IQR 2–5) and 5 (IQR 4–8) min for radiographers using AI in a laboratory (ie, non-clinical) setting vs 10 (IQR 5–15) min for radiologists (unassisted reading in clinical practice).^23^

### Other methods of using AI (stand-alone AI and supporting less experienced staff)

Studies have also investigated other ways of using AI (comparing stand-alone AI with no human input to unaided radiologists or used AI to support less trained staff) or used non-comparative evidence (eg, AI-assisted reading or unaided reading compared with a reference standard). These are presented in online supplemental material 8.

Across studies and outcomes, stand-alone AI was associated with the highest sensitivity (range 58%–100%) but lowest specificity (62%–82%) when compared with AI-assisted radiologist reading (sensitivity 71%–99%, specificity 74%–97%) and/or unaided radiologist reading (sensitivity 43%–94%, specificity 63%–97%) (online supplemental material 8).^18^,^2024^

One study investigated whether AI assistance would support radiographers to match the accuracy of radiologists.^23^ Experienced radiologists were more sensitive (91% vs 71%) and specific (97% vs 92%) for detecting and categorising actionable nodules than AI-assisted reading by radiographers (without prior experience in thoracic CT reporting) (online supplemental material 8). Further decisions of experienced, unaided radiologists (made during clinical practice) were consistent with British Thoracic Society guidance 71.6% of the time, while the decisions of two radiographers with AI assistance in a laboratory setting were consistent with the guidance 39.7% and 60.7% of the time, respectively.

## Discussion

### Summary of clinical context

Targeted lung cancer screening programmes are being set up in many countries due to strong randomised controlled trial (RCT) evidence that screening leads to a reduction in lung cancer-specific mortality. This will, however, place enormous pressure on already over-stretched healthcare systems, particularly in terms of scanner capacity and radiologist time. Different types of software using AI-derived algorithms have become available and could potentially reduce the screening workload and reading time for radiologists. These AI-based software, however, also have the potential to cause patient harm or create further workload for radiologists, and evidence is required to determine their performance in a screening context. Here, we have reported the results of a systematic review, synthesising the available evidence on the accuracy, reading time and impact on clinical management.

### Statement of principal findings

Our searches yielded 6573 publications, from which 11 heterogeneous studies, reporting on nearly 20 000 patients from six different countries and using six different AI-based software systems were included. All 11 studies were at high risk of bias with multiple applicability concerns. We used a narrative approach to summarise our results, finding that AI-assisted reading was faster and generally improved sensitivity (range: +5% to +20% for detecting/categorising actionable nodules; +3% to +15% for detecting/categorising malignant nodules), with lower specificity (range: −7% to −3% for correctly detecting/categorising people without actionable nodules; −8% to −6% for correctly detecting/categorising people without malignant nodules) compared with unaided reading. AI assistance tended to increase the proportion of nodules allocated to higher risk categories. If these findings were replicated in a population of a million people attending screening, the impact of AI would be an extra 150–750 cancers detected at the cost of 59 700–79 600 people receiving unnecessary surveillance, reducing positive predictive value.

### Strengths and limitations

Our searches were extensive but limited by date (January 2012–March 2023). The 2012 cut-off was introduced after discussion with experts who considered that our definition of AI would not include systems introduced or tested prior to that date. Our searches are also limited to studies published in the English language although this is unlikely to have biased our findings.^27 28^ We aimed to include all AI-based software, which had (or was anticipated to have) appropriate regulatory marking (CE mark) across the UK and the EU, with anticipated availability for commercial use by 2023. However, our searches were inclusive, and we were unlikely to have omitted significant studies from our research because of this inclusion criterion.

QUADAS-2 was used independently by two reviewers^12^ combined with the QUADAS-C tool for comparative studies,^13^ which we tailored to the review question to assess risk of bias and applicability. Almost all the studies fell short in key elements of quality, including patient selection, definition of reference standard, index test and flow and timing. The studies we identified were extremely heterogeneous using six different AI-based software systems and from at least six different countries, where the epidemiology of lung cancer, training of radiologists and experience of use of CT screening for lung cancer differ substantially. Therefore, we undertook a narrative review and plotted our findings in ROC space, however if it was possible, meta-analysis would allow for more precise estimates of the accuracy of the addition of AI-based software to CT lung cancer screening. We acknowledge that the potential benefit of AI assistance (150–750 additional lung cancers detected in a screened population of a million people) will depend on the prevalence of lung cancer in the cohort and as such is not generalisable to other populations at higher or lower risk. In addition, software derived from AI potentially allows continuous improvement of performance through learning from expanding sources of data. Although the various softwares evaluated in our review did not involve learning from data in real time, companies may refine their software by retraining their AI models with new datasets and then update the AI-derived algorithms used in the software periodically. Published evaluations on the performance of AI-based software in screening are, therefore, only a snapshot and could be outdated by the time when they are published, and our findings might not completely reflect systems that are currently available. The AI software that we evaluated only processed and utilised data from CT scan images to enhance nodule segmentation, detection and measurement that underpin current practice based on contemporary guidelines. Use of AI software to combine and interrogate additional morphological data from scan images (radiomics) along with a wide range of demographic, histological, proteomic and genomic data for prediction of nodules that are malignant is an area of very active research. These advances could fundamentally change clinical practice in the future. Nevertheless, it is crucial that any claims of improvement in risk stratification and cancer detection with AI software are supported by robust evidence generated from studies with strong designs that address risk of bias and applicability concerns that we highlighted.

### Strengths and weaknesses versus other studies

We identified 12 previous systematic reviews on the accuracy of AI for lung nodule/cancer detection and/or malignancy risk prediction in medical images. Nine of these were non-comparative and focused on stand-alone AI performance of algorithms that were not commercially available, so were not informative for our review question (references are reported in online supplemental material 10). One rapid review^29^ was comparative but focused on the accuracy of AI-based software for the classification of lung nodules into benign or malignant, a software function that was not included in our review.

Two reviews^30 31^ did cover our question but were broader and did not separately report on the screening population or on commercially available software. Li *et al*^31^ evaluated the impact of AI on physicians’ performance in detecting various thoracic pathologies on CT and chest X-ray. The review by Ewals *et al*.^30^ was more relevant but covered not only the screening population but also the oncologic, symptomatic or mixed populations as well as software that was not commercially available. Of our 11 included papers, only one^20^ was identified in the review by Li *et al*^31^ and three^17 18 21^ in the review by Ewals *et al*.^30^ Despite the broader population in the review by Ewals *et al*, they found a similar pattern of increased sensitivity and reduced specificity with AI use. However, Li *et al* found that, across all pathologies and both image types, both sensitivity and specificity generally improved when using AI-based devices. In concordance with our review, a faster reading time was reported with concurrent AI use in both previous reviews.^30 31^

## Conclusions and implications for clinicians and policymakers

Our systematic review demonstrates that, when used in population-based lung cancer screening programmes, assistance of AI-based software can increase sensitivity, but at the expense of a reduction in specificity, that is, an increase in false-positive findings. The lung checks in the NHS England Targeted Lung Health Checks programme are already supported by AI,^32^ and removing AI-based software from existing screening programmes is not a practical policy option. However, the limited available evidence suggests that there is significant scope for improvement in the AI-based software, particularly in specificity. This is particularly important to consider as the screening programme is rolled out in the UK, given the potential increase in false-positive findings and the resulting additional workload for radiologists and anxiety for patients. Furthermore, care must be taken that AI-based software does not contribute to changing disease definitions or referral thresholds as the limited evidence base suggests its measurements and categorisations are more cautious and biased towards greater referral. Finally, more research is needed particularly in clinical settings and around the impact of AI assistance on medical staff with less training. Prospective, comparative, test accuracy studies that measure accuracy of the whole testing pathway with AI assistance integrated in clinical practice and compare it with the accuracy of the pathway without AI assistance are needed.

## supplementary material

10.1136/thorax-2024-221662 online supplemental file 1

## Data Availability

All data relevant to the study are included in the article or uploaded as supplementary information.
