# Supplementary material for: Software using artificial intelligence for nodule and cancer detection in CT lung cancer screening: systematic review of test accuracy studies
Source: Thorax. 2024 Sep 25;79(11):e221662. doi: 10.1136/thorax-2024-221662 (PMC11503082; doi:10.1136/thorax-2024-221662)
Supplement: online supplemental file 1 [file thorax-79-11-s001.pdf]

Software using artificial intelligence for nodule and cancer detection in CT lung cancer screening: systematic review of test accuracy studies

Supplementary materials

Julia Geppert [1 2] ^, Asra Asgharzadeh [1 3] ^, Anna Brown [1], Chris Stinton[1 2], Emma Helm [4], Surangi Jayakody [1], Dan Todkill [1], Daniel Gallacher [1], Hesam Ghiasvand [1 5], Mubarak Patel [1], Peter Auguste [1], Alexander Tsertsvadze [1], Yen-Fu Chen [1 *], Amy Grove [1], Bethany Shinkins [1 2], Aileen Clarke [1], Sian Taylor-Phillips [2]

[1] Warwick Evidence, Division of Health Sciences, Warwick Medical School, University of Warwick, Coventry, United Kingdom

[2] Warwick Screening, Division of Health Sciences, Warwick Medical School, University of Warwick, Coventry, United Kingdom

[3] Population Health Science, Bristol Medical School, University of Bristol, Bristol, United Kingdom

[4] University Hospitals Coventry and Warwickshire, Coventry, United Kingdom

[5] Research Centre for Healthcare and Communities, Coventry University, Coventry, United Kingdom

^Joint first author

*Corresponding author: Dr Yen-Fu Chen, e-mail: Y-F.Chen@warwick.ac.uk

Table of Contents

Supplementary material 1: Search strategies	3
Supplementary material 2: Data extraction form	20
Supplementary material 3: QUADAS-2 and QUADAS-C tailored to the review question, with guidance notes	21
Supplementary material 4: PRISMA diagram. Summary of publications included and excluded at each stage of the review (original searches / update searches)	36
Supplementary material 5: Publications and sub-studies excluded after review of full-text articles	37
Supplementary material 6: Quality assessment results based on QUADAS-2 and QUADAS-C tools (10 studies)	58
Supplementary material 7: Comparative evidence on sensitivity and specificity for AI-assisted reading compared with unaided reading by radiologists for the detection and/or categorisation of lung nodules in CT images for lung cancer screening	62
Supplementary material 8: Evidence on AI-assisted reading compared with unaided reading and stand-alone AI for accuracy of detecting and/or categorising any nodules, actionable nodules and malignant nodules	63
Supplementary material 9: Illustrative calculations for the potential impact of introducing AI assistance in a lung cancer screening programme	65
Supplementary material 10: References for the 9 identified non-comparative systematic reviews on stand-alone AI software performance of algorithms that are not commercially available	68

Supplementary material 1: Search strategies

Overview (Original search):

Bibliographic databases and trials registers		
**Database / register**	**Date searched**	**Number of records**
MEDLINE All	17/01/22	2,740
Embase	17/01/22	3,495
Cochrane Library (CENTRAL and Cochrane Database of Systematic reviews)	17/01/22	131 (all from CENTRAL; 0 results from CDSR)
Science Citation Index and Conference Proceedings – Science (Web of Science)	19/01/22	3,210
HTA database (CRD)	19/01/22	1
INAHTA HTA database	19/01/22	3
medRxiv	19/01/22	7
clinicaltrials.gov	19/01/22	17
WHO ICTRP	19/01/22	22
Total number of records retrieved: 9,626<br>Duplicates removed (EndNote): 3,296<br>**Final number for screening: 6,330**		
Other sources		
**Source**	**Date searched**	**Documents retrieved**
National Institute for Health and Care Excellence (NICE) website	24/01/22	3
Canadian Agency for Drugs and Technologies in Health (CADTH) website	24/01/22	7
ISPOR conference presentations	25/01/22	0
HTAi annual meetings	25/01/22	1
SPIE proceedings	27/01/22	14
IEEE Engineering in Medicine & Biology Society annual conference	27/01/22	1
European Congress of Radiology	31/01/22	52
Radiological Society of North America annual meetings	01/02/22	55
FDA devices databases	14/02/22	5
device / manufacturer websites	15-16/02/22	15 documents, plus 1 link to video presentation
reference lists – selected systematic reviews	Checked manually by reviewers	
reference lists – included studies		
forwards citation tracking:<br>Science Citation Index (Web of Science) & Google Scholar	26/05/22 &<br>30/05/22	44
**Total: 197**		
Search update: bibliographic databases and trials registers		
**Database / register**	**Date searched**	**Number of records**
MEDLINE All	06/03/23	3,335
Embase	06/03/23	4,916
Cochrane Library (CENTRAL and Cochrane Database of Systematic reviews)	06/03/23	193 (192 from CENTRAL; 1 from CDSR)
Science Citation Index and Conference Proceedings – Science (Web of Science)	06/03/23	4,063

HTA database (CRD)	n/a (database no longer updated)	
INAHTA HTA database	06/03/23	3
clinicaltrials.gov	06/03/23	21
WHO ICTRP	06/03/23	30

Total number of records retrieved: 12,561
Duplicates removed (both within this set and against original search results from January 2022): 10,874
Final number for screening: 1,687

Search strategies:

MEDLINE ALL
Date searched: 17/01/22
Ovid MEDLINE(R) ALL <1946 to January 14, 2022>

1 exp artificial intelligence/ or exp machine learning/ or exp deep learning/ or exp supervised machine learning/ or exp support vector machine/ or exp unsupervised machine learning/ 134273
2 ai.kf,tw. 34062
3 ((artificial or machine or deep) adj5 (intelligence or learning or reasoning)).kf,tw. 89902
4 exp Neural Networks, Computer/ 42235
5 (neural network* or convolutional or CNN or CNNs).kf,tw. 73835
6 exp Diagnosis, Computer-Assisted/ 85513
7 ((computer aided or computer assisted) adj1 (diagnosis or detection)).kf,tw. 6018
8 (support vector machine* or random forest* or black box learning).kf,tw. 31141
9 1 or 2 or 3 or 4 or 5 or 6 or 7 or 8 322906
10 exp Lung Neoplasms/di, dg or Solitary Pulmonary Nodule/di, dg 56493
11 ((lung or lungs or pulmon* or bronchial) adj3 (nodul* or cancer* or neoplas* or tumor* or tumour* or carcino* or malignan* or adenocarcinom* or blastoma*)).kf,tw. 274199
12 ((pulmonary or lung) adj2 lesion*).kf,tw. 14782
13 10 or 11 or 12 302352
14 Tomography, X-Ray Computed/ or exp Tomography, Spiral Computed/ 418962
15 (comput* adj2 tomograph*).kf,tw. 348023
16 (CT or LDCT).kf,tw. 388825
17 (CAT adj2 (scan* or x-ray* or xray*)).kf,tw. 1342
18 Mass Screening/ 111594
19 ((lung or lungs or pulmon*) adj3 (nodule* or cancer* or tumor* or tumour*) adj3 screen*).kf,tw. 4813
20 "Early Detection of Cancer"/ 31774
21 14 or 15 or 16 or 17 or 18 or 19 or 20 893125
22 9 and 13 and 21 2767
23 (aview* lcs* or clearread* ct* or inferread* ct lung* or lung nodule ai* or veolity* or veye).kf,tw. 7
24 ((ai rad companion* and chest) or contextflow* or search lung ct* or "jld 01k*" or qct lung* or sensecare* lung* or visia* ct* or vuno).kf,tw. 8
25 (coreline* or riverain* or infervision* or fujifilm* or mevis* or aidence*).in,kf,tw. 1381
26 (siemens* healthineers* or contextflow* or jlk inc* or arterys* or qureai* or qure ai* or sensetime* or canon medical* or vuno*).in,kf,tw. 1407
27 (25 or 26) and (10 or 11) 159
28 22 or 23 or 24 or 27 2867
29 exp animals/ not humans/ 4943529
30 28 not 29 2851
31 limit 30 to english language 2740

Search update: an identical search was run on 06/03/23 and retrieved 3335 results.

The artificial intelligence search terms (lines 1-4 & 6) are based on those used in:

Freeman K, Geppert J, Stinton C, Todkill D, Johnson S, Clarke A et al. Use of artificial intelligence for image analysis in breast cancer screening programmes: systematic review of test accuracy BMJ 2021; 374 :n1872 doi:10.1136/bmj.n1872 (see online supplementary appendix 1)

Selected lung cancer/nodule search terms (lines 11-12) were informed by those used in: Duarte A, Corbett M, Melton H, Harden M, Palmer S, Soares M, Simmonds M. EarlyCDT Lung for
lung cancer risk classification of solid pulmonary nodules: A Diagnostics Assessment Report. York
EAG, 2021. Available from: https://www.nice.org.uk/guidance/indevelopment/gid-dg10041/documents (accessed 9 November 2021)

Embase
Date searched: 17/01/22
Embase <1974 to 2022 January 14>

1 exp artificial intelligence/ or exp machine learning/ 304838
2 ai.kf,tw. 45921
3 ((artificial or machine or deep) adj5 (intelligence or learning or reasoning)).kf,tw. 105922
4 (neural network* or convolutional or CNN or CNNs).kf,tw. 89201
5 computer assisted diagnosis/ 40877
6 ((computer aided or computer assisted) adj1 (diagnosis or detection)).kf,tw. 8264
7 (support vector machine* or random forest* or black box learning).kf,tw. 38837
8 1 or 2 or 3 or 4 or 5 or 6 or 7 420312
9 exp lung cancer/di or lung nodule/di 46922
10 ((lung or lungs or pulmon* or bronchial) adj3 (nodul* or cancer* or neoplas* or tumor* or tumour* or carcino* or malignan* or adenocarcinom* or blastoma*)).kf,tw. 392765
11 ((pulmonary or lung) adj2 lesion*).kf,tw. 21058
12 9 or 10 or 11 420629
13 computer assisted tomography/ or low-dose computed tomography/ or exp x-ray computed tomography/ or multidetector computed tomography/ or spiral computer assisted tomography/ or computed tomography scanner/ 931594
14 (comput* adj2 tomograph*).kf,tw. 445065
15 (CT or LDCT).kf,tw. 664348
16 (CAT adj2 (scan* or x-ray* or xray*)).kf,tw. 2036
17 mass screening/ or cancer screening/ 142872
18 screening/ 184110
19 ((lung or lungs or pulmon*) adj3 (nodule* or cancer* or tumor* or tumour*) adj3 screen*).kf,tw. 7644
20 early cancer diagnosis/ 9899
21 13 or 14 or 15 or 16 or 17 or 18 or 19 or 20 1643282
22 8 and 12 and 21 3370
23 (aview* lcs* or clearread* ct* or inferread* ct lung* or lung nodule ai or veolity* or veye).dv,kf,tw. 11
24 (qct lung* or vuno*).dv. 0
25 ((ai rad companion* and chest) or contextflow* or search lung ct* or "jld 01k*" or sensecare* lung* or visia* ct*).dv,kf,tw. 4

26 (coreline* or riverain* or infervision* or fujifilm* or mevis* or aidence*).dm,in,kf,tw. 5146
27 (siemens* healthineers* or contextflow* or jlk inc* or arterys* or qureai* or qure ai* or sensetime* or canon medical* or vuno*).dm,in,kf,tw.4797
28 (26 or 27) and (9 or 10) 436
29 22 or 23 or 24 or 25 or 28 3692
30 (exp animal/ or exp animal experiment/) not (exp human/ or exp human experiment/ or conference abstract.pt.) 4770834
31 29 not 30 3673
32 limit 31 to english language 3495

Search update: an identical search was run on 06/03/23 and retrieved 4916 results.

Cochrane Library (via www.cochranelibrary.com)
Date searched: 17/01/22
Cochrane Central Register of Controlled Trials, Issue 12 of 12, December 2021
Cochrane Database of Systematic Reviews, Issue 1 of 12, January 2022

ID Search Hits
#1 [mh "artificial intelligence"] OR [mh "machine learning"] OR [mh "deep learning"] OR [mh "supervised machine learning"] OR [mh "support vector machine"] OR [mh "unsupervised machine learning"] 1261
#2 ai:ti,ab,kw 4506
#3 ((artificial OR machine OR deep) NEAR/5 (intelligence OR learning OR reasoning)):ti,ab,kw 2857
#4 [mh "Neural Networks, Computer"] 148
#5 ((neural NEXT network*) OR convolutional OR CNN OR CNNs):ti,ab,kw 1479
#6 [mh "Diagnosis, Computer-Assisted"] 1931
#7 (("computer aided" OR "computer assisted") NEAR/1 (diagnosis OR detection)):ti,ab,kw 1001
#8 (("support vector" NEXT machine*) OR (random NEXT forest*) OR "black box learning"):ti,ab,kw 776
#9 #1 OR #2 OR #3 OR #4 OR #5 OR #6 OR #7 OR #8 10964
#10 [mh "Lung Neoplasms"/DI,DG] OR [mh ^"Solitary Pulmonary Nodule"/DI,DG] 653
#11 ((lung OR lungs OR pulmon* OR bronchial) NEAR/3 (nodul* OR cancer* OR neoplas* OR tumor* OR tumour* OR carcino* OR malignan* OR adenocarcinom* OR blastoma*)):ti,ab,kw 25143
#12 ((pulmonary OR lung) NEAR/2 lesion*):ti,ab,kw 533
#13 #10 OR #11 OR #12 25426
#14 [mh ^"Tomography, X-Ray Computed"] OR [mh "Tomography, Spiral Computed"] 4555
#15 (comput* NEAR/2 tomograph*):ti,ab,kw 20680
#16 (CT OR LDCT):ti,ab,kw 81013
#17 (CAT NEAR/2 (scan* OR x-ray* OR xray*)):ti,ab,kw 34
#18 [mh ^"Mass Screening"] 3339
#19 ((lung OR lungs OR pulmon*) NEAR/3 (nodule* OR cancer* OR tumor* OR tumour*) NEAR/3 screen*):ti,ab,kw 758
#20 [mh ^"Early Detection of Cancer"] 1384
#21 #14 OR #15 OR #16 OR #17 OR #18 OR #19 OR #20 96454
#22 #9 AND #13 AND #21125

#23 ((aview* NEXT lcs*) OR (clearread* NEXT ct*) OR (inferread* NEXT "ct" NEXT lung*) OR ("lung nodule" NEXT ai*) OR veolity* OR veye) 2
#24 (("ai rad" NEXT companion*) AND chest) OR contextflow* OR ("search lung" NEXT ct*) OR (jld NEXT 01k*) OR (qct NEXT lung*) OR (sensecare* NEXT lung*) OR (visia* NEXT ct*) OR vuno* 2
#25 coreline* OR riverain* OR infervision* OR fujifilm* OR mevis* OR aidence* 152
#26 (siemens* NEXT healthineers*) OR contextflow* OR (jlk NEXT inc*) OR arterys* OR qureai* OR (qure NEXT ai*) OR sensetime* OR (canon NEXT medical*) OR vuno* 57
#27 (#25 OR #26) AND (#10 OR #11) 6
#28 #22 OR #23 OR #24 OR #27 in Cochrane Reviews, Trials 131

Search update: an identical search was run on 06/03/23 and retrieved 193 results.

The Ovid Medline search strategy was translated for use in the Cochrane Library and Web of Science with the aid of the Polyglot Search Translator:
Clark JM, Sanders S, Carter M, Honeyman D, Cleo G, Auld Y, et al. Improving the translation of search strategies using the Polyglot Search Translator: a randomized controlled trial. J Med Libr Assoc 2020;108(2):195-207.
http://dx.doi.org/10.5195/jmla.2020.834

Science Citation Index and Conference Proceedings - Science (via Web of Science)
Date searched: 19/01/2022
SCI-EXPANDED: 1970-present
CPCI-S: 1990-present

23 (((#17) OR #18) OR #19) OR #22 and English (Languages) 3,210
22 (#20 OR #21) AND #7 AND #16 216
21 ((((TS=("siemens* healthineers*" OR contextflow* OR "jlk inc*" OR arterys* OR qureai* OR "qure ai*" OR sensetime* OR "canon medical*" OR vuno*)) OR OG=("siemens* healthineers*" OR contextflow* OR "jlk inc*" OR arterys* OR qureai* OR "qure ai*" OR sensetime* OR "canon medical*" OR vuno*)) OR AD=("siemens* healthineers*" OR contextflow* OR "jlk inc*" OR arterys* OR qureai* OR "qure ai*" OR sensetime* OR "canon medical*" OR vuno*)) OR FO=("siemens* healthineers*" OR contextflow* OR "jlk inc*" OR arterys* OR qureai* OR "qure ai*" OR sensetime* OR "canon medical*" OR vuno*)) 2,633
20 ((((TS=(coreline* OR riverain* OR infervision* OR fujifilm* OR mevis* OR aidence*)) OR OG=(coreline* OR riverain* OR infervision* OR fujifilm* OR mevis* OR aidence*)) OR AD=(coreline* OR riverain* OR infervision* OR fujifilm* OR mevis* OR aidence*)) OR FO=(coreline* OR riverain* OR infervision* OR fujifilm* OR mevis* OR aidence*)) 3,964
19 TS=(("ai rad companion*" AND chest) OR contextflow* OR "search lung ct*" OR "jld 01k*" OR "qct lung*" OR "sensecare* lung*" OR "visia* ct*" OR vuno) 8
18 TS=("aview* lcs*" OR "clearread* ct*" OR "inferread* ct lung*" OR "lung nodule ai*" OR veolity* OR veye) 5
17 ((#6) AND #9) AND #16 3,085
16 #10 or #11 or #12 or #13 or #14 or #15 655,436
15 TS=("Early Detection of Cancer") 2,106
14 TS=((lung OR lungs OR pulmon*) NEAR/3 (nodule* OR cancer* OR tumor* OR tumour*) NEAR/3 screen*) 6,299
13 TS=("Mass Screening") 5,559
12 TS=(CAT NEAR/2 (scan* OR x-ray* OR xray*)) 1,067

11	TS=(CT OR LDCT)	455,518
10	TS=(comput* NEAR/2 tomograph*)	361,422
9	#7 OR #8	380,001
8	TS=((pulmonary OR lung) NEAR/2 lesion*)	14,221
7	TS=((lung OR lungs OR pulmon* OR bronchial) NEAR/3 (nodul* OR cancer* OR neoplas* OR tumor* OR tumour* OR carcino* OR malignan* OR adenocarcinom* OR blastoma*))	370,649
6	#1 OR #2 OR #3 OR #4 OR #5	901,467
5	TS=("support vector machine*" OR "random forest*" OR "black box learning")	133,456
4	TS=(("computer aided" OR "computer assisted") NEAR/2 (diagnosis OR detection))	16,891
3	TS=("neural network*" OR convolutional OR CNN OR CNNs)	501,511
2	TS=((artificial OR machine OR deep) NEAR/5 (intelligence OR learning OR reasoning))	395,814
1	TS=(ai)	75,151

Search update: an identical search was run on 06/03/23 and retrieved 4063 results.

The Ovid Medline search strategy was translated for use in the Cochrane Library and Web of Science with the aid of the Polyglot Search Translator:
Clark JM, Sanders S, Carter M, Honeyman D, Cleo G, Auld Y, et al. Improving the translation of search strategies using the Polyglot Search Translator: a randomized controlled trial. J Med Libr Assoc 2020;108(2):195-207.
http://dx.doi.org/10.5195/jmla.2020.834

HTA Database (via CRD https://www.crd.york.ac.uk/CRDWeb/)
Date searched: 19/01/22

1	MeSH DESCRIPTOR Artificial Intelligence EXPLODE ALL TREES	290
2	(ai)	202
3	((artificial OR machine OR deep) ADJ5 (intelligence OR learning OR reasoning))	8
4	(neural network* OR convolutional OR CNN OR CNNs)	12
5	MeSH DESCRIPTOR Diagnosis, Computer-Assisted EXPLODE ALL TREES	108

6	((computer aided OR computer assisted) ADJ1 (diagnosis OR detection))	34
7	(support vector machine* OR random forest* OR black box learning)	0
8	(#1 OR #2 OR #3 OR #4 OR #5 OR #6 OR #7) IN HTA	148
9	((lung* or pulmon*) ADJ3 (nodul* or cancer* or neoplas* or tumor* or tumour* or carcino* or malignan* or adenocarcinom*))	1444
10	MeSH DESCRIPTOR Lung Neoplasms EXPLODE ALL TREES	1151
11	MeSH DESCRIPTOR Solitary Pulmonary Nodule EXPLODE ALL TREES	27
12	(#9 OR #10 OR #11) IN HTA	341
13	MeSH DESCRIPTOR Tomography, X-Ray Computed	896
14	MeSH DESCRIPTOR Tomography, Spiral Computed EXPLODE ALL TREES	75
15	(comput* ADJ2 tomograph*)	1395
16	(CT OR LDCT)	1231
17	(CAT ADJ2 (scan* OR x-ray* OR xray*))	6
18	MeSH DESCRIPTOR Mass Screening	2103

19	((lung OR lungs OR pulmon*) ADJ3 (nodule* OR cancer* OR tumor* OR tumour*) ADJ3 screen*)	42
20	MeSH DESCRIPTOR Early Detection of Cancer EXPLODE ALL TREES	277
21	(#13 OR #14 OR #15 OR #16 OR #17 OR #18 OR #19 OR #20) IN HTA	953
22	#8 AND #12 AND #21	1

International HTA database (via INAHTA https://database.inahta.org/)
Date searched: 19/01/22

21	#20 AND #14 AND #8	3
20	#19 OR #16 OR #15	417
19	#18 AND #17	383
18	nodul* OR cancer* OR neoplas* OR tumor* OR tumour* OR carcino* OR malignan* OR adenocarcinom*	3216
17	lung* OR pulmon*	866
16	"Lung Neoplasms"[mhe]	318
15	"Solitary Pulmonary Nodule"[mh]	6
14	#13 OR #12 OR #11 OR #10 OR #9	2443
13	tomograph* OR radiograph* OR CT OR x-ray* OR xray* OR MRI OR PET	813
12	screening	1234
11	"Diagnostic Imaging"[mhe]	1127
10	"Mass Screening"[mhe]	758
9	"Early Detection of Cancer"[mh]	71
8	#7 OR #6 OR #5 OR #4 OR #3 OR #2 OR #1	189
7	"Artificial Intelligence"[mhe]	85
6	"Diagnosis, Computer-Assisted"[mhe]	64
5	"Neural Networks, Computer"[mhe]	0
4	"artificial intelligence" OR "machine learning" OR "deep learning" OR "deep reasoning" OR "machine reasoning"	9
3	"neural network" OR "neural networks" OR convolutional OR CNN OR CNNs	5
2	"computer aided" OR "computer assisted"	65
1	"support vector machine*" OR "random forest*" OR "black box learning"	0

Search update: an identical search was run on 06/03/23 and retrieved the same 3 results.

medRxiv (via medrxivr https://mcguinlu.shinyapps.io/medrxivr/)
Date searched: 19/1/22

Advanced search screen:

Topic 1:
[Aa]rtificial [Ii]ntelligence
[Mm]achine [Ll]earning
[Dd]eep [Ll]earning
[Ss]upport [Vv]ector [Mm]achine
\\b[Aa][Ii]\\b
[Nn]eural [Nn]etwork
[Cc]onvolutional
[Rr]andom [Ff]orest
[Bb]lack [Bb]ox [Ll]earning
[Cc]omputer [Aa]ided [Dd]iagnosis

[Cc]omputer [Aa]ssisted [Dd]iagnosis
[Cc]omputer [Aa]ided [Dd]etection
[Cc]omputer [Aa]ssisted [Dd]etection
\\bCNN\\b
\\bCNNs\\b
[Dd]eep [Rr]easoning
[Mm]achine [Rr]easoning

Topic 2:
[Ll]ung
[Pp]ulmon

Topic 3:
0[Nn]eoplas
[Cc]ancer
[Nn]odul
[Tt]umor
[Tt]umour
[Cc]arcinoma
[Aa]denocarcinoma

Topic 4:
[Cc]omputed [Tt]omograph
\\bCT\\b
\\bLDCT\\b
screening

Earliest record date:
2016-07-01

Latest record date:
2022-01-19

Remove older versions of the same record

clinicaltrials.gov
Date searched: 19/01/22

Home screen search: https://clinicaltrials.gov/ct2/home

3 Studies found for: "aview lcs" OR "aview lcs+" OR "clearread ct" OR "inferread ct lung" OR "inferread lung" OR "lung nodule ai" OR veolity OR veye [Other terms]

10 Studies found for: coreline* OR riverain OR infervision OR fujifilm OR aidoc OR mevis OR aidence ['Other terms']| lung OR pulmonary [Condition or disease] *(of which 3 studies already found above)*

2 Studies found for: "ai rad companion" OR contextflow OR "search lung ct" OR "jld 01k" OR "lung ai" OR "qct lung" OR sensecare OR vuno [Other terms]

5 Studies found for: "siemens healthineers" OR jlk OR qureai OR "qure ai" OR sensetime [Other terms]| lung OR pulmonary [Condition or disease]

Total: 17 unique results

Search update: an identical search was run on 06/03/23 and retrieved 21 results.

WHO International Clinical Trials Registry Platform (ICTRP) search portal
Date searched: 19/01/22

Home screen search: https://trialsearch.who.int/Default.aspx

7 records for 7 trials found for: aview lcs* OR clearread ct OR inferread ct lung OR inferread lung OR lung nodule ai OR veolity OR veye

9 records for 9 trials found for: (coreline* OR riverain OR infervision OR fujifilm OR aidoc OR mevis OR aidence) AND (lung OR pulmonary)

9 records for 8 trials found for: ai rad companion OR contextflow OR search lung ct OR jld 01k OR qct lung OR sensecare OR vuno

No results were found for: (siemens healthineers OR jlk OR qureai OR qure ai OR sensetime OR arterys) AND (lung OR pulmonary)

Advanced search screen: https://trialsearch.who.int/AdvSearch.aspx

1 records for 1 trials found for: lung ai [in the intervention]
without synonyms selected; recruitment status is ALL

Total number of trials after 3 duplicates removed (using EndNote): **22**

Search update: an identical search was run on 06/03/23 and retrieved 30 results.

NICE website https://www.nice.org.uk/

Date searched: 24/01/22

Browsed: NICE Guidance > Conditions and diseases > Cancer > Lung cancer:
https://www.nice.org.uk/guidance/conditions-and-diseases/cancer/lung-cancer
found 76 published products, of which **3** downloaded/of potential interest

Searched published guidance: https://www.nice.org.uk/guidance/published?sp=on
Filters (Guidance programme): Technology appraisal guidance, NICE guidelines, Clinical guidelines, Medical technologies guidance, Diagnostics guidance, Highly specialised technologies guidance, Cancer service guidelines.

lung cancer 51 results, of which 1 potentially relevant, already identified above
nodule 3 results, of which 1 potentially relevant, already identified above

Searched published guidance: https://www.nice.org.uk/guidance/published?sp=on

No filters.

artificial intelligence 3 results, of which 1 potentially relevant, already identified above
machine learning 0 results
deep learning 0 results
ai 1 result, of which 0 relevant
neural network 0 results

Browsed guidance In consultation: https://www.nice.org.uk/guidance/inconsultation
12 results, 0 relevant to lung cancer/pulmonary nodules or artificial intelligence

Total unique results downloaded: 3

Canadian Agency for Drugs and Technologies in Health (CADTH) website
https://www.cadth.ca/

Date searched: 24/01/22

Search screen: https://www.cadth.ca/search , results limited to Reports tab.

Search terms:

lung cancer [contains all words] 74 results; 8 potentially relevant, of which 1 already identified via bibliographic database searches

nodules nodule [contains any words] 9 results; 5 potentially relevant, all 5 already identified above

artificial intelligence [contains all words] 31 results; 3 potentially relevant, all 3 already identified above

machine learning [contains all words] 17 results; 2 potentially relevant, both already identified above

deep learning [contains all words] 11 results; 2 potentially relevant, both already identified above

ai 20 results; 2 potentially relevant, both already identified above

neural networks [contains all words] 5 results; 1 potentially relevant, already identified above

Total unique results downloaded: 7

ISPOR presentations database **https://www.ispor.org/heor-resources/presentations-database/search**
Date searched: 25/01/22
As there was no option to export results in bulk, titles and, where necessary abstracts, were scanned for potential relevance and only those potentially relevant to AI technologies *and* CT imaging *and* lung cancer/pulmonary nodules were retrieved (where not already identified by previous searches).

search	hits	documents retrieved
lung cancer AND (tomograph* OR CT OR LDCT OR screening)	70	0 (1 potentially relevant already identified via database searches)
pulmonary nodule* AND (tomograph* OR CT OR LDCT OR screening)	3	0
lung nodule* AND (tomograph* OR CT OR LDCT OR screening)	4	0
lung AND ("artificial intelligence" OR "machine learning" OR "deep learning" OR ai OR "neural networks" OR "neural network")	15	0
pulmonary AND ("artificial intelligence" OR "machine learning" OR "deep learning" OR ai OR "neural networks" OR "neural network")	7	0
**Total documents retrieved:**		**0**

Health Technology Assessment International (HTAi) Annual Meetings
https://htai.org/annual-meetings/

Date searched: 25/01/22

HTAi 2021 Virtual (Manchester). Full program available at:
https://htai.org/wp-content/uploads/2021/06/HTAi_AM21_Full-Program.pdf
Searched (Ctrl + F) for:
lung
pulmon
chest
thora
artificial int
learning
neural *nothing relevant found*

HTAi 2020 Beijing (virtual). Poster abstracts and Oral abstracts available from:
https://htai.eventsair.com/htaibeijing2020
Scanned titles in poster and abstract e-books (no search function available); 1 potentially relevant (oral abstract)

HTAi 2019 Cologne. Abstract book available at:
https://htai.org/wp-content/uploads/2019/08/htai_AM19_abstracts_20190812.pdf
Searched (Ctrl + F) for:
lung
pulmon
chest

thora
artificial int
learning
neural *nothing relevant found*

Total documents retrieved: 1

SPIE Proceedings (via SPIE Digital Library https://www.spiedigitallibrary.org/)

Date searched: 26/01/22
Advanced search screen; search in: Proceedings

("lung cancer" OR "pulmonary nodule") AND ("artificial intelligence" OR "machine learning" OR "deep learning" OR "neural network") AND (screening OR tomography OR CT OR LDCT)
Refine by: Year 2012-2022

285 results; of which 14 potentially relevant *and* not already identified via the bibliographic database searches

Annual International Conference of the IEEE Engineering in Medicine & Biology Society (via IEEE Xplore)

Date searched: 27/01/22
Command search screen: https://ieeexplore.ieee.org/search/advanced/command

"Parent Publication Number":1000269 AND ((lung OR pulmonary) NEAR/3 (nodule OR cancer OR neoplas* OR tumor OR tumour OR carcinoma OR malignan* OR adenocarcinoma)) AND (ai OR ((artificial OR machine OR deep) NEAR/5 (intelligence OR learning OR reasoning)) OR "neural network" OR "neural networks" OR convolutional OR CNN OR CNNs OR (("computer aided" OR "computer assisted") NEAR/1 (diagnosis OR detection)) OR "support vector machine*" OR "random forest*" OR "black box learning") AND (tomograph* OR CT OR LDCT OR screening)

14 results; of which 13 already identified via the bibliographic database searches
1 paper downloaded

European Congress of Radiology (via European Society of Radiology website https://www.myesr.org/congress/about-ecr/past-congresses)

Date searched: 31/1/22

ECR 2021. Abstract book available at:
https://insightsimaging.springeropen.com/track/pdf/10.1186/s13244-021-01014-5.pdf
ECR 2020. Abstract book available at:
https://insightsimaging.springeropen.com/track/pdf/10.1186/s13244-020-00851-0.pdf
ECR 2019. Abstract book available at:
https://insightsimaging.springeropen.com/track/pdf/10.1186/s13244-019-0713-y.pdf

ECR 2018. Abstract book available at: https://link.springer.com/article/10.1007/s13244-018-0603-8
ECR 2017. Abstract book available at:
https://insightsimaging.springeropen.com/track/pdf/10.1007/s13244-017-0546-5.pdf
ECR 2016. Abstract book B - Scientific Sessions and Clinical Trials in Radiology, available at: https://link.springer.com/content/pdf/10.1007/s13244-016-0475-8.pdf
ECR 2015. Abstract book B - Scientific Sessions and Late-Breaking Clinical Trials, available at: https://link.springer.com/content/pdf/10.1007/s13244-015-0387-z.pdf
ECR 2014. Abstract book B - Scientific Sessions, available at:
https://link.springer.com/content/pdf/10.1007/s13244-014-0317-5.pdf

Searched (Ctrl + F) for:
lung ca
lung nod
pulmonary nod
artificial int
machine learning
deep learning
neural net

Number of abstracts downloaded (potentially relevant to AI + CT/screening + lung cancer/nodules; obvious phantom studies, prediction models and PET-CT excluded):
2021: 5
2020: 17
2019: 19
2018: 4
2017: 2
2016: 1
2015: 3
2014: 1
Total: 52 (0 already identified via other searches)

Radiological Society of North America annual meetings (via RSNA website: https://www.rsna.org/annual-meeting/future-and-past-meetings)

Date searched: 01/02/22

RSNA 2020 meeting program available at: https://www.rsna.org/-/media/Files/RSNA/Annual-meeting/Program/RSNA-2020-program.ashx
posters: *unable to access posters without an RSNA members' login*

RSNA 2019
scientific sessions available at: https://archive.rsna.org/2019/ScienceSessions.pdf
posters: a *list of titles is available, but no abstracts/further details accessible without an RSNA members' login*

RSNA 2018:
scientific sessions available at: https://archive.rsna.org/2018/ScienceSessions.pdf
posters and exhibits available at: https://archive.rsna.org/2018/PostersandExhibits.pdf

RSNA 2016 meeting program available at:
scientific sessions available at: https://archive.rsna.org/2016/ScienceSessions.pdf
posters and exhibits available at: https://archive.rsna.org/2016/PostersandExhibits.pdf

Searched (Ctrl + F) within documents for:
lung ca
lung nod
pulmonary nod
artificial int
machine learning
neural net
deep learning *[except in 2019 & 2018 Scientific Sessions, where there were too many (200+) results to scan]*

RSNA 2017:
No PDF documents available.
Meeting program available at: http://rsna2017.rsna.org/program/index.cfm
Searched for:

lung cancer
pulmonary nodule
pulmonary nodules
lung nodule
lung nodules
artificial intelligence
machine learning

Number of abstracts downloaded (potentially relevant to AI + CT/screening + lung cancer/nodules; obvious phantom studies, prediction models and PET-CT excluded):
2020: 2
2019: 17
2018: 17
2017: 14
2016: 5
Total: 55

U.S. Food & Drug Administration (FDA) Premarket Notification, Premarket Approval & De novo databases (via FDA website)

Date searched: 14/02/22
Search interfaces:

- Premarket Approval (PMA) database, 'Device' field https://www.accessdata.fda.gov/scripts/cdrh/cfdocs/cfPMA/pma.cfm
- 510(k) Premarket Notification database, 'Device Name' field https://www.accessdata.fda.gov/scripts/cdrh/cfdocs/cfPMN/pmn.cfm
- Device Classification Under Section 513(f)(2)(De Novo) database, ‘device name’ field https://www.accessdata.fda.gov/scripts/cdrh/cfdocs/cfPMN/denovo.cfm

Search terms	PMA database results	510(k) database results	De novo database results	**Documents downloaded** (judged to contain potentially useful/relevant information not already identified in previous sets)
ai rad companion	0	7	0	1
aview lcs	0	1	0	1
clearread	1	2	0	1
contextflow	0	0	0	
search lung	0	0	0	
inferread	0	2	0	1
jld-01k	0	0	0	
lung AI	0	3	0	
lung nodule	0	4	0	
qct lung	0	1	0	
search lung	0	0	0	
sensecare	0	0	0	
veolity	0	1	0	1
veye	0	0	0	
vuno	0	0	0	
**Total:**				**5**

Websites relating to the technologies of interest/their manufacturers
Dates searched: 15-16/02/22

AI-Rad Companion Chest CT / Siemens Healthineers
https://www.siemens-healthineers.com/ searched for 'AI-Rad Companion'.
Downloaded 1 'White paper' and checked its references (all potentially relevant references already identified via database searches).

AVIEW LCS+ / Coreline Soft. Browsed:
https://www.corelinesoft.com/aview-lcs-2/aview-lcs-plus/
https://www.corelinesoft.com/aview-lcs-2/
https://www.corelinesoft.com/newsroom-eng/
0 documents to download

ClearRead CT / Riverain Technologies
https://www.riveraintech.com/clearread-ai-solutions/clearread-ct/ 1 reference on page, already identified via database searches

https://www.riveraintech.com/resources/clinical-evidence/#clearread-ct-studies links to 5 papers, of which 1 not already found via database searches; **1 downloaded (Van Leeuwen 2021)**

SEARCH Lung CT / contextflow
https://contextflow.com/solution/search-for-3d-medical-imaging/ 0 to download
https://contextflow.com/startup-news/ **1 press release mentions not-yet-published study and 1 video presentation about the same study.**

InferRead CT Lung / Infervision. Browsed:
https://global.infervision.com/product/19/
https://global.infervision.com/news/5/
https://global.infervision.com/news/6/
0 documents to download

JLD-01K / JLK Inc
https://www.jlkgroup.com/en/medihub.html 0 documents to download

Lung AI / Arterys
https://www.arterys.com/clinicalapp/lungapp - references 'Arterys Lung AI Nodule Detection study - University of California, San Diego' – unable to find this via Google search
https://www.arterys.com/clinical-evidence - nothing on Lung AI; 0 documents to download

Lung Nodule AI / Fujifilm. Browsed:
https://www.fujifilm.com/uk/en/healthcare/healthcare-it
https://synapse.fujifilm.eu/ai-lab/#(grid|filter)=.radiology;
0 documents to download

qCT-Lung / Qure.ai. Browsed:
https://qure.ai/product/qct-lung/
https://qure.ai/evidences/
0 documents to download

SenseCare-Lung Pro / Sensetime. Browsed:
https://www.sensetime.com/en/product-detail?categoryId=32629
https://www.sensetime.com/en/news-index
0 documents to download

MeVis / Veolity. Browsed
https://www.veolity.com/
https://www.veolity.com/news-events
0 documents to download

Aidence / Veye Lung Nodules
https://www.aidence.com/veye-lung-nodules/
https://www.aidence.com/development-clinical-validation/ **2 conference posters and 1 unpublished manuscript downloaded**
https://www.aidence.com/clinical-research/ 5 articles/reports, of which 1 CQC report not identified via previous searches; **1 document downloaded**
https://www.aidence.com/resources/
https://www.aidence.com/articles/ **6 articles downloaded** (including 3 from an external site, 2 of which are in Dutch)

VUNO Med-LungCT AI / VUNO
https://www.vuno.co/en/lung
https://www.vuno.co/en/publication/lists/medical_image 10 articles/abstracts of potential interest, of which 2 RSNA abstracts not already identified via other searches; **2 downloaded**

Supplementary material 2: Data extraction form

EVIDENCE ID	STUDY NAME (Author Year)	EXTRACTOR	CHECKER										
**PATIENT SAMPLING ITEMS**	PATIENT SAMPLING	**PATIENT CHARACTERISTICS AND SETTING ITEMS**	PATIENT CHARACTERISTICS AND SETTING	**INDEX TEST ITEMS**	INDEX TEST (software-based nodule detection and analysis)	**COMPARATOR ITEMS**	COMPARATOR (no software for nodule detection or analysis)	**REFERENCE STANDARD ITEMS**	REFERENCE STANDARD	**FLOW AND TIMING ITEMS**	FLOW AND TIMING	**NOTES items**	NOTES
A1 Review question relevance<br>- Q1: Test accuracy and other intermediate outcomes<br>- Q2: Clinical effectiveness<br>- Q3: Cost effectiveness		*B1 Setting*		*C1 Index test mode, e.g.<br>[A] Stand-alone AI<br>[B] 2nd read CAD<br>[C] Concurrent CAD*		*D1 Reader details (number, general or thoracic radiologist or other, experience) (continue labelling with [D], [E]... as appropriate)*		*E1 Reference standard - General approach*		*F1 What was the time interval between index and reference tests?*		*G1: Funding*	
A2 Relevant outcomes for DAR		*B2 Location (include name of institution if available)*		*C2 AI name and version/date (label different AI-based index tests with [A], [B], [C]...)*		*D2 Reading conditions (reader study, clinical practice, other details)*		*E2 Reference standard for nodule detection*		*F2 Did all patients receive the same reference standard*		*G2: Publication status*	
A3 Study design<br>(and description of groups labelled [1] [2] ...)		*B3 Dates*		*C3 Manufacturer and country*		*D3 Method of nodule detection*		*E3 Reference standard for malignant nodules*		*F3 Was the reference standard chosen based on only one of the index/comparator tests?*		*G3: Source (pre-print or Journal name)*	
A4 Aim of the study		*B4 Indication for CT scan<br>- Symptomatic<br>- Incidental (with reason)<br>- Screening<br>- CT surveillance*		*C4 Commercially available / CE mark*		*D4 Method of nodule composition/type*		*E4 Reference standard for benign nodules*		*F4 Missing data*		*G4: Author COI (including any manufacturer affiliations)*	
A5 Study type<br>1) Stand-alone software compared to nothing<br>2) Stand-alone software compared to human<br>3) Software-assisted reader compared to unassisted reader<br>4) Software-assisted reader compared to nothing<br>5) Software use in pathway		*B5 Patient characteristics<br>- Age<br>- Gender<br>- Ethnicity<br>- Smoking*		*C5 AI algorithm details*		*D4 Method of nodule size measurement (segmentation, volume, diameter)*		*E5 Reference standard for nodule composition/type*		*F5 Uninterpretable results*		*G5 Comment*	
A6 Comparative study design:<br>1) Fully Paired<br>2) Randomized<br>3) Partially paired with random subset<br>4) Partially paired with nonrandom subset<br>5) Unpaired nonrandomized<br>6) Other (please describe)		*B6 Nodule characteristics<br>- Number of nodules<br>- Nodule size<br>- Nodule type<br>- Nodule shape*		*C6 AI training and tuning details*		*D5 Method of nodule growth rate*		*E6 Reference standard for nodule segmentation and size*		*F6 Indeterminate results*			
A7 Method of participant / CT image selection<br>- Source<br>- Consecutive, random, selected (e.g. enriched), unclear		*B7 CT image acquisition<br>- CT scanner<br>- Full or partial chest<br>- With or without contrast<br>- Acquisition parameters (e.g. dose)<br>- Image reconstruction<br>- Slice thickness*		*C7 Software functionality:<br>- Nodule detection<br>- Nodule composition<br>- Nodule segmentation/ measurement<br>- Growth rate*		*D6 Blinded to reference standard*		*E7 Reference standard for nodule growth rate*		*F7 Statistical analysis*			
A8 Were cases recruited prospectively or retrospectively?		*B8 Comments*		*C8 AI software settings (e.g. threshold)*		*D7 Blinded to the results of any other index tests/comparator tests*		*E8 Was it blind to index test/comparator test*		*F8 Comment*			
A9 Sample size				*C9 Reader details (number, general or thoracic radiologist or other, experience, location)*		*D8 Threshold pre-specified*		*E9 Did it incorporate index test/comparator test*					
A10 Inclusion criteria				*C10 Reading conditions where human readers are part of the test (reader study, clinical practice, other details)*		*D9 Other information available to unassisted reader (e.g. prior CT scans, family history)*		E10 Comments					
A11 Exclusion criteria				*C11 Method for nodule detection*		*D10 Description of a whole read (up to clinical decision on discharge, CT surveillance or further diagnostic investigation)*							
A12 Study flow<br>- Screened for eligibility<br>- Eligible<br>- Not eligible (with reasons)<br>- Included in study/test set<br>- Excluded from study/test set (with reasons)<br>- Included in analysis<br>- Excluded from analysis (with reasons)				*C12 Method for nodule composition/type*		*D11 Comments*							
A13 Comment				*C13 Method for segmentation and nodule size measurement (volume, diameter)*									
				C14 Method for nodule growth determination									
				C15 Other information made available to AI system or AI assisted reader (e.g. prior CT scans, family history)									
				C16 Blinded to reference standard									
				C17 Blinded to the results of any index/comparator tests									
				C18 Threshold predefined									
				C19 Description of a whole read (up to clinical decision on discharge, CT surveillance or further diagnostic investigation)									
				C20 Comment									

Supplementary material 3: QUADAS-2 and QUADAS-C tailored to the review question, with guidance notes

First author surname and year of publication:

Name of first reviewer: *Name of second reviewer:*

Phase 1: State the review question:

Question 1) What is the accuracy of CT image analysis assisted by software for automated detection and analysis of lung nodules in people undergoing CT scans?

Patients (setting, intended use of index test, presentation, prior testing):
People who have no confirmed lung nodules or lung cancer and who are not having staging investigations or follow-up imaging for primary cancer elsewhere in the body, who have a CT scan that includes the chest:<br>• *for reasons unrelated to suspicion of lung cancer (incidental population);*<br>• *because of signs or symptoms suggestive of lung cancer (symptomatic population);*<br>• *as part of lung cancer screening (screening population);*<br><br>*People having CT surveillance for a previously identified lung nodule (surveillance population).*
Index test(s) (including human comparators):
• *CT scan review by*<br>○ *Index test [A]: any of the specified software <u>alone</u>;*<br>○ *Index test [B]: a radiologist or another healthcare professional using any of the specified software as <u>2nd reader</u>;*<br>○ *Index test [C]: a radiologist or another healthcare professional with <u>concurrent use</u> of any of the specified software;*<br>○ *Index test [D]: a radiologist or another healthcare professional <u>without</u> software assistance.*
Reference standard and target condition:
• *Target condition: Lung cancer (or lung nodules)*<br>• *Reference standard for nodule detection and nodule type: Experienced chest radiologist reading (single reader or consensus/majority reading of more than one reader).*<br>• *Reference standard for nodule size measurement and nodule growth assessment: Experienced radiologist reading (single reader or consensus/mean size or mean growth rate) or measurement of nodules after excision.*<br>• *Reference standard for malignant/benign nodules:*<br>*Malignant: Histological analysis of lung biopsy or health record review;*

Benign: CT surveillance (imaging follow-up) without significant growth, follow-up without diagnosis of lung cancer.		
**Comparative review question** (only fill this part for comparative diagnostic accuracy studies with at least 2 index tests, add more rows for index tests if needed)		
Patients:		
Index test [A] (stand-alone software)		
Index test [B] (second-read CAD)		
Index test [C] (concurrent CAD)		
Index test [D] (human reader without software)		
Reference standard and target condition:		
**Comparative study design**		
Which of the following study designs does the primary study most strongly resemble?<br>*#1 Fully Paired*<br>*#2 Randomized*<br>*#3 Partially paired with random subset*<br>*#4 Partially paired with nonrandom subset*<br>*#5 Unpaired nonrandomized*<br>*Other (please describe the study design):*		*#1 If participants receiving index test [A] and index test [B] are identical (all participants receive all index test).*<br>*#2 If each participant is randomized to receive either one index test or the other.*<br>*#3 If participants are randomly selected either to receive one index test or to undergo both index tests.*<br>*#4 If a nonrandom mechanism is used to decide whether participants receive one or both index tests.*<br>*#5 If participants receive only one of the index tests without randomization.*<br>*Other (please describe study design)*

Phase 2: Draw a flow diagram for the primary study *(adapt template below or copy from paper)*

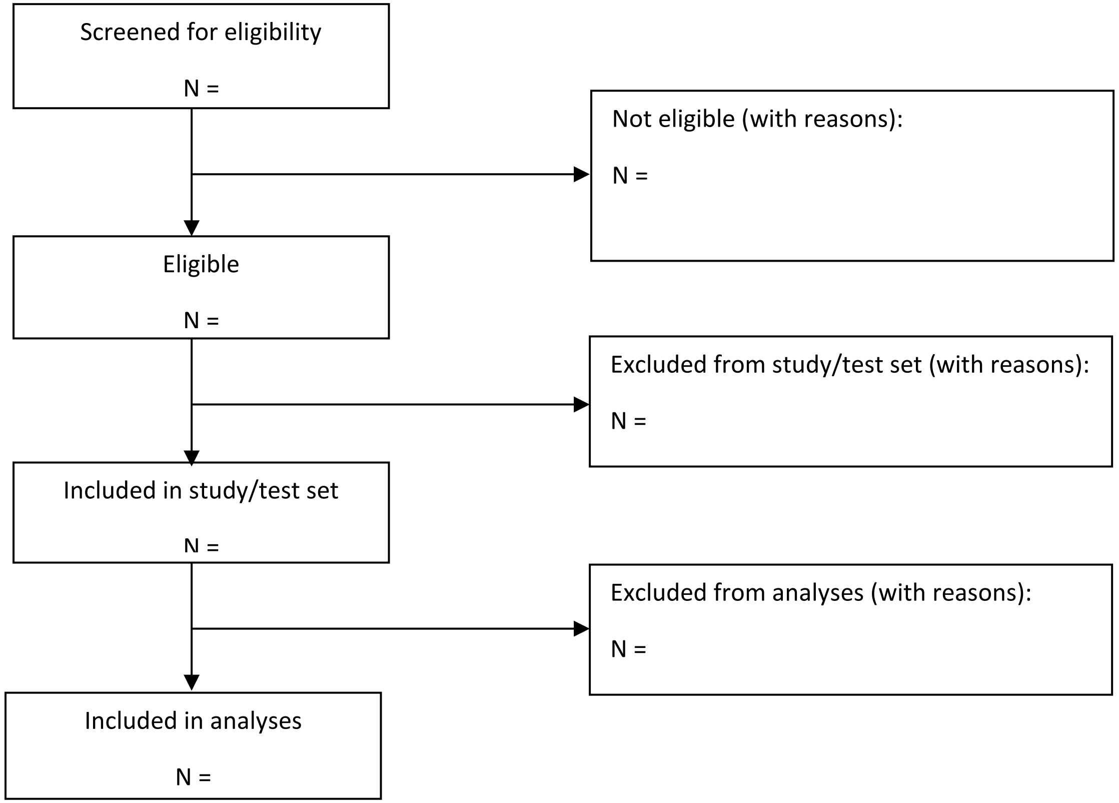

Phase 3: Risk of bias and applicability judgments

QUADAS-2 is structured so that 4 key domains are each rated in terms of the risk of bias and the concern regarding applicability to the research question (as defined above). Each key domain has a set of signalling questions to help reach the judgments regarding bias and applicability.

**DOMAIN 1: PATIENT SELECTION**<br>**A. Risk of Bias**					
Describe methods of patient selection:					
**Single test accuracy (QUADAS-2)**	**Answers for Index test [A]**	**Answers for Index test [B]**	**Answers for Index test [C]**	**Answers for Index test [D]**	**Guidance**
1.1 Was a consecutive or random sample of patients enrolled?	Yes<br>No<br>Unclear<br>NA	Yes<br>No<br>Unclear<br>NA	Yes<br>No<br>Unclear<br>NA	Yes<br>No<br>Unclear<br>NA	Consecutive (e.g. ALL patients in a certain time period) or random sampling – yes.<br>If not stated – unclear.<br>Other studies (selected or enriched sample) – no.
1.2 Was a case-control design avoided?	Yes<br>No<br>Unclear<br>NA	Yes<br>No<br>Unclear<br>NA	Yes<br>No<br>Unclear<br>NA	Yes<br>No<br>Unclear<br>NA	Studies with single set of inclusion criteria for study admission (1-gate); can be prospective or retrospective sampling – yes.<br>If not stated – unclear.<br>Studies with separate sampling schemes for diseased (cases) and non-diseased individuals (controls) (2-gate), e.g. if the samples are selected according to knowing whether people do or do not have lung nodules or lung cancer – no.
1.3 Did the study avoid inappropriate exclusions?	Yes<br>No<br>Unclear<br>NA	Yes<br>No<br>Unclear<br>NA	Yes<br>No<br>Unclear<br>NA	Yes<br>No<br>Unclear<br>NA	Use this to flag up that groups of people / CT images were systematically excluded who should not have been as their exclusion narrows the spectrum of diseased or non-diseased (e.g. exclusion of ‘easy to diagnose’ or ‘difficult to diagnose’ patients).<br>Systematic exclusion of CT images that could not be processed by the software (e.g. segmentation failures), even if reported in the paper as ‘Exclusions from the study’, should be ignored in this domain but scored in the ‘Flow & timing’ domain.<br>If nothing is said and consecutive or random sampling – yes.<br>If non-consecutive sampling issue and nothing said – unclear.<br>Exclusions by nodule number per image or unjustified (not based on management guidelines or minimal software manufacturer threshold) exclusion of certain nodule sizes) – no.

<table>
<tr><td></td><td></td><td></td><td></td><td></td><td>Systematic exclusion of patients with other non-nodule related lung pathology that can mimic or mask lung nodules (‘difficult to read’ CT images; e.g. severe pulmonary fibrosis, diffuse bronchiectasis, extensive inflammatory consolidation, pneumothorax, and massive pleural effusion) – no.
Systematic exclusion of ‘easy to read’ CT images (e.g. patients without other, non-nodule related lung conditions). – no.</td></tr>
<tr><td>1.4 Were the people/CT images included in the study independent of those used to train the AI algorithm?</td><td>Yes
No
Unclear
NA</td><td>Yes
No
Unclear
NA</td><td>Yes
No
Unclear
NA</td><td>Yes
No
Unclear
NA</td><td><u>For test set studies</u>, this translates as “Has the test set been clearly described as an external (geographically) validation set?”
Any internal validation (e.g. split sample, cross-validation) or temporal validation – no.
No details stated about the training set and tuning set - unclear.
External geographical validation (Test set was sample from a different centre; can be in another country or the same country) – yes.
For index test [D] without AI software involvement – NA.

<u>For prospective applied studies in a clinical context</u>:
If the study is located at different centre(s) to those who provided CT images used to train and tune the AI algorithm – yes.
If not stated – unclear.
If there is any overlap in patients or CT images – no.
For index test [D] without AI software involvement – NA.</td></tr>
<tr><td>1.5 Could the selection of patients have introduced bias?
(Score HIGH if ‘no’ to any question.)</td><td>RISK:
LOW
HIGH
UNCLEAR
NA</td><td>RISK:
LOW
HIGH
UNCLEAR
NA</td><td>RISK:
LOW
HIGH
UNCLEAR
NA</td><td>RISK:
LOW
HIGH
UNCLEAR
NA</td><td>All signalling questions answered with ‘yes’ – LOW.
At least one signalling question answered with ‘no’ – HIGH.
Only ‘yes’ and ‘unclear’ answers – UNCLEAR.</td></tr>
<tr><td>Comparative accuracy (QUADAS-C)</td><td colspan="2">Answers for the test comparison</td><td colspan="3">Guidance</td></tr>
<tr><td>C1.1 Was the risk of bias for each index test judged ‘low’ for this domain?</td><td colspan="2">Yes
No</td><td colspan="3">‘yes’ if the risk of bias judgment for single test accuracy (question 1.5 in QUADAS-2) was ‘low’ for each index test.</td></tr>
<tr><td>C1.2 Was a fully paired or randomized design used?</td><td colspan="2">Yes
No
Unclear</td><td colspan="3">‘yes’ if one of the following methods was used for allocating patients to index tests:
(1) each patient receiving all of the index tests (fully paired design) or
(2) random allocation of patients to one of the index tests (randomized design).</td></tr>
</table>

C1.3 Was the allocation sequence random?	Yes<br>No<br>Unclear<br>NA	Only applicable to randomized designs<br>‘yes’ if the study generated a truly random allocation sequence, for example, computer-generated random numbers and random number tables.
C1.4 Was the allocation sequence concealed until patients were enrolled and assigned to index tests?	Yes<br>No<br>Unclear<br>NA	Only applicable to randomized designs<br>‘yes’ if the study used appropriate methods to conceal allocation, such as central randomization schemes and opaque sealed envelopes.
**C1.5 Could the selection of patients have introduced bias in the comparison?**	**RISK:<br>LOW<br>HIGH<br>UNCLEAR**	Risk of bias can be judged ‘low’ if questions C1.1 to C1.4 were answered ‘yes’ (questions C1.3 and C1.4 are only applicable to randomized designs).<br>If at least one question was answered ‘no’, users should consider a ‘high risk of bias’ judgment if the bias associated with the design feature is of such concern that the entire domain is deemed problematic.<br>If C1.2 was answered ‘no’, strongly consider ‘high risk of bias’.
**B. Concerns regarding applicability**		
Describe included patients (prior testing, presentation, intended use of index test and setting)**:**		

**Single test accuracy (QUADAS-2)**	**Answers for Index test [A]**	**Answers for Index test [B]**	**Answers for Index test [C]**	**Answers for Index test [D]**	**Guidance**
**Please fill in one of the following four rows based on the assessed population (Incidental, Symptomatic, Screening, Surveillance)**					
**Is there concern that the included patients *(SCREENING)* do not match the review question?**	**CONCERN:<br>LOW<br>HIGH<br>UNCLEAR<br>NA**	**CONCERN:<br>LOW<br>HIGH<br>UNCLEAR<br>NA**	**CONCERN:<br>LOW<br>HIGH<br>UNCLEAR<br>NA**	**CONCERN:<br>LOW<br>HIGH<br>UNCLEAR<br>NA**	High concerns if:<br>- Not a consecutive or random sample of patients / CT images;<br>- Enriched sample (e.g. in-/exclusion by nodule number, nodule type and nodule size, respectively);<br>- Age not between 50-75 years;<br>- Not at high risk for lung cancer (e.g. current or former smokers, identified by questionnaire or other risk prediction model);<br>- Patients not representative of European screening population (study not performed in a European country);<br>- >10% of included people have a different indication for the CT scan than lung cancer screening;

					- CT image acquisition details different to European practice for a screening population (UK practice: slice thickness ≤2.0mm, low dose [<2 less mSV per scan], no contrast).

DOMAIN 2: INDEX TEST(S)

If more than one index test (e.g. different functions of the software) or a human comparator was used, please complete for each test.

A. Risk of Bias

Describe the index test and how it was conducted and interpreted:

Single test accuracy (QUADAS-2)	Answers for Index test [A]	Answers for Index test [B]	Answers for Index test [C]	Answers for Index test [D]	Guidance
2.1 Were the index test results interpreted without knowledge of the results of the reference standard?<br>(Requires no repeated application of AI to any of the same CT images, or use of the same CT images or images from the same patients for training)	Yes<br>No<br>Unclear<br>NA	Yes<br>No<br>Unclear<br>NA	Yes<br>No<br>Unclear<br>NA	Yes<br>No<br>Unclear<br>NA	[A] For index tests where AI software is used standalone (<u>without any human element):</u><br>- AI system has not previously been trained on these CT images or learned from these CT images or other CT images from the same patients – yes.<br>- If data from the same dataset was used for training/tuning the software – no.<br>- If repeat use of the same CT images or other CT images from the same patients within the same or previous studies – no (unless explicit that the AI algorithm was pre-set and did not change upon repeat use, and the study did not select one of several AI systems based on use with the same cases).<br>- If nothing is said about training/tuning – unclear.<br>- If not explicit that there has been no repeat use within the same or previous studies – unclear.<br><br>[B] [C] [D] For index tests <u>where a human is involved</u> (either unassisted human read comparator, software-assisted human readers e.g. second-read CAD or concurrent CAD):

					- Requires clear statement of blinding, or clear temporal relationships where the human read occurred before the reference standard – yes.<br>- If nothing is said and no clear temporal relationship – unclear.<br>- If clearly unblinded – no.
2.2 If a threshold was used, was it pre-specified?	Yes<br>No<br>Unclear<br>NA	Yes<br>No<br>Unclear<br>NA	Yes<br>No<br>Unclear<br>NA	Yes<br>No<br>Unclear<br>NA	[A] If the AI software threshold was pre-set by company or clearly pre-specified in methods (e.g. sensitivity and/or FP rate threshold or nodule size threshold) – yes.<br>If AI software threshold clearly not pre-set by company or pre-specified in methods – no.<br>Using sensitivity / specificity of the unaided reader as benchmark using the same dataset – no.<br>Reporting AI software performance at various threshold settings or in a ROC curve – no.<br>If nothing is said – unclear.<br>No threshold used – NA.<br><br>[B] [C] [D] Unaided or software-assisted human readers detecting nodules:<br>Use of a pre-specified nodule size or volume threshold – yes.<br>If a threshold is used but it is unclear if it was pre-specified – unclear.<br>Nodule size or volume threshold not pre-specified – no.<br>No threshold used – NA.
2.3 Where human readers are part of the test, were their decisions made in a clinical practice context? (i.e. avoidance of the laboratory effect)	Yes<br>No<br>Unclear<br>NA	Yes<br>No<br>Unclear<br>NA	Yes<br>No<br>Unclear<br>NA	Yes<br>No<br>Unclear<br>NA	This question has been added.<br>[A] NA<br>[B] [C] [D] If the readers made decisions in the clinical context, and those decisions were used to decide whether to discharge or recall patients (either prospectively as part of a trial or test accuracy study or retrospective studies using the original decision) – yes.<br><br>If readers examined a test set (of any prevalence) outside clinical practice, or any other context likely to result in the laboratory effect (that their reading result is not influencing a patient's diagnosis) – no.
**2.4 Could the conduct or interpretation of the index test have introduced bias?**	**RISK:<br>LOW<br>HIGH<br>UNCLEAR<br>NA**	**RISK:<br>LOW<br>HIGH<br>UNCLEAR<br>NA**	**RISK:<br>LOW<br>HIGH<br>UNCLEAR<br>NA**	**RISK:<br>LOW<br>HIGH<br>UNCLEAR<br>NA**	All signalling questions answered with 'yes' – LOW.<br>At least one signalling question answered with 'no' – HIGH<br>Only 'yes' and 'unclear' answers – UNCLEAR.

**Comparative accuracy (QUADAS-C)**	**Answers for the test comparison**			**Guidance**	
C2.1 Was the risk of bias for each index test judged 'low' for this domain?	Yes<br>No			'yes' if the risk of bias judgment for single test accuracy (question 2.5 in QUADAS-2) was 'low' for each index test.	
C2.2 Were the index test results interpreted without knowledge of the results of the other index test(s)?	Yes<br>No<br>Unclear<br>NA			Only applicable if patients received multiple index tests (fully or partially paired designs)<br>'yes' if index test [A] was interpreted blind to the results of index test [B] and vice versa.<br>Blinding is not necessary if none of the index tests involve subjective interpretation.	
C2.3 Is undergoing one index test unlikely to affect the performance of the other index test(s)?	Yes<br>No<br>Unclear<br>NA			Only applicable if patients received multiple index tests (fully or partially paired designs)<br>'yes' if one index test cannot influence or interfere with the results of subsequently performed index test(s).<br>Examples of such influence or interference include distortion of sampling area (biopsies) and patient fatigue (questionnaires).	
C2.4 Were the index tests conducted and interpreted without advantaging one of the tests?	Yes<br>No<br>Unclear			'yes' if there were no differences in the conduct and interpretation between the index tests that may unfairly benefit one of the tests. An example of such a difference is when index test A was performed by an expert and index test B by a nonexpert.<br>Differences between tests that reflect clinical practice may be acceptable, in which case 'yes' is appropriate.	
**C2.5 Could the conduct or interpretation of the index tests have introduced bias in the comparison?**<br>**(Score HIGH if 'no' to any question.)**	**RISK:**<br>**LOW**<br>**HIGH**<br>**UNCLEAR**			Risk of bias can be judged 'low' if signaling questions C2.1 to C2.4 were answered 'yes' (C2.2 and C2.3 are only applicable to fully or partially paired designs).<br>If at least one question was answered 'no', users should consider a 'high risk of bias' judgment if the bias associated with the design feature is of such concern that the entire domain is deemed problematic.	
**B. Concerns regarding applicability**					
**Single test accuracy (QUADAS-2)**	**Answers for Index test [A]**	**Answers for Index test [B]**	**Answers for Index test [C]**	**Answers for Index test [D]**	**Guidance**
**Is there concern that the index test(s) or comparator, its conduct, or interpretation differ from the review question?**	**CONCERN:**<br>**LOW**<br>**HIGH**<br>**UNCLEAR**<br>**NA**	**CONCERN:**<br>**LOW**<br>**HIGH**<br>**UNCLEAR**<br>**NA**	**CONCERN:**<br>**LOW**<br>**HIGH**<br>**UNCLEAR**<br>**NA**	**CONCERN:**<br>**LOW**<br>**HIGH**<br>**UNCLEAR**<br>**NA**	High concerns if:<br>For all functionalities:<br>- [A] [B] [C] Any prototype versions that did not later become the commercially available version (e.g. applicability not confirmed by the company).<br>- Integration of software into pathway not applicable to UK or EU

					(e.g. standalone AI performance [A] instead of concurrent [C] or second-read [B] CAD; for [B] and [C] – more than 1 human reader involved per read);<br>- Human comparator [D] not applicable to UK or European practice (e.g. human double reading instead of single human reader);<br>- Human reader's experience and/or specialty not representative of UK or European clinical practice (The training for radiologists is 5 years. After that time they are considered "fully trained".) for this target population;<br>- [B] [C] [D] Reader had no access to maximum intensity projections (MIP) and/or multiplanar reformations (MPR).<br><u>Nodule detection:</u><br>- Study did not use a pre-specified nodule size threshold similar to the UK 2015 BTS guidelines (i.e. ≥5mm maximum axial diameter or ≥80mm^3), Lung-RADS or the European Position Statement (EUPS) (i.e. ≥5mm diameter or ≥100mm^3).<br>- [A] CAD false positive rate set to >2 per case.<br><u>Nodule type determination:</u><br>- Other nodule types used than in the UK BTS guidelines, Lung-RADS or the European Position Statement (EUPS) (nodule type should be classified as solid, part-solid or pure ground glass nodules).<br><u>Nodule size measurement (volume/diameter):</u><br>- Nodules should be measured using semi-automated volumetry. Where volumetry segmentation is not possible or judged to be inaccurate, maximal axial manual diameter measurements should be recorded, excluding any spiculation. Manual adjustment of volumetric analysis should be avoided as this may introduce unquantified variability.

**DOMAIN 3: REFERENCE STANDARD**<br>**A. Risk of Bias**					
Describe the reference standard and how it was conducted and interpreted:					
**Single test accuracy (QUADAS-2)**	**Answers for Index test [A]**	**Answers for Index test [B]**	**Answers for Index test [C]**	**Answers for Index test [D]**	**Guidance**

3.1 Is the reference standard likely to correctly classify the target condition?	Yes<br>No<br>Unclear<br>NA	Yes<br>No<br>Unclear<br>NA	Yes<br>No<br>Unclear<br>NA	Yes<br>No<br>Unclear<br>NA	Lung cancer:<br>Histopathology after biopsy/excision – yes.<br>Medical records – no.<br><br>Benign nodules:<br>Histopathology after biopsy/excision;<br><u>For solid nodules:</u> CT surveillance for at least 2 years with stable diameter or stable (or VDT>600 days) after 1 year on volumetry;<br><u>For subsolid nodules:</u> resolved at CT scan after 3 months or CT surveillance for at least 4 years without growth or altered morphology;<br>At least 2 year follow-up without lung cancer diagnosis – yes.<br><br>Nodule detection / nodule type / nodule pairs;<br>No reference standard in in vivo studies: will accept majority or consensus reading of (at least) 3 experienced thoracic – yes.<br>Less than 3 experienced thoracic radiologist – no.<br><br>Nodule size:<br>Measurement of nodule size after nodule excision or consensus/average size measurement of (at least) 3 experienced thoracic radiologists – yes.
3.2 Were the reference standard results interpreted without knowledge of the results of the index test?	Yes<br>No<br>Unclear<br>NA	Yes<br>No<br>Unclear<br>NA	Yes<br>No<br>Unclear<br>NA	Yes<br>No<br>Unclear<br>NA	Malignant / benign nodules:<br>For retrospective studies if the original human reader is used as comparator test – no.<br>For prospective studies if the investigators did not blind the clinicians undertaking the follow up tests to which index test examined the CT images - no.<br>For retrospective studies where readers read CT scans prospectively (reader study) – yes.<br><br>Nodule detection / nodule type / nodule pairs / nodule size:<br>If the reference standard reader(s) performed their read prior to the index test(s) – yes.<br>If reference standard reader(s) are blinded to AI and human reader results – yes.<br>If reference standard reader(s) are part of the index test(s) or not blinded to index test markings / decisions – no.
**3.3 Could the reference standard, its conduct, or its**	**RISK: LOW**	**RISK: LOW**	**RISK: LOW**	**RISK: LOW**	All signalling questions answered with ‘yes’ – LOW.<br>At least one signalling question answered with ‘no’ – HIGH.

**interpretation have introduced bias?**	**HIGH**<br>**UNCLEAR**<br>**NA**	**HIGH**<br>**UNCLEAR**<br>**NA**	**HIGH**<br>**UNCLEAR**<br>**NA**	**HIGH**<br>**UNCLEAR**<br>**NA**	Only 'yes' and 'unclear' answers – UNCLEAR.
**Comparative accuracy (QUADAS-C)**	**Answers for the test comparison**				**Guidance**
C3.1 Was the risk of bias for each index test judged 'low' for this domain?	Yes<br>No				'yes' if the risk of bias judgment for single test accuracy (question 3.3 in QUADAS-2) was 'low' for each index test.
C3.2 Did the reference standard avoid incorporating any of the index tests?	Yes<br>No<br>Unclear				'Incorporation' means that an index test is part of the reference standard. This question is not about whether the reference standard results were interpreted without knowledge of the index test results.<br>'yes' if none of the index tests were part of the reference standard. Note that this issue is different from blinding (signaling question 3.2 in QUADAS-2).
**C3.3 Could the reference standard, its conduct, or its interpretation have introduced bias in the comparison?**	**RISK:**<br>**LOW**<br>**HIGH**<br>**UNCLEAR**				Risk of bias can be judged 'low' if signaling questions C3.1 and C3.2 were answered 'yes'. If at least one question was answered 'no', users should consider a 'high risk of bias' judgment if the bias associated with the design feature is of such concern that the entire domain is deemed problematic.
**B. Concerns regarding applicability**					
**Single test accuracy (QUADAS-2)**	**Answers for Index test [A]**	**Answers for Index test [B]**	**Answers for Index test [C]**	**Answers for Index test [D]**	**Guidance**
**Is there concern that the target condition as defined by the reference standard does not match the review question?**	**CONCERN:**<br>**LOW**<br>**HIGH**<br>**UNCLEAR**<br>**NA**	**CONCERN:**<br>**LOW**<br>**HIGH**<br>**UNCLEAR**<br>**NA**	**CONCERN:**<br>**LOW**<br>**HIGH**<br>**UNCLEAR**<br>**NA**	**CONCERN:**<br>**LOW**<br>**HIGH**<br>**UNCLEAR**<br>**NA**	High concerns if:<br>Malignant/benign nodules:<br>- Different length of CT surveillance (e.g. solid nodules: <2 years with diameter measurements or <1 year with volume measurements; non-resolved sub-solid nodules <4 years);<br>- Diagnosis of cancer not by pathology of biopsied/resected nodules;<br>- No follow-up for at least two years for patients with nodules who are not receiving CT surveillance or biopsy/excision.<br>"Actionable" nodule present/absent:<br>- Different nodule size to BTS 2015 guideline definition ("actionable nodule" is ≥5 mm maximum axial diameter or ≥80 mm^3), Lung-RADS or the European Position Statement (EUPS) (i.e. ≥5mm diameter or ≥100mm^3).<br><br>Nodule type:<br>- Other types used than in the BTS 2015 guidelines, Lung-RADS or the European Position Statement (EUPS) (nodule type should be classified as solid, part-solid or pure ground

					glass nodules).<br><br><u>Nodule size measurement (volume/diameter):</u><br>- Nodule size should be measured as volume or, if volumetry segmentation is not possible, as maximum axial diameter.<br><br><u>Nodule pairs:</u><br>- NA

**DOMAIN 4: FLOW AND TIMING**<br>**Risk of Bias**					
Describe any patients who did not receive the index test(s) and/or reference standard or who were excluded from the 2x2 table (refer to flow diagram):					
Describe the time interval and any intervention between index tests(s) and reference standard:					
**Single test accuracy (QUADAS-2)**	**Answers for Index test [A]**	**Answers for Index test [B]**	**Answers for Index test [C]**	**Answers for Index test [D]**	**Guidance**
4.1 Did all patients receive a reference standard?	Yes<br>No<br>Unclear<br>NA	Yes<br>No<br>Unclear<br>NA	Yes<br>No<br>Unclear<br>NA	Yes<br>No<br>Unclear<br>NA	<u>Malignant / benign nodules:</u><br>If any patients who should have received a biopsy/resection, other follow-up tests and/or CT surveillance after index test positive results did not receive one or results were unavailable – no.<br>If index test negative patients were not followed up for at least one year (pragmatic threshold) to confirm absence of lung cancer – no.<br><br><u>Nodule detection / nodule type / detection of nodule pairs:</u><br>If ALL CT images are assessed by expert reading as reference standard - yes.
4.2 Did all patients receive the same reference standard?	Yes<br>No<br>Unclear	Yes<br>No<br>Unclear	Yes<br>No<br>Unclear	Yes<br>No<br>Unclear	Need to give separate answers for detection of lung cancer, nodule detection, nodule composition or detection of nodule pairs.

	NA	NA	NA	NA	<u>For nodule detection, nodule composition, detection of nodule pairs:</u><br>If all CT images received the SAME reference standard (e.g. consensus expert reading) - yes.<br><br><u>Malignant / benign nodules:</u><br>Usually NO – all studies will necessarily have differential verification, because not all patients can or should be biopsied.
4.3 Were all patients included in the analysis?	Yes<br>No<br>Unclear<br>NA	Yes<br>No<br>Unclear<br>NA	Yes<br>No<br>Unclear<br>NA	Yes<br>No<br>Unclear<br>NA	If there were significant exclusions (>10%; cut-off determined pragmatically) after the point of selecting the cohort, for example indeterminate results (e.g. segmentation failures) or losses to follow up – no.<br>If the number of excluded CT images after the point of selecting the test set / study sample is not reported – unclear.<br>If there were <10% of CT images excluded from the analyses – yes.
4.4 If there were exclusions from the analysis, has it been reported how many were due to software processing failures?	Yes<br>No<br>Unclear<br>NA	Yes<br>No<br>Unclear<br>NA	Yes<br>No<br>Unclear<br>NA	Yes<br>No<br>Unclear<br>NA	This signalling question was added.<br>If the number of CT images excluded due to software processing failures (e.g. segmentation failures) has been reported – yes.<br>If it is unclear if there were any exclusions from the analysis – unclear.<br>If the number of CT images excluded due to software processing failures (e.g. segmentation failures) has not been reported – no.<br>Unaided readers [D] or no exclusions from the analysis – NA.
**4.5 Could the patient flow have introduced bias?**	**RISK:<br>LOW<br>HIGH<br>UNCLEAR<br>NA**	**RISK:<br>LOW<br>HIGH<br>UNCLEAR<br>NA**	**RISK:<br>LOW<br>HIGH<br>UNCLEAR<br>NA**	**RISK:<br>LOW<br>HIGH<br>UNCLEAR<br>NA**	All signalling questions answered with 'yes' – LOW.<br>At least one signalling question answered with 'no' – HIGH.<br>Only 'yes' and 'unclear' answers – UNCLEAR.
**Comparative accuracy (QUADAS-C)**	**Answers for the test comparison**		**Guidance**		
C4.1 Was the risk of bias for each index test judged 'low' for this domain?	Yes<br>No		'yes' if the risk of bias judgment for single test accuracy (question 4.5 in QUADAS-2) was 'low' for each index test.		
C4.2 Was there an appropriate interval between the index tests?	Yes<br>No<br>Unclear		For many index tests, 'appropriate' would constitute performing the tests at the same time after patient enrolment. This excludes the possibility of disease progression or change in patient management. Some index tests have different 'diagnostic windows' and are ideally performed at different timepoints; subject-matter expertise is required to determine this.		
C4.3 Was the same reference standard used for all index tests?	Yes<br>No		'yes' if either (1) a single reference standard was used in all patients or (2) multiple reference standards were used (e.g., either surgery or follow-up) and these reference standards were the same for patients receiving index		

	Unclear	test [A] and patients receiving index test [B].
C4.4 Are the proportions and reasons for missing data similar across index tests?	Yes<br>No<br>Unclear	Missing data occurs if test results are unavailable, invalid, inconclusive, or if patients are excluded from the analysis.<br>‘yes’ if there is no missing data, or if the proportion and reasons for missing data are similar for index test [A] and index test [B].
**C4.5 Could the patient flow have introduced bias in the comparison?**	**RISK:**<br>**LOW**<br>**HIGH**<br>**UNCLEAR**	Risk of bias can be judged ‘low’ if signaling questions C4.1 to C4.4 were answered ‘yes’. If at least one question was answered ‘no’, users should consider a ‘high risk of bias’ judgment if the bias associated with the design feature is of such concern that the entire domain is deemed problematic.

Supplementary material 4: PRISMA diagram. Summary of publications included and excluded at each stage of the review (original searches / update searches)

NA, Not applicable; ND, Not done.

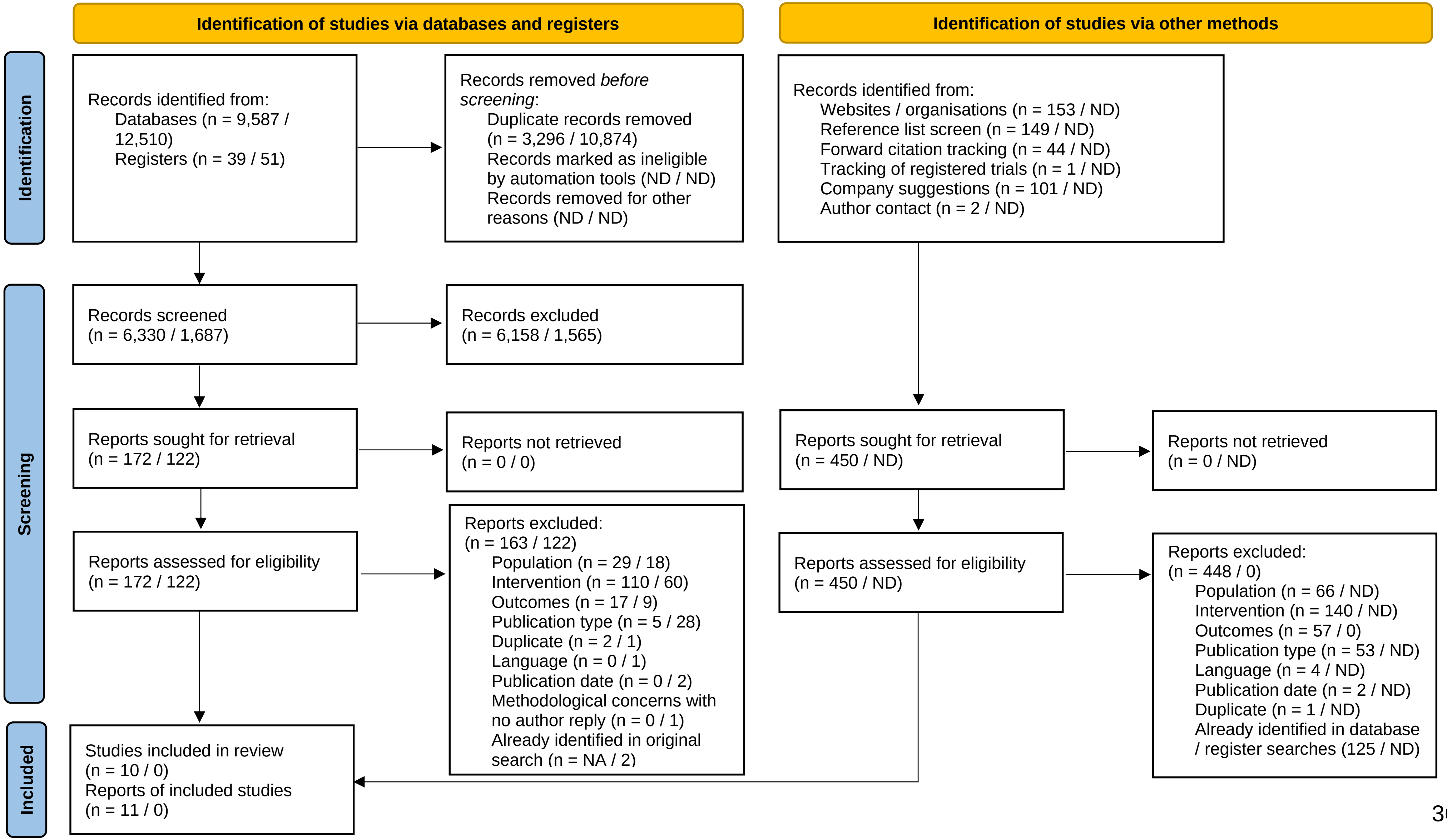

Supplementary material 5: Publications and sub-studies excluded after review of full-text articles

Key to reasons for exclusions and justifications

Population – >10% of CT images taken for reasons other than lung cancer screening; use of chest phantoms; other image type than computed tomography (CT).

Intervention – Language processing tool; malignancy risk prediction; software not commercially available; no CE mark by December 2021; software not AI-based; software name unclear and no author reply;

Outcomes - No relevant outcomes reported; clinical trial register with no outcomes reported yet.

Publication type – Conference abstract with no additional data reported to an already included full journal paper; conference abstract without accompanying full journal article; no primary research article (e.g. reviews, editorials).

Publication date – Records published prior 2012 excluded from the original searches; records published prior 2022 were excluded from the update searches.

Language – Records not available in English language.

Publications excluded with reasons

A. Publications excluded after review of full-text articles – Original electronic database searches (n=163)

Reference	Main reason for exclusion
**Excluded on population: <90% screening LDCT (n=20)**	
1. Abadia AF, Yacoub B, Stringer N, et al. Diagnostic Accuracy and Performance of Artificial Intelligence in Detecting Lung Nodules in Patients With Complex Lung Disease: A Noninferiority Study. J Thorac Imaging 2021;12:12. doi: https://dx.doi.org/10.1097/RTI.0000000000000613	<90% screening LDCT
2. Ahn Y, Lee SM, Noh HN, et al. Use of a Commercially Available Deep Learning Algorithm to Measure the Solid Portions of Lung Cancer Manifesting as Subsolid Lesions at CT: Comparisons with Radiologists and Invasive Component Size at Pathologic Examination. Radiology 2021;299(1):202-10. doi: https://dx.doi.org/10.1148/radiol.2021202803	<90% screening LDCT
3. Blazis SP, Dickerscheid DBM, Linsen PVM, et al. Effect of CT reconstruction settings on the performance of a deep learning based lung nodule CAD system. Eur J Radiol 2021;136:109526. doi: https://dx.doi.org/10.1016/j.ejrad.2021.109526	<90% screening LDCT
4. Cohen JG, Kim H, Park SB, et al. Comparison of the effects of model-based iterative reconstruction and filtered back projection algorithms on software measurements in pulmonary subsolid nodules. Eur Radiol 2017;27(8):3266-74. doi: https://dx.doi.org/10.1007/s00330-016-4716-5	<90% screening LDCT
5. Kim H, Park CM, Hwang EJ, et al. Pulmonary subsolid nodules: value of semi-automatic measurement in diagnostic accuracy, diagnostic reproducibility and nodule classification agreement. Eur Radiol 2018;28(5):2124-33. doi: https://dx.doi.org/10.1007/s00330-017-5171-7	<90% screening LDCT
6. Kozuka T, Matsukubo Y, Kadoba T, et al. Efficiency of a computer-aided diagnosis (CAD) system with deep learning in detection of pulmonary nodules on 1-mm-thick images of computed tomography. Jpn J Radiol 2020;38(11):1052-61. doi: https://dx.doi.org/10.1007/s11604-020-01009-0	<90% screening LDCT
7. Liu K, Li Q, Ma J, et al. Evaluating a Fully Automated Pulmonary Nodule Detection Approach and Its Impact on Radiologist Performance. Radiol Artif Intell 2019;1(3):e180084. doi: https://dx.doi.org/10.1148/ryai.2019180084	<90% screening LDCT
8. Martini K, Bluthgen C, Eberhard M, et al. Impact of Vessel Suppressed-CT on Diagnostic Accuracy in Detection of Pulmonary Metastasis and Reading Time. Acad Radiol 2021;28(7):988-94. doi: https://dx.doi.org/10.1016/j.acra.2020.01.014	<90% screening LDCT
9. Martins Jarnalo CO, Linsen PVM, Blazis SP, et al. Clinical evaluation of a deep-learning-based computer-aided detection system for the detection of pulmonary nodules in a large teaching hospital. Clin Radiol 2021;76(11):838-45. doi: https://dx.doi.org/10.1016/j.crad.2021.07.012	<90% screening LDCT
10. Meybaum C, Graff M, Fallenberg EM, et al. Contribution of CAD to the Sensitivity for Detecting Lung Metastases on Thin-Section CT - A Prospective Study with Surgical and Histopathological Correlation. ROFO Fortschr Geb Rontgenstr Nuklearmed 2020;192(1):65-73. doi: https://dx.doi.org/10.1055/a-0977-3453	<90% screening LDCT
11. Park S, Lee SM, Kim W, et al. Computer-aided Detection of Subsolid Nodules at Chest CT: Improved Performance with Deep Learning-based CT Section Thickness Reduction. Radiology 2021;299(1):211-19. doi: https://dx.doi.org/10.1148/radiol.2021203387	<90% screening LDCT
12. Rueckel J, Sperl JI, Kaestle S, et al. Reduction of missed thoracic findings in emergency whole-body computed tomography using artificial intelligence assistance. Quant 2021;11(6):2486-98. doi: https://dx.doi.org/10.21037/qims-20-1037	<90% screening LDCT
13. Shaffer K. Deep Learning and Lung Cancer: AI to Extract Information Hidden in Routine CT Scans. Radiology 2020;296(1):225-26. doi: https://dx.doi.org/10.1148/radiol.2020201366	<90% screening LDCT

Reference	Main reason for exclusion
14. Takaishi T, Ozawa Y, Bando Y, et al. Incorporation of a computer-aided vessel-suppression system to detect lung nodules in CT images: effect on sensitivity and reading time in routine clinical settings. Jpn J Radiol 2021;39(2):159-64. doi: https://dx.doi.org/10.1007/s11604-020-01043-y	<90% screening LDCT
15. Vassallo L, Traverso A, Agnello M, et al. A cloud-based computer-aided detection system improves identification of lung nodules on computed tomography scans of patients with extra-thoracic malignancies. Eur Radiol 2019;29(1):144-52. doi: https://dx.doi.org/10.1007/s00330-018-5528-6	<90% screening LDCT
16. Wagner AK, Hapich A, Psychogios MN, et al. Computer-Aided Detection of Pulmonary Nodules in Computed Tomography Using ClearReadCT. J Med Syst 2019;43(3):58. doi: https://dx.doi.org/10.1007/s10916-019-1180-1	<90% screening LDCT
17. Wan Y-L, Pan K-T, Wu PW, et al. The use of artificial intelligence in the differentiation of malignant and benign lung nodules on computed tomograms proven by surgical pathology. Cancers (Basel) 2020;12(8):1-14. doi: http://dx.doi.org/10.3390/cancers12082211	<90% screening LDCT
18. Weikert T, Akinci D'Antonoli T, Bremerich J, et al. Evaluation of an AI-Powered Lung Nodule Algorithm for Detection and 3D Segmentation of Primary Lung Tumors. Contrast Media Mol Imaging 2019;2019:1545747. doi: https://dx.doi.org/10.1155/2019/1545747	<90% screening LDCT
19. Yacoub B, Kabakus I, Schoepf J, et al. Performance of an Artificial Intelligence-Based Platform Against Clinical Radiology Reports for the Evaluation of Non-contrast Chest CT. J Thorac Imaging 2021;36(6):W123. doi: http://dx.doi.org/10.1097/RTI.0000000000000619	<90% screening LDCT
20. Yacoub B, Kabakus IM, Schoepf UJ, et al. Performance of an Artificial Intelligence-Based Platform Against Clinical Radiology Reports for the Evaluation of Noncontrast Chest CT. Acad Radiol 2021;10:10. doi: https://dx.doi.org/10.1016/j.acra.2021.02.007	<90% screening LDCT
**Excluded on population: Chest phantoms (n=3)**	
21. Ebner L, Roos JE, Christensen JD, et al. Maximum-Intensity-Projection and Computer-Aided-Detection Algorithms as Stand-Alone Reader Devices in Lung Cancer Screening Using Different Dose Levels and Reconstruction Kernels. AJR Am J Roentgenol 2016;207(2):282-8. doi: https://dx.doi.org/10.2214/AJR.15.15588	Chest phantom
22. Peters AA, Decasper A, Munz J, et al. Performance of an AI based CAD system in solid lung nodule detection on chest phantom radiographs compared to radiology residents and fellow radiologists. J 2021;13(5):2728-37. doi: https://dx.doi.org/10.21037/jtd-20-3522	Chest phantom
23. Schwyzer M, Messerli M, Eberhard M, et al. Impact of dose reduction and iterative reconstruction algorithm on the detectability of pulmonary nodules by artificial intelligence. Diagn Interv Imaging 2022;03:03. doi: https://dx.doi.org/10.1016/j.diii.2021.12.002	Chest phantom
**Excluded on population: Other image type (n=6)**	
24. Lee JH, Sun HY, Park S, et al. Performance of a Deep Learning Algorithm Compared with Radiologic Interpretation for Lung Cancer Detection on Chest Radiographs in a Health Screening Population. Radiology 2020;297(3):687-96. doi: https://dx.doi.org/10.1148/radiol.2020201240	Other image type
25. Rajagopalan K, Babu S. The detection of lung cancer using massive artificial neural network based on soft tissue technique. BMC Med Inf Decis Mak 2020;20(1):282. doi: https://dx.doi.org/10.1186/s12911-020-01220-z	Other image type
26. Schultheiss M, Schmette P, Bodden J, et al. Lung nodule detection in chest X-rays using synthetic ground-truth data comparing CNN-based diagnosis to human performance. Sci 2021;11(1):15857. doi: https://dx.doi.org/10.1038/s41598-021-94750-z	Other image type
27. Ueda D, Yamamoto A, Shimazaki A, et al. Artificial intelligence-supported lung cancer detection by multi-institutional readers with multi-vendor chest radiographs: a retrospective clinical validation study. BMC Cancer 2021;21(1):1120. doi: https://dx.doi.org/10.1186/s12885-021-08847-9	Other image type
28. Yamada Y, Shiomi E, Hashimoto M, et al. Value of a Computer-aided Detection System Based on Chest Tomosynthesis Imaging for the Detection of Pulmonary Nodules. Radiology 2018;287(1):333-39. doi: https://dx.doi.org/10.1148/radiol.2017170405	Other image type
29. Yoo H, Lee SH, Arru CD, et al. AI-based improvement in lung cancer detection on chest radiographs: results of a multi-reader study in NLST dataset. Eur Radiol 2021;31(12):9664-74. doi: https://dx.doi.org/10.1007/s00330-021-08074-7	Other image type

Reference	Main reason for exclusion
**Excluded on technology: Language processing tool (n=1)**	
30. Hunter B, Reis S, Campbell D, et al. Development of a Structured Query Language and Natural Language Processing Algorithm to Identify Lung Nodules in a Cancer Centre. Front Med (Lausanne) 2021;8:748168. doi: https://dx.doi.org/10.3389/fmed.2021.748168	Language processing tool
**Excluded on technology: Malignancy risk prediction (n=12)**	
31. Ardila D, Kiraly AP, Bharadwaj S, et al. End-to-end lung cancer screening with three-dimensional deep learning on low-dose chest computed tomography. Nat Med 2019;25(6):954-61. doi: https://dx.doi.org/10.1038/s41591-019-0447-x	Malignancy risk prediction
32. Heuvelmans MA, Oudkerk M. Deep learning to stratify lung nodules on annual follow-up CT. Lancet Digit Health 2019;1(7):e324-e25. doi: https://dx.doi.org/10.1016/S2589-7500(19)30156-6	Malignancy risk prediction
33. Huang P, Park S, Yan R, et al. Added Value of Computer-aided CT Image Features for Early Lung Cancer Diagnosis with Small Pulmonary Nodules: A Matched Case-Control Study. Radiology 2018;286(1):286-95. doi: https://dx.doi.org/10.1148/radiol.2017162725	Malignancy risk prediction
34. Jacobs C, Setio AAA, Scholten ET, et al. Deep Learning for Lung Cancer Detection on Screening CT Scans: Results of a Large-Scale Public Competition and an Observer Study with 11 Radiologists. Radiol Artif Intell 2021;3(6):e210027. doi: https://dx.doi.org/10.1148/ryai.2021210027	Malignancy risk prediction
35. Lassau N, Bousaid I, Chouzenoux E, et al. Three artificial intelligence data challenges based on CT and MRI. Diagn Interv Imaging 2020;101(12):783-88. doi: https://dx.doi.org/10.1016/j.diii.2020.03.006	Malignancy risk prediction
36. Pickup L, Arteta C, Declerck J, et al. P1.11-02 Acceleration of Lung Cancer Diagnosis: Utility Study for AI-Based Stratification of Pulmonary Nodules. Journal of Thoracic Oncology 2019;14(10 Supplement):S515. doi: http://dx.doi.org/10.1016/j.jtho.2019.08.1075	Malignancy risk prediction
37. Tsakok MT, Mashar M, Pickup L, et al. The utility of a convolutional neural network (CNN) model score for cancer risk in indeterminate small solid pulmonary nodules, compared to clinical practice according to British Thoracic Society guidelines. Eur J Radiol 2021;137:109553. doi: https://dx.doi.org/10.1016/j.ejrad.2021.109553	Malignancy risk prediction
38. Wels M, Lades F, Muehlberg A, et al. General Purpose Radiomics for Multi-Modal Clinical Research. Medical Imaging 2019: Computer-Aided Diagnosis 2019;10950 doi: 10.1117/12.2511856	Malignancy risk prediction
39. Xu T, Huang C, Liu Y, et al. Artificial intelligence based on deep learning for differential diagnosis between benign and malignant pulmonary nodules: A real-world, multicenter, diagnostic study. Journal of Clinical Oncology 2020;38(15) doi: https://dx.doi.org/10.1200/JCO.2020.38.15-suppl.9037	Malignancy risk prediction
40. Zeng JY, Ye HH, Yang SX, et al. Clinical application of a novel computer-aided detection system based on three-dimensional CT images on pulmonary nodule. Int J Clin Exp Med 2015;8(9):16077-82.	Malignancy risk prediction
41. Zhao L, Bai C, Zhu Y. Preliminary study on diagnostic value of artificial intelligence in early-stage lung cancer. American Journal of Respiratory and Critical Care Medicine 2020;201(1)	Malignancy risk prediction
42. Zhao L, Bai C-X, Zhu Y. Diagnostic value of artificial intelligence in early-stage lung cancer. Chin Med J 2020;133(4):503-04. doi: http://dx.doi.org/10.1097/CM9.0000000000000634	Malignancy risk prediction
**Excluded on technology: Software not commercially available (n=37)**	
43. Akter O, Moni MA, Islam MM, et al. Lung cancer detection using enhanced segmentation accuracy. Appl Intell 2021;51(6):3391-404. doi: 10.1007/s10489-020-02046-y	Software not commercially available
44. Aresta G, Jacobs C, Araujo T, et al. iW-Net: an automatic and minimalistic interactive lung nodule segmentation deep network. Sci 2019;9(1):11591. doi: https://dx.doi.org/10.1038/s41598-019-48004-8	Software not commercially available

Reference	Main reason for exclusion
45. Cui X, Zheng S, Heuvelmans MA, et al. Performance of a deep learning-based lung nodule detection system as an alternative reader in a Chinese lung cancer screening program. Eur J Radiol 2022;146:110068. doi: https://dx.doi.org/10.1016/j.ejrad.2021.110068	Software not commercially available
46. Huang W, Xue Y, Wu Y. A CAD system for pulmonary nodule prediction based on deep three-dimensional convolutional neural networks and ensemble learning. PLoS ONE 2019;14(7):e0219369. doi: https://dx.doi.org/10.1371/journal.pone.0219369	Software not commercially available
47. Iwasawa T, Matsumoto S, Aoki T, et al. A comparison of axial versus coronal image viewing in computer-aided detection of lung nodules on CT. Jpn J Radiol 2015;33(2):76-83. doi: https://dx.doi.org/10.1007/s11604-014-0383-0	Software not commercially available
48. Jacobs C, van Rikxoort EM, Scholten ET, et al. Solid, part-solid, or non-solid?: classification of pulmonary nodules in low-dose chest computed tomography by a computer-aided diagnosis system. Invest Radiol 2015;50(3):168-73. doi: https://dx.doi.org/10.1097/RLI.0000000000000121	Software not commercially available
49. Jacobs C, van Rikxoort EM, Twellmann T, et al. Automatic detection of subsolid pulmonary nodules in thoracic computed tomography images. Med Image Anal 2014;18(2):374-84. doi: https://dx.doi.org/10.1016/j.media.2013.12.001	Software not commercially available
50. Kuo C-FJ, Barman J, Hsieh CW, et al. Fast fully automatic detection, classification and 3D reconstruction of pulmonary nodules in CT images by local image feature analysis. Biomedical Signal Processing and Control 2021;68:102790. doi: http://dx.doi.org/10.1016/j.bspc.2021.102790	Software not commercially available
51. Lassen BC, Jacobs C, Kuhnigk JM, et al. Robust semi-automatic segmentation of pulmonary subsolid nodules in chest computed tomography scans. Phys Med Biol 2015;60(3):1307-23. doi: https://dx.doi.org/10.1088/0031-9155/60/3/1307	Software not commercially available
52. Liang F, Li C, Fu X. Evaluation of the Effectiveness of Artificial Intelligence Chest CT Lung Nodule Detection Based on Deep Learning. J 2021;2021:9971325. doi: https://dx.doi.org/10.1155/2021/9971325	Software not commercially available
53. Liang J, Ye G, Guo J, et al. Reducing False-Positives in Lung Nodules Detection Using Balanced Datasets. Front 2021;9:671070. doi: https://dx.doi.org/10.3389/fpubh.2021.671070	Software not commercially available
54. Liu C, Hu SC, Wang C, et al. Automatic detection of pulmonary nodules on CT images with YOLOv3: development and evaluation using simulated and patient data. Quant 2020;10(10):1917-29. doi: https://dx.doi.org/10.21037/qims-19-883	Software not commercially available
55. Liu JB, Liu LH, He W, et al. Computer-aided detection of pulmonary nodules in computed tomography images: Effect on observer performance. Journal of Medical Imaging and Health Informatics 2017;7(6):1205-11. doi: http://dx.doi.org/10.1166/jmihi.2017.2201	Software not commercially available
56. Liu JK, Jiang HY, Gao MD, et al. An Assisted Diagnosis System for Detection of Early Pulmonary Nodule in Computed Tomography Images. J Med Syst 2017;41(2):30. doi: https://dx.doi.org/10.1007/s10916-016-0669-0	Software not commercially available
57. Long C, Hackett T, Yang D, et al. Automatic detection and diagnosis of pulmonary nodule using deep convolutional neural network. Canadian Journal of Respiratory, Critical Care, and Sleep Medicine 2019;3(Supplement 1):11. doi: http://dx.doi.org/10.1080/24745332.2019.1623590	Software not commercially available
58. Masood A, Yang P, Sheng B, et al. Cloud-Based Automated Clinical Decision Support System for Detection and Diagnosis of Lung Cancer in Chest CT. IEEE J Transl Eng Health Med 2020;8:4300113. doi: https://dx.doi.org/10.1109/JTEHM.2019.2955458	Software not commercially available
59. Nguyen CC, Tran GS, Nguyen VT, et al. Pulmonary Nodule Detection Based on Faster R-CNN With Adaptive Anchor Box. IEEE Access 2021;9:154740-51. doi: 10.1109/ACCESS.2021.3128942	Software not commercially available
60. Nomura Y, Higaki T, Fujita M, et al. Effects of Iterative Reconstruction Algorithms on Computer-assisted Detection (CAD) Software for Lung Nodules in Ultra-low-dose CT for Lung Cancer Screening. Acad Radiol 2017;24(2):124-30. doi: https://dx.doi.org/10.1016/j.acra.2016.09.023	Software not commercially available

Reference	Main reason for exclusion
61. Paing MP, Hamamoto K, Tungjitkusolmun S, et al. Automatic Detection and Staging of Lung Tumors using Locational Features and Double-Staged Classifications. Appl Sci-Basel 2019;9(11) doi: 10.3390/app9112329	Software not commercially available
62. Pereira FR, De Andrade JMC, Escuissato DL, et al. Classifier Ensemble Based on Computed Tomography Attenuation Patterns for Computer-Aided Detection System. IEEE Access 2021;9:123134-45. doi: 10.1109/ACCESS.2021.3109860	Software not commercially available
63. Qiu Z, Wu Q, Wang S, et al. Development of a deep learning-based method to diagnose pulmonary ground glass nodules by sequential computed tomography imaging. Thorac Cancer 2022;06:06. doi: https://dx.doi.org/10.1111/1759-7714.14305	Software not commercially available
64. Savic M, Ma Y, Ramponi G, et al. Lung Nodule Segmentation with a Region-Based Fast Marching Method. Sensors (Basel) 2021;21(5):09. doi: https://dx.doi.org/10.3390/s21051908	Software not commercially available
65. Seito AAA, Jacobs C, Ciompi F, et al. Computer-Aided Detection of Lung Cancer: Combining Pulmonary Nodule Detection Systems with a Tumor Risk Prediction Model. Medical Imaging 2015: Computer-Aided Diagnosis 2015;9414 doi: 10.1117/12.2080955	Software not commercially available
66. Silva M, Capretti G, Sverzellati N, et al. Non-solid and part-solid nodules: Comparison between visual and computer aided detection. J Thorac Imaging 2017;32(4):W19. doi: http://dx.doi.org/10.1097/RTI.0000000000000288	Software not commercially available
67. Silva M, Schaefer-Prokop CM, Jacobs C, et al. Detection of Subsolid Nodules in Lung Cancer Screening: Complementary Sensitivity of Visual Reading and Computer-Aided Diagnosis. Invest Radiol 2018;53(8):441-49. doi: https://dx.doi.org/10.1097/RLI.0000000000000464	Software not commercially available
68. Song J, Huang SC, Kelly B, et al. Automatic lung nodule segmentation and intra-nodular heterogeneity image generation. IEEE j 2021;15:15. doi: https://dx.doi.org/10.1109/JBHI.2021.3135647	Software not commercially available
69. Tammemagi M, Ritchie AJ, Atkar-Khattra S, et al. Predicting Malignancy Risk of Screen-Detected Lung Nodules-Mean Diameter or Volume. J Thorac Oncol 2019;14(2):203-11. doi: https://dx.doi.org/10.1016/j.jtho.2018.10.006	Software not commercially available
70. Tan JR, Cheong EHT, Chan LP, et al. Implementation of an Artificial Intelligence-Based Double Read System in Capturing Pulmonary Nodule Discrepancy in CT Studies. Curr Probl Diagn Radiol 2021;50(2):119-22. doi: https://dx.doi.org/10.1067/j.cpradiol.2020.07.006	Software not commercially available
71. Terasawa T, Aoki T, Murakami S, et al. Detection of lung carcinoma with predominant ground glass opacity on CT using temporal subtraction method. Eur Radiol 2018;28(4):1594-99. doi: https://dx.doi.org/10.1007/s00330-017-5085-4	Software not commercially available
72. Wang YQ, Yue SH, Li Q, et al. Research on Technologies of Computer Aided Diagnosis for Solitary Pulmonary Nodule Based on CT Images. 2019 Ieee International Instrumentation and Measurement Technology Conference (I2mtc) 2019:724-28.	Software not commercially available
73. Woo M, Devane AM, Lowe SC, et al. Deep learning for semi-automated unidirectional measurement of lung tumor size in CT. Cancer Imaging 2021;21(1):43. doi: https://dx.doi.org/10.1186/s40644-021-00413-7	Software not commercially available
74. Xu YM, Zhang T, Xu H, et al. Deep Learning in CT Images: Automated Pulmonary Nodule Detection for Subsequent Management Using Convolutional Neural Network. Cancer Manag Res 2020;12:2979-92. doi: https://dx.doi.org/10.2147/CMAR.S239927	Software not commercially available
75. Yen A, Pfeffer Y, Blumenfeld A, et al. Use of a Dual Artificial Intelligence Platform to Detect Unreported Lung Nodules. J Comput Assist Tomogr 2021;45(2):318-22. doi: https://dx.doi.org/10.1097/RCT.0000000000001118	Software not commercially available
76. Young S, Lo P, Kim G, et al. The effect of radiation dose reduction on computer-aided detection (CAD) performance in a low-dose lung cancer screening population. Med Phys 2017;44(4):1337-46. doi: https://dx.doi.org/10.1002/mp.12128	Software not commercially available
77. Yu H, Li J, Zhang L, et al. Design of lung nodules segmentation and recognition algorithm based on deep learning. BMC Bioinformatics 2021;22(Suppl 5):314. doi: https://dx.doi.org/10.1186/s12859-021-04234-0	Software not commercially available

Reference	Main reason for exclusion
78. Zhang QH, Kong XJ. Design of Automatic Lung Nodule Detection System Based on Multi-Scene Deep Learning Framework. IEEE Access 2020;8:90380-89. doi: 10.1109/ACCESS.2020.2993872	Software not commercially available
79. Zuo W, Zhou F, He Y. An Embedded Multi-branch 3D Convolution Neural Network for False Positive Reduction in Lung Nodule Detection. J Digit Imaging 2020;33(4):846-57. doi: https://dx.doi.org/10.1007/s10278-020-00326-0	Software not commercially available
**Excluded on technology: No appropriate regulatory approval (CE mark) across the UK and the EU (n=32)**	
80. Benzakoun J, Bommart S, Coste J, et al. Computer-aided diagnosis (CAD) of subsolid nodules: Evaluation of a commercial CAD system. Eur J Radiol 2016;85(10):1728-34. doi: https://dx.doi.org/10.1016/j.ejrad.2016.07.011	No CE mark
81. Brown M, Browning P, Wahi-Anwar MW, et al. Integration of Chest CT CAD into the Clinical Workflow and Impact on Radiologist Efficiency. Acad Radiol 2019;26(5):626-31. doi: https://dx.doi.org/10.1016/j.acra.2018.07.006	No CE mark
82. Buls N, Watte N, Nieboer K, et al. Performance of an artificial intelligence tool with real-time clinical workflow integration - Detection of intracranial hemorrhage and pulmonary embolism. Phys Med 2021;83:154-60. doi: http://dx.doi.org/10.1016/j.ejmp.2021.03.015	No CE mark
83. Chen K, Lai YC, Vanniarajan B, et al. Clinical impact of a deep learning system for automated detection of missed pulmonary nodules on routine body computed tomography including the chest region. Eur Radiol 2022;09:09. doi: https://dx.doi.org/10.1007/s00330-021-08412-9	No CE mark
84. Chen L, Gu D, Chen Y, et al. An artificial-intelligence lung imaging analysis system (ALIAS) for population-based nodule computing in CT scans. Comput Med Imaging Graph 2021;89:101899. doi: https://dx.doi.org/10.1016/j.compmedimag.2021.101899	No CE mark
85. Cho J, Kim J, Lee KJ, et al. Incidence Lung Cancer after a Negative CT Screening in the National Lung Screening Trial: Deep Learning-Based Detection of Missed Lung Cancers. J 2020;9(12):02. doi: https://dx.doi.org/10.3390/jcm9123908	No CE mark
86. Ctri. To Study how accurately of Predible Lung detects Lung Nodules. https://trialsearchwhoint/Trial2aspx?TrialID=CTRI/2019/07/020120 2019	No CE mark
87. Cui X, Ye Z, Zheng S, et al. Validation of a deep learning-based computer-aided system for lung nodule detection in a Chinese lung cancer screening program. Eur Respir J 2020;56(Supplement 64) doi: https://dx.doi.org/10.1183/13993003.congress-2020.4168	No CE mark
88. Cui X, Ye Z, Zheng S, et al. P42.02 Evaluating the Feasibility of a Deep Learning-Based Computer-Aided Detection System for Lung Nodule Detection in a Lung Cancer Screening Program. Journal of Thoracic Oncology 2021;16(3 Supplement):S477-S78. doi: https://dx.doi.org/10.1016/j.jtho.2021.01.826	No CE mark
89. Den Harder AM, Willemink MJ, van Hamersvelt RW, et al. Effect of radiation dose reduction and iterative reconstruction on computer-aided detection of pulmonary nodules: Intra-individual comparison. Eur J Radiol 2016;85(2):346-51. doi: https://dx.doi.org/10.1016/j.ejrad.2015.12.003	No CE mark
90. Guo X, Li Y, Yang C, et al. Deep Learning-Based Computed Tomography Imaging to Diagnose the Lung Nodule and Treatment Effect of Radiofrequency Ablation. J 2021;2021:6556266. doi: https://dx.doi.org/10.1155/2021/6556266	No CE mark
91. Han D, Heuvelmans M, Rook M, et al. Evaluation of a novel deep learning-based classifier for perifissural nodules. Eur Radiol 2021;31(6):4023-30. doi: https://dx.doi.org/10.1007/s00330-020-07509-x	No CE mark
92. Jacobs C, van Ginneken B. Google's lung cancer AI: a promising tool that needs further validation. Nat Rev Clin Oncol 2019;16(9):532-33. doi: https://dx.doi.org/10.1038/s41571-019-0248-7	No CE mark
93. Jeon KN, Goo JM, Lee CH, et al. Computer-aided nodule detection and volumetry to reduce variability between radiologists in the interpretation of lung nodules at low-dose screening computed tomography. Invest Radiol 2012;47(8):457-61. doi: https://dx.doi.org/10.1097/RLI.0b013e318250a5aa	No CE mark

Reference	Main reason for exclusion
94. Jprn U. Japanese Multi-Center Study for Utility of 3D Computer-Aided Detection System at Lung Cancer Screening with Low-dose CT Protocol. https://trialsearchwhoint/Trial2aspx?TrialID=JPRN-UMIN000030415 2018	No CE mark
95. Jurkovic IA, Papanikolaou N, Stathakis S, et al. Objective assessment of the quality and accuracy of deformable image registration. J 2020;45(3):156-67. doi: https://dx.doi.org/10.4103/jmp.JMP_47_19	No CE mark
96. Li L, Liu Z, Huang H, et al. Evaluating the performance of a deep learning-based computer-aided diagnosis (DL-CAD) system for detecting and characterizing lung nodules: Comparison with the performance of double reading by radiologists. Thorac Cancer 2019;10(2):183-92. doi: https://dx.doi.org/10.1111/1759-7714.12931	No CE mark
97. Li X, Guo F, Zhou Z, et al. Performance of Deep-learning-based Artificial Intelligence on Detection of Pulmonary Nodules in Chest CT. Zhongguo fei ai za zhi [Chinese journal of lung cancer] 2019;22(6):336-40. doi: 10.3779/j.issn.1009-3419.2019.06.02	No CE mark
98. Liu Z, Li L, Li T, et al. Does a Deep Learning-Based Computer-Assisted Diagnosis System Outperform Conventional Double Reading by Radiologists in Distinguishing Benign and Malignant Lung Nodules? Front 2020;10:545862. doi: http://dx.doi.org/10.3389/fonc.2020.545862	No CE mark
99. Matsumoto S, Ohno Y, Aoki T, et al. Computer-aided detection of lung nodules on multidetector CT in concurrent-reader and second-reader modes: a comparative study. Eur J Radiol 2013;82(8):1332-7. doi: https://dx.doi.org/10.1016/j.ejrad.2013.02.005	No CE mark
100. Ohno Y, Aoyagi K, Chen Q, et al. Comparison of computer-aided detection (CADe) capability for pulmonary nodules among standard-, reduced- and ultra-low-dose CTs with and without hybrid type iterative reconstruction technique. Eur J Radiol 2018;100:49-57. doi: https://dx.doi.org/10.1016/j.ejrad.2018.01.010	No CE mark
101. Ohno Y, Aoyagi K, Takenaka D, et al. Machine learning for lung CT texture analysis: Improvement of inter-observer agreement for radiological finding classification in patients with pulmonary diseases. Eur J Radiol 2021;134:109410. doi: https://dx.doi.org/10.1016/j.ejrad.2020.109410	No CE mark
102. Ohno Y, Aoyagi K, Yaguchi A, et al. 3D CADv system with and without CNN: Comparison of nodule component measurement accuracy and differentiation in routine clinical practice data. International Journal of Computer Assisted Radiology and Surgery 2020;15(1 Supplement):S114-S15. doi: http://dx.doi.org/10.1007/s11548-020-02171-6	No CE mark
103. Ohno Y, Aoyagi K, Yaguchi A, et al. Differentiation of Benign from Malignant Pulmonary Nodules by Using a Convolutional Neural Network to Determine Volume Change at Chest CT. Radiology 2020;296(2):432-43. doi: https://dx.doi.org/10.1148/radiol.2020191740	No CE mark
104. Ohri B, Smith D, Melville P, et al. Use of "artificial Intelligence" to Aid Pulmonary Nodule Assessment. Respirology 2020;25:177. doi: https://dx.doi.org/10.1111/resp.13778	No CE mark
105. Prakashini K, Babu S, Rajgopal KV, et al. Role of Computer Aided Diagnosis (CAD) in the detection of pulmonary nodules on 64 row multi detector computed tomography. Lung India 2016;33(4):391-7. doi: https://dx.doi.org/10.4103/0970-2113.184872	No CE mark
106. Qi LL, Wu BT, Tang W, et al. Long-term follow-up of persistent pulmonary pure ground glass nodules with deep learning-assisted nodule segmentation. Eur Radiol 2020;30(2):744-55. doi: https://dx.doi.org/10.1007/s00330-019-06344-z	No CE mark
107. Wang YW, Wang JW, Yang SX, et al. Proposing a deep learning-based method for improving the diagnostic certainty of pulmonary nodules in CT scan of chest. Eur Radiol 2021;31(11):8160-67. doi: https://dx.doi.org/10.1007/s00330-021-07919-5	No CE mark
108. Wu N, Li X, Luo X. P62.10 AI-Based Three-Dimension Reconstruction for Pulmonary Nodules -New Auxiliary Exploration for Thoracic Surgery. Journal of Thoracic Oncology 2021;16(10 Supplement):S1181. doi: http://dx.doi.org/10.1016/j.jtho.2021.08.655	No CE mark
109. Yanagawa M, Honda O, Kikuyama A, et al. Pulmonary nodules: effect of adaptive statistical iterative reconstruction (ASIR) technique on performance of a computer-aided detection (CAD) system-comparison of performance between different-dose CT scans. Eur J Radiol 2012;81(10):2877-86. doi: https://dx.doi.org/10.1016/j.ejrad.2011.09.011	No CE mark

Reference	Main reason for exclusion
110. Yang D, Bai C, Hu J, et al. Deep convolutional neutral networks based artificial intelligence system for pulmonary nodule detection and diagnosis in United States and Chinese dataset. American Journal of Respiratory and Critical Care Medicine 2018;197(MeetingAbstracts)	No CE mark
111. Yuan R, Mayo J, Streit I, et al. MA10.06 Randomized Clinical Trial with Computer Assisted Diagnosis (CAD) Versus Radiologist as First Reader of Lung Screening LDCT. Journal of Thoracic Oncology 2019;14(10 Supplement):S287-S88. doi: http://dx.doi.org/10.1016/j.jtho.2019.08.578	No CE mark
**Excluded on technology: Manufacturer eligible but other software (n=13)**	
112. Azour L, Moore WH, O'Donnell T, et al. Inter-Reader Variability of Volumetric Subsolid Pulmonary Nodule Radiomic Features. Acad Radiol 2021;17:17. doi: https://dx.doi.org/10.1016/j.acra.2021.01.026	Manufacturer eligible but other software
113. Bogoni L, Ko JP, Alpert J, et al. Impact of a computer-aided detection (CAD) system integrated into a picture archiving and communication system (PACS) on reader sensitivity and efficiency for the detection of lung nodules in thoracic CT exams. J Digit Imaging 2012;25(6):771-81. doi: https://dx.doi.org/10.1007/s10278-012-9496-0	Manufacturer eligible but other software
114. Godoy MC, Kim TJ, White CS, et al. Benefit of computer-aided detection analysis for the detection of subsolid and solid lung nodules on thin- and thick-section CT. AJR Am J Roentgenol 2013;200(1):74-83. doi: https://dx.doi.org/10.2214/AJR.11.7532	Manufacturer eligible but other software
115. Heuvelmans M, Oudkerk M, Zhao YR, et al. Comparison of three software systems for semi-automatic volumetry of pulmonary nodules on baseline and follow-up CT examinations. Acta Radiol 2014;55(6):691-98. doi: http://dx.doi.org/10.1177/0284185113508177	Manufacturer eligible but other software
116. Jacobs C, van Rikxoort EM, Murphy K, et al. Computer-aided detection of pulmonary nodules: a comparative study using the public LIDC/IDRI database. Eur Radiol 2016;26(7):2139-47. doi: https://dx.doi.org/10.1007/s00330-015-4030-7	Manufacturer eligible but other software
117. Larici AR, Amato M, Ordonez P, et al. Detection of noncalcified pulmonary nodules on low-dose MDCT: comparison of the sensitivity of two CAD systems by using a double reference standard. Radiol Med (Torino) 2012;117(6):953-67.	Manufacturer eligible but other software
118. Liang M, Tang W, Xu DM, et al. Low-Dose CT Screening for Lung Cancer: Computer-aided Detection of Missed Lung Cancers. Radiology 2016;281(1):279-88. doi: https://dx.doi.org/10.1148/radiol.2016150063	Manufacturer eligible but other software
119. Messerli M, Kluckert T, Knitel M, et al. Computer-aided detection (CAD) of solid pulmonary nodules in chest x-ray equivalent ultralow dose chest CT - first in-vivo results at dose levels of 0.13mSv. Eur J Radiol 2016;85(12):2217-24. doi: https://dx.doi.org/10.1016/j.ejrad.2016.10.006	Manufacturer eligible but other software
120. Mozaffary A, Trabzonlu TA, Lombardi P, et al. Integration of fully automated computer-aided pulmonary nodule detection into CT pulmonary angiography studies in the emergency department: effect on workflow and diagnostic accuracy. Emerg 2019;26(6):609-14. doi: https://dx.doi.org/10.1007/s10140-019-01707-x	Manufacturer eligible but other software
121. Nair A, Gartland N, Barton B, et al. Comparing the performance of trained radiographers against experienced radiologists in the UK lung cancer screening (UKLS) trial. Br J Radiol 2016;89(1066):20160301. doi: https://dx.doi.org/10.1259/bjr.20160301	Manufacturer eligible but other software
122. Nair A, Screaton NJ, Holemans JA, et al. The impact of trained radiographers as concurrent readers on performance and reading time of experienced radiologists in the UK Lung Cancer Screening (UKLS) trial. Eur Radiol 2018;28(1):226-34. doi: https://dx.doi.org/10.1007/s00330-017-4903-z	Manufacturer eligible but other software
123. Takahashi EA, Koo CW, White DB, et al. Prospective Pilot Evaluation of Radiologists and Computer-aided Pulmonary Nodule Detection on Ultra-low-Dose CT With Tin Filtration. J Thorac Imaging 2018;33(6):396-401. doi: https://dx.doi.org/10.1097/RTI.0000000000000348	Manufacturer eligible but other software

Reference		Main reason for exclusion
124.	Zhao Y, de Bock GH, Vliegenthart R, et al. Performance of computer-aided detection of pulmonary nodules in low-dose CT: comparison with double reading by nodule volume. Eur Radiol 2012;22(10):2076-84. doi: https://dx.doi.org/10.1007/s00330-012-2437-y	Manufacturer eligible but other software
**Excluded on technology: Software name unclear, no author reply (n=15)**		
125.	Arteta C, Novotny P, Santos C, et al. Automatic Nodule Size Measurements Can Improve Prediction Accuracy Within a Brock Risk Model. Journal of Thoracic Oncology 2018;13(10 Supplement):S429. doi: http://dx.doi.org/10.1016/j.jtho.2018.08.490	Software name unclear; no author reply received
126.	Brown MS, Kim HJ, Lo P, et al. Automated tumor size assessment: Consistency of computer measurements with an expert panel. Journal of Clinical Oncology 2013;31(15 SUPPL. 1)	Software name unclear; no author reply received
127.	Brown MS, Lo P, Barnoy E, et al. Clinically usable computer-aided detection (CAD) system for lung cancer screening with CT. American Journal of Respiratory and Critical Care Medicine 2013;187(MeetingAbstracts)	Software name unclear; no author reply received
128.	Gu X, Xie W, Fang Q, et al. The effect of pulmonary vessel suppression on computerized detection of nodules in chest CT scans. Med Phys 2020;47(10):4917-27. doi: https://dx.doi.org/10.1002/mp.14401	Software name unclear; no author reply received
129.	Gu XM, Chai YL, Weiyang X, et al. Effect of CAD system with a vessel suppression function on clinical lung nodule detection in chest CT scans. Medical Imaging 2021: Image Perception, Observer Performance, and Technology Assessment 2021;11599 doi: 10.1117/12.2582059	Software name unclear; no author reply received
130.	Gu XM, Xie WY, Fang QM, et al. Lung vessel suppression and its effect on nodule detection in chest CT scans. Medical Imaging 2020: Computer-Aided Diagnosis 2020;11314 doi: 10.1117/12.2549405	Software name unclear; no author reply received
131.	Lieman-Sifry J, Brouha S, Weihe E, et al. Deep learning-based cad may improve detection of pulmonary nodules while preserving a low false-positive rate. J Thorac Imaging 2019;34(4):W61. doi: http://dx.doi.org/10.1097/RTI.0000000000000421	Software name unclear; no author reply received
132.	Liu Y, Luo H, Qing H, et al. Screening baseline characteristics of early lung cancer on low-dose computed tomography with computer-aided detection in a Chinese population. Cancer epidemiol 2019;62:101567. doi: https://dx.doi.org/10.1016/j.canep.2019.101567	Software name unclear; no author reply received
133.	Miki S, Nomura Y, Hayashi N, et al. Prospective Study of Spatial Distribution of Missed Lung Nodules by Readers in CT Lung Screening Using Computer-assisted Detection. Acad Radiol 2021;28(5):647-54. doi: https://dx.doi.org/10.1016/j.acra.2020.03.015	Software name unclear; no author reply received
134.	Ohno Y, Seki S, Yoshikawa T, et al. Convolutional Neural Network for 3D CADv Systems: Utility for Differentiation of Malignant from Benign Pulmonary Nodules. International Journal of Computer Assisted Radiology and Surgery 2019;14(Supplement 1):S67. doi: http://dx.doi.org/10.1007/s11548-019-01969-3	Software name unclear; no author reply received
135.	Setio AA, Jacobs C, Gelderblom J, et al. Automatic detection of large pulmonary solid nodules in thoracic CT images. Med Phys 2015;42(10):5642-53. doi: https://dx.doi.org/10.1118/1.4929562	Software name unclear; no author reply received
136.	Tokunaga S, Hazeki N, Tamura D, et al. Computer-aided detection (CAD) as concurrent vs. second reader for lung nodules on CT in a Japanese multicenter study: Evaluation of reading time and observer performance in radiologists and pulmonologists. Chest 2013;144(4 MEETING ABSTRACT) doi: http://dx.doi.org/10.1378/chest.1703410	Software name unclear; no author reply received
137.	Werner S, Gast R, Horger M, et al. Accuracy and Reproducibility of a Software Prototype for Semi-Automated Computer-Aided Volumetry of the solid and subsolid Components of part-solid Pulmonary Nodules. RoFo Fortschritte auf dem Gebiet der Rontgenstrahlen und der Bildgebenden Verfahren 2021 doi: http://dx.doi.org/10.1055/a-1656-9834	Software name unclear; no author reply received
138.	Zheng S, Cui X, Vonder M, et al. Deep learning-based pulmonary nodule detection: Effect of slab thickness in maximum intensity projections at the nodule candidate detection stage. Comput Methods Programs Biomed 2020;196:105620. doi: https://dx.doi.org/10.1016/j.cmpb.2020.105620	Software name unclear; no author reply received

Reference		Main reason for exclusion
139.	Zheng S, Cui X, Ye Z, et al. P42.06 Automatic Lung Nodule Detection by a Deep Learning-Based CAD System: The Value of Slab Thickness in the Maximum Intensity Projection Technique. Journal of Thoracic Oncology 2021;16(3 Supplement):S479-S80. doi: https://dx.doi.org/10.1016/j.jtho.2021.01.830	Software name unclear; no author reply received
**Excluded on outcomes: Clinical trial register, no outcomes yet (n=4)**		
140.	fdeeeKCT0005065. A multi-center, retrospective pivotal trial to evaluate the efficacy of artificial intelligence-based pulmonary nodule detection software ‘VUNO Med – Lung CAD’ in thoracic CT: http://cris.nih.go.kr/cris/en/search/search_result_st01.jsp?seq=16420, 2020.	Clinical trial register, no outcomes yet
141.	Institute VP, University S, Technologies R. Evaluation of Computer-Aided Lung Nodule Detection Software in Thoracic CT for Riverain Technologies LLC: https://ClinicalTrials.gov/show/NCT02440139, 2015.	Clinical trial register, no outcomes yet
142.	NCT04119960. Clinical Validation of InferRead Lung CT.AI: https://clinicaltrials.gov/show/NCT04119960, 2019.	Clinical trial register, no outcomes yet
143.	NCT04792632. Clinical Performance Evaluation of Veye Lung Nodules: https://clinicaltrials.gov/show/NCT04792632, 2021.	Clinical trial register, no outcomes yet
**Excluded on outcomes: No relevant test accuracy outcomes reported (n=13)**		
144.	Buckler AJ, Danagoulian J, Johnson K, et al. Inter-Method Performance Study of Tumor Volumetry Assessment on Computed Tomography Test-Retest Data. Acad Radiol 2015;22(11):1393-408. doi: https://dx.doi.org/10.1016/j.acra.2015.08.007	No relevant test accuracy outcomes
145.	ChiCTR1900021144. Evaluation of AI-assisted detection of lung nodules in low dose CT images: http://www.chictr.org.cn/showproj.aspx?proj=35698, 2019.	No relevant test accuracy outcomes
146.	ChiCTR2000029278. A blinded, self-control trial to evaluate an AI based CAD system for Lung Nodule Diagnosis: http://www.chictr.org.cn/showproj.aspx?proj=48219, 2020.	No relevant test accuracy outcomes
147.	Ganti S. Radiological lessons, tips and tricks from UK's first lung cancer screening site. Lung Cancer 2020;139(Supplement 1):S6. doi: http://dx.doi.org/10.1016/S0169-5002%2820%2930041-6	No relevant test accuracy outcomes
148.	Heuvelmans MA, Walter JE, Vliegenthart R, et al. Disagreement of diameter and volume measurements for pulmonary nodule size estimation in CT lung cancer screening. Thorax 2018;73(8):779-81. doi: https://dx.doi.org/10.1136/thoraxjnl-2017-210770	No relevant test accuracy outcomes
149.	Hwang EJ, Goo JM, Kim HY, et al. Variability in interpretation of low-dose chest CT using computerized assessment in a nationwide lung cancer screening program: comparison of prospective reading at individual institutions and retrospective central reading. Eur Radiol 2021;31(5):2845-55. doi: https://dx.doi.org/10.1007/s00330-020-07424-1	No relevant test accuracy outcomes
150.	Kisby G, Dentry M. Use of computer-aided detection (CAD) in CT Chest imaging for the diagnosis of lung nodules. Journal of Medical Imaging and Radiation Oncology 2021;65(SUPPL 1):143. doi: http://dx.doi.org/10.1111/1754-9485.13301	No relevant test accuracy outcomes
151.	Lee J, Kim Y, Kim HY, et al. Feasibility of implementing a national lung cancer screening program: Interim results from the Korean Lung Cancer Screening Project (K-LUCAS). Transl 2021;10(2):723-36. doi: https://dx.doi.org/10.21037/tlcr-20-700	No relevant test accuracy outcomes
152.	Lee J, Lim J, Kim Y, et al. Development of Protocol for Korean Lung Cancer Screening Project (K-LUCAS) to Evaluate Effectiveness and Feasibility to Implement National Cancer Screening Program. Cancer Res 2019;51(4):1285-94. doi: https://dx.doi.org/10.4143/crt.2018.464	No relevant test accuracy outcomes
153.	Milanese G, Eberhard M, Martini K, et al. Vessel suppressed chest Computed Tomography for semi-automated volumetric measurements of solid pulmonary nodules. Eur J Radiol 2018;101:97-102. doi: https://dx.doi.org/10.1016/j.ejrad.2018.02.020	No relevant test accuracy outcomes

Reference		Main reason for exclusion
154.	Nct. Evaluation of Use of Diagnostic AI for Lung Cancer in Practice. https://clinicaltrialsgov/show/NCT03780582 2018	No relevant test accuracy outcomes
155.	Park S, Lee SM, Do KH, et al. Deep Learning Algorithm for Reducing CT Slice Thickness: Effect on Reproducibility of Radiomic Features in Lung Cancer. Korean J Radiol 2019;20(10):1431-40. doi: https://dx.doi.org/10.3348/kjr.2019.0212	No relevant test accuracy outcomes
156.	Schreuder A, van Ginneken B, Scholten ET, et al. Classification of CT Pulmonary Opacities as Perifissural Nodules: Reader Variability. Radiology 2018;288(3):867-75. doi: https://dx.doi.org/10.1148/radiol.2018172771	No relevant test accuracy outcomes
**Excluded on publication type: Conference abstract with no additional data to an included full-text paper reported (n=3)**		
157.	Hall H, Ruparel M, Horst C, et al. The role of computer-assisted radiographer reporting in lung cancer screening programmes. Thorax 2019;74(Supplement 2):A131-A32. doi: http://dx.doi.org/10.1136/thorax-2019-BTSabstracts2019.221	Conference abstract with no additional data reported
158.	Hwang EJ, Yoon SH, Goo JM, et al. P2.11-16 Variability in Reading Low-Dose Chest CT: Individual Readers vs. Central Review in a Nationwide Lung Cancer Screening Project. Journal of Thoracic Oncology 2019;14(10 Supplement):S798-S99. doi: http://dx.doi.org/10.1016/j.jtho.2019.08.1716	Conference abstract with no additional data reported
159.	Lo S, Freedman M, Mun SK. The application of a vessel suppressed function incorporated with lung opacity analysis for the significant increase of nodule detectability in CT. International Journal of Computer Assisted Radiology and Surgery 2017;12(1 Supplement 1):S150. doi: http://dx.doi.org/10.1007/s11548-017-1588-3	Conference abstract with no additional data reported
**Excluded on publication type: No primary research article (n=2)**		
160.	Crosby D, Lyons N, Greenwood E, et al. A roadmap for the early detection and diagnosis of cancer. The Lancet Oncology 2020;21(11):1397-99. doi: http://dx.doi.org/10.1016/S1470-2045%2820%2930593-3	No primary research article
161.	Svoboda E. Artificial intelligence is improving the detection of lung cancer. Nature 2020;587(7834):S20-S22. doi: https://dx.doi.org/10.1038/d41586-020-03157-9	No primary research article
**Excluded: Duplicate (n=2)**		
162.	Mun SK, Lo SB, Freedman MT, et al. Computer-aided detection of lung nodules on CT with a computerized pulmonary vessel suppressed function. American Journal of Roentgenology 2018;210(3):480-88. doi: http://dx.doi.org/10.2214/AJR.17.18718	Duplicate
163.	Yuan R, Mayo J, Streit I, et al. Randomized Clinical Trial with Computer Assisted Diagnosis (CAD) Versus Radiologist as First Reader of Lung Screening LDCT. Journal of Thoracic Oncology 2019;14(10):S287-S88. doi: 10.1016/j.jtho.2019.08.578	Duplicate

B. Publications excluded after review of full-text articles – Updated electronic database searches (n=122)

Reference	Main reason for exclusion
**Excluded on population: <90% lung cancer screening LDCT (n=16)**	
1. Alshaibani NN, Alkhatlan NM, Alanazi NA, et al. DETECTION AND DIAGNOSIS OF LUNG CANCER USING CNN BASED ARTIFICIAL INTELLIGENCE. Journal of Pharmaceutical Negative Results 2022;13:4121-34. doi: https://dx.doi.org/10.47750/pnr.2022.13.S07.517	<90% lung cancer screening LDCT
2. Cavallo JJ, de Oliveira Santo I, Mezrich JL, et al. Clinical Implementation of a Combined Artificial Intelligence and Natural Language Processing Quality Assurance Program for Pulmonary Nodule Detection in the Emergency Department Setting. J 2023;02:02. doi: https://dx.doi.org/10.1016/j.jacr.2022.12.016	<90% lung cancer screening LDCT
3. Chen X, Qi Q, Sun Z, et al. Total nodule number as an independent prognostic factor in resected stage III non-small cell lung cancer: a deep learning-powered study. Ann 2022;10(2):33. doi: https://dx.doi.org/10.21037/atm-21-3231	<90% lung cancer screening LDCT
4. Chen X, Xu H, Qi Q, et al. AI-based chest CT semantic segmentation algorithm enables semi-automated lung cancer surgery planning by recognizing anatomical variants of pulmonary vessels. Front 2022;12:1021084. doi: https://dx.doi.org/10.3389/fonc.2022.1021084	<90% lung cancer screening LDCT
5. Chen Y, Tian X, Fan K, et al. The Value of Artificial Intelligence Film Reading System Based on Deep Learning in the Diagnosis of Non-Small-Cell Lung Cancer and the Significance of Efficacy Monitoring: A Retrospective, Clinical, Nonrandomized, Controlled Study. Comput 2022;2022:2864170. doi: https://dx.doi.org/10.1155/2022/2864170	<90% lung cancer screening LDCT
6. De Lucia F, Amer Ouali R, Devriendt A, et al. Comparison of Chest Computed Tomography Between the Two Waves of Coronavirus Disease 2019 in Belgium Using Artificial Intelligence. Cureus 2022;14(2):e22203. doi: https://dx.doi.org/10.7759/cureus.22203	<90% lung cancer screening LDCT
7. Hempel HL, Engbersen MP, Wakkie J, et al. Higher agreement between readers with deep learning CAD software for reporting pulmonary nodules on CT. Eur J Radiol Open 2022;9:100435. doi: https://dx.doi.org/10.1016/j.ejro.2022.100435	<90% lung cancer screening LDCT
8. Hu Q, Chen C, Kang S, et al. Application of computer-aided detection (CAD) software to automatically detect nodules under SDCT and LDCT scans with different parameters. Comput Biol Med 2022;146:105538. doi: https://dx.doi.org/10.1016/j.compbiomed.2022.105538	<90% lung cancer screening LDCT
9. Hu Q, Wang S, Chen C, et al. Comparison of two reader modes of computer-aided diagnosis in lung nodules on low-dose chest CT scan. J Innov Opt Health Sci 2022;15(2):2250013. doi: https://dx.doi.org/10.1142/S1793545822500134	<90% lung cancer screening LDCT
10. Kawaguchi Y, Shimada Y, Murakami K, et al. Prognostic impact of artificial intelligence-based volumetric quantification of the solid part of the tumor in clinical stage 0-I adenocarcinoma. Lung Cancer 2022;170:85-90. doi: https://dx.doi.org/10.1016/j.lungcan.2022.06.007	<90% lung cancer screening LDCT
11. Murchison JT, Ritchie G, Senyszak D, et al. Validation of a deep learning computer aided system for CT based lung nodule detection, classification, and growth rate estimation in a routine clinical population. PLoS ONE 2022;17(5):e0266799. doi: https://dx.doi.org/10.1371/journal.pone.0266799	<90% lung cancer screening LDCT
12. Musetescu AE, Gherghina FL, Florescu LM, et al. Computer-Aided Diagnosis of Pulmonary Nodules in Rheumatoid Arthritis. Life (Basel) 2022;12(11):20. doi: https://dx.doi.org/10.3390/life12111935	<90% lung cancer screening LDCT
13. Otsuka Y, Imashimizu K, Hata K, et al. AI-VDT can Help in Detecting Primary Lung Cancer. medRxiv 2022 doi: https://dx.doi.org/10.1101/2022.04.26.22274299	<90% lung cancer screening LDCT
14. Salihoglu YS, Erdemir RU, Puren BA, et al. Diagnostic Performance of Machine Learning Models Based on 18 F-FDG PET/CT Radiomic Features in the Classification of Solitary Pulmonary Nodules. Molecular Imaging and Radionuclide Therapy 2022;31(2):82-88. doi: https://dx.doi.org/10.4274/mirt.galenos.2021.43760	<90% lung cancer screening LDCT

Reference	Main reason for exclusion
15. X Z, Zhu L, D S, et al. Comparison of single- and dual-energy CT combined with artificial intelligence for the diagnosis of pulmonary nodules. Clin Radiol 2023;78(2):e99-e105. doi: 10.1016/j.crad.2022.09.114	<90% lung cancer screening LDCT
16. Zuo Z, Wang P, Zeng W, et al. Measuring pure ground-glass nodules on computed tomography: assessing agreement between a commercially available deep learning algorithm and radiologists' readings. Acta Radiol 2022:2841851221135406. doi: https://dx.doi.org/10.1177/02841851221135406	<90% lung cancer screening LDCT
**Excluded on population: No CT images (n=2)**	
17. Govindarajan A, Tanamala S, Chattoraj S, et al. Role of an Automated Deep Learning Algorithm for Reliable Screening of Abnormality in Chest Radiographs: A Prospective Multicenter Quality Improvement Study. Diagnostics (Basel) 2022;12(11):2724. doi: https://dx.doi.org/10.3390/diagnostics12112724	Chest radiographs
18. Lemieux ME, Reveles XT, Rebeles J, et al. Detection of early-stage lung cancer in sputum using automated flow cytometry and machine learning. Respir Res 2023;24(1):23. doi: https://dx.doi.org/10.1186/s12931-023-02327-3	Sputum cytology
**Excluded on technology: Malignancy risk prediction (n=4)**	
19. Diao K, Chen Y, Liu Y, et al. Diagnostic study on clinical feasibility of an AI-based diagnostic system as a second reader on mobile CT images: a preliminary result. Ann 2022;10(12):668. doi: https://dx.doi.org/10.21037/atm-22-2157	Malignancy risk prediction
20. Maldonado F, Varghese C, Rajagopalan S, et al. Validation of the BRODERS classifier (Benign versus aggressive nODule Evaluation using Radiomic Stratification), a novel high-resolution computed tomography-based radiomic classifier for indeterminate pulmonary nodules. The European respiratory journal 2020 doi: https://dx.doi.org/10.1183/13993003.02485-2020	Malignancy risk prediction
21. Lung Nodule Classification of CT Images Based on the Deep Learning Algorithms. 5th International Conference on Imaging, Signal Processing and Communications (ICISPC); 2021 Jul 23-25; Kumamoto, JAPAN. Ieee.	Malignancy risk prediction
22. Wu XY, Ding F, Li K, et al. Analysis of the Causes of Solitary Pulmonary Nodule Misdiagnosed as Lung Cancer by Using Artificial Intelligence: A Retrospective Study at a Single Center. Diagnostics (Basel) 2022;12(9):13. doi: https://dx.doi.org/10.3390/diagnostics12092218	Malignancy risk prediction
**Excluded on technology: Software not commercially available (n=53)**	
23. Adams SJ, Mondal P, Penz E, et al. Development and Cost Analysis of a Lung Nodule Management Strategy Combining Artificial Intelligence and Lung Reporting and Data Systems for Baseline Lung Cancer Screening. Journal of the American College of Radiology : JACR 2021 doi: https://dx.doi.org/10.1016/j.jacr.2020.11.014	Software not commercially available
24. Agnes SA, Anitha J, Solomon AA. Two-stage lung nodule detection framework using enhanced UNet and convolutional LSTM networks in CT images. Computers in Biology and Medicine 2022;149:19. doi: 10.1016/j.compbiomed.2022.106059	Software not commercially available
25. Albqoor AN, Alzaatreh MY, Almatari MKI. A Novel Method of Segmentation and Analysis of CT Chest Images for Early Lung Cancer Detection. Biomedical and Pharmacology Journal 2022;15(4):1947-56. doi: https://dx.doi.org/10.13005/bpj/2533	Software not commercially available
26. Bai Y, Li D, Duan Q, et al. Analysis of high-resolution reconstruction of medical images based on deep convolutional neural networks in lung cancer diagnostics. Comput Methods Programs Biomed 2022;217:106592. doi: https://dx.doi.org/10.1016/j.cmpb.2021.106592	Software not commercially available
27. Balachandran S, Ranganathan V. Semantic context-aware attention UNET for lung cancer segmentation and classification. Int J Imaging Syst Technol:15. doi: 10.1002/ima.22837	Software not commercially available
28. Bhaskar N, Ganashree TS. Pulmonary Nodule Classification and Lung Cancer Stage Identification by Deep Learning Approach. NeuroQuantology 2022;20(11):6297-309. doi: https://dx.doi.org/10.14704/NQ.2022.20.11.NQ66626	Software not commercially available

Reference	Main reason for exclusion
29. Bhatt SD, Soni HB. Improving Classification Accuracy of Pulmonary Nodules using Simplified Deep Neural Network. Open Biomedical Engineering Journal 2021;15(Suppl 2):180-89. doi: https://dx.doi.org/10.2174/1874120702115010180	Software not commercially available
30. Chae KJ, Jin GY, Ko SB, et al. Deep Learning for the Classification of Small (≤2 cm) Pulmonary Nodules on CT Imaging: a Preliminary Study. Acad Radiol 2020;27(4):e55-e63. doi: 10.1016/j.acra.2019.05.018	Software not commercially available
31. Chen S. Models of Artificial Intelligence-Assisted Diagnosis of Lung Cancer Pathology Based on Deep Learning Algorithms. J 2022;2022:3972298. doi: https://dx.doi.org/10.1155/2022/3972298	Software not commercially available
32. Chetan MR, Dowson N, Price NW, et al. Developing an understanding of artificial intelligence lung nodule risk prediction using insights from the Brock model. Eur Radiol 2022;32(8):5330-38. doi: https://dx.doi.org/10.1007/s00330-022-08635-4	Software not commercially available
33. Du W, He B, Luo X, et al. Diagnostic Value of Artificial Intelligence Based on CT Image in Benign and Malignant Pulmonary Nodules. J 2022;2022:5818423. doi: https://dx.doi.org/10.1155/2022/5818423	Software not commercially available
34. Eid Alazemi F, Jehangir B, Imran M, et al. An Efficient Model for Lungs Nodule Classification Using Supervised Learning Technique. J 2023;2023:8262741. doi: https://dx.doi.org/10.1155/2023/8262741	Software not commercially available
35. Han Y, Qi H, Wang L, et al. Pulmonary nodules detection assistant platform: An effective computer aided system for early pulmonary nodules detection in physical examination. Comput Methods Programs Biomed 2022;217:106680. doi: https://dx.doi.org/10.1016/j.cmpb.2022.106680	Software not commercially available
36. Huidrom R, Chanu YJ, Singh KM. Neuro-evolutional based computer aided detection system on computed tomography for the early detection of lung cancer. Multimed Tools Appl 2022;81(22):32661-73. doi: 10.1007/s11042-022-12722-5	Software not commercially available
37. Hussain MA, Gogoi L. Performance analyses of five neural network classifiers on nodule classification in lung CT images using WEKA: a comparative study. Phys Eng Sci Med 2022;45(4):1193-204. doi: https://dx.doi.org/10.1007/s13246-022-01187-3	Software not commercially available
38. Karrar A, Mabrouk MS, Wahed MA, et al. Auto diagnostic system for detecting solitary and juxtapleural pulmonary nodules in computed tomography images using machine learning. Neural Comput Appl 2023;35(2):1645-59. doi: 10.1007/s00521-022-07844-8	Software not commercially available
39. Katase S, Ichinose A, Hayashi M, et al. Development and performance evaluation of a deep learning lung nodule detection system. BMC med 2022;22(1):203. doi: https://dx.doi.org/10.1186/s12880-022-00938-8	Software not commercially available
40. Khan A, Tariq I, Khan H, et al. Lung Cancer Nodules Detection via an Adaptive Boosting Algorithm Based on Self-Normalized Multiview Convolutional Neural Network. J 2022;2022:5682451. doi: https://dx.doi.org/10.1155/2022/5682451	Software not commercially available
41. Kim S, Jeong WK, Choi JH, et al. Development of deep learning-assisted overscan decision algorithm in low-dose chest CT: Application to lung cancer screening in Korean National CT accreditation program. PLoS ONE 2022;17(9):e0275531. doi: https://dx.doi.org/10.1371/journal.pone.0275531	Software not commercially available
42. Lan CC, Hsieh MS, Hsiao JK, et al. Deep Learning-based Artificial Intelligence Improves Accuracy of Error-prone Lung Nodules. Int J Med Sci 2022;19(3):490-98. doi: https://dx.doi.org/10.7150/ijms.69400	Software not commercially available
43. Lee J, Park JH, Kim M, et al. Improved Lung Cancer Detection in Ultra Low dose CT with Combined AI-based Nodule Detection and Denoising Techniques. International Workshop on Advanced Imaging Technology (IWAIT); 2022 Jan 04-06; Hong Kong, HONG KONG. Spie-Int Soc Optical Engineering.	Software not commercially available
44. Moragheb MA, Badie A, Noshad A. An Effective Approach for Automated Lung Node Detection using CT Scans. J 2022;12(4):377-86. doi: https://dx.doi.org/10.31661/jbpe.v0i0.2110-1412	Software not commercially available

Reference	Main reason for exclusion
45. Nayani ASK, Swapnasri G, Naresh M. Lung Cancer Recognition Using CT Scan with CNN-VGG19 and PNN. NeuroQuantology 2022;20(10):2654-62. doi: https://dx.doi.org/10.14704/nq.2022.20.10.NQ55228	Software not commercially available
46. Pyrros A, Chen A, Rodriguez-Fernandez JM, et al. Deep Learning-Based Digitally Reconstructed Tomography of the Chest in the Evaluation of Solitary Pulmonary Nodules: A Feasibility Study. Acad Radiol 2022;09:09. doi: https://dx.doi.org/10.1016/j.acra.2022.05.005	Software not commercially available
47. Qiao J, Fan Y, Zhang M, et al. Ensemble framework based on attributes and deep features for benign-malignant classification of lung nodule. Biomedical Signal Processing and Control 2023;79:104217. doi: https://dx.doi.org/10.1016/j.bspc.2022.104217	Software not commercially available
48. Ramana K, Kumar MR, Sreenivasulu K, et al. Early Prediction of Lung Cancers Using Deep Saliency Capsule and Pre-Trained Deep Learning Frameworks. Front 2022;12:886739. doi: https://dx.doi.org/10.3389/fonc.2022.886739	Software not commercially available
49. Ranjitha UN, Gowtham MA. BCDU-Net and chronological-AVO based ensemble learning for lung nodule segmentation and classification. Computer Methods in Biomechanics and Biomedical Engineering: Imaging and Visualization 2022 doi: https://dx.doi.org/10.1080/21681163.2022.2150891	Software not commercially available
50. Rocha J, Cunha A, Mendonca AM. Comparison of Conventional and Deep Learning Based Methods for Pulmonary Nodule Segmentation in CT Images. 19th EPIA Conference on Artificial Intelligence (EPIA); 2019 Sep 03-06; Univ Tras Os Montes & Alto Douro, Vila Real, PORTUGAL. Springer International Publishing Ag.	Software not commercially available
51. Shao J, Wang G, Yi L, et al. Deep Learning Empowers Lung Cancer Screening Based on Mobile Low-Dose Computed Tomography in Resource-Constrained Sites. Front Biosci (Landmark Ed) 2022;27(7):212. doi: https://dx.doi.org/10.31083/j.fbl2707212	Software not commercially available
52. Suresh S, Mohan S. NROI based feature learning for automated tumor stage classification of pulmonary lung nodules using deep convolutional neural networks. J King Saud Univ-Comput Inf Sci 2022;34(5):1706-17. doi: 10.1016/j.jksuci.2019.11.013	Software not commercially available
53. Tan H, Bates JHT, Kinsey CM. Discriminating TB lung nodules from early lung cancers using deep learning. Bmc Medical Informatics and Decision Making 2022;22(1):7. doi: 10.1186/s12911-022-01904-8	Software not commercially available
54. Tandon R, Agarwal S, Arthur C, et al. VCNet: Hybrid Deep Learning Model for Detection and Classification of Lung Carcinoma Using Chest Radiographs. Front 2022;10:13. doi: 10.3389/fpubh.2022.894920	Software not commercially available
55. Tao G, Zhu L, Chen Q, et al. Prediction of future imagery of lung nodule as growth modeling with follow-up computed tomography scans using deep learning: a retrospective cohort study. Transl 2022;11(2):250-62. doi: https://dx.doi.org/10.21037/tlcr-22-59	Software not commercially available
56. Verhoeven R, Van Ooijen PMA, Cnossen F. Designing for appropriate trust in artificial intelligence: making machine learning usable for radiologists. Insights imaging 2022;14(Supplement 4):166. doi: https://dx.doi.org/10.1186/s13244-022-01337-x	Software not commercially available
57. Wang H, Li Y, Liu S, et al. Design Computer-Aided Diagnosis System Based on Chest CT Evaluation of Pulmonary Nodules. Comput 2022;2022:7729524. doi: https://dx.doi.org/10.1155/2022/7729524	Software not commercially available
58. **Wang H, Tang N, Zhang C, et al. Practice toward standardized performance testing of computer-aided detection algorithms for pulmonary nodule. Front 2022;10:1071673. doi: https://dx.doi.org/10.3389/fpubh.2022.1071673**	Software not commercially available
59. **Wang H, Zhu H, Ding L. Accurate classification of lung nodules on CT images using the TransUnet. Front 2022;10:1060798. doi: https://dx.doi.org/10.3389/fpubh.2022.1060798**	Software not commercially available

Reference	Main reason for exclusion
60. **Wang Q, Xu S, Zhang G, et al. Applying a CT texture analysis model trained with deep-learning reconstruction images to iterative reconstruction images in pulmonary nodule diagnosis. J appl clin med phys 2022;23(11):e13759. doi: https://dx.doi.org/10.1002/acm2.13759**	Software not commercially available
61. **Wang X, Gao M, Xie J, et al. Development, Validation, and Comparison of Image-Based, Clinical Feature-Based and Fusion Artificial Intelligence Diagnostic Models in Differentiating Benign and Malignant Pulmonary Ground-Glass Nodules. Front 2022;12:892890. doi: https://dx.doi.org/10.3389/fonc.2022.892890**	Software not commercially available
62. Wang X, Yu Z, Wang L, et al. An Enhanced Priori Knowledge GAN for CT Images Generation of Early Lung Nodules with Small-Size Labelled Samples. Oxid Med Cell Longev 2022;2022:2129303. doi: https://dx.doi.org/10.1155/2022/2129303	Software not commercially available
63. Wang Y, Zhou C, Chan HP, et al. Hybrid U-Net-based deep learning model for volume segmentation of lung nodules in CT images. Med Phys 2022;49(11):7287-302. doi: https://dx.doi.org/10.1002/mp.15810	Software not commercially available
64. Warkentin MT, Al-Sawaihey H, Lam S, et al. Radiomics analysis to predict pulmonary nodule malignancy using machine learning approaches. medRxiv 2022 doi: https://dx.doi.org/10.1101/2022.10.03.22280659	Software not commercially available
65. Weihuan L, Haiyu Z, Shaowei W, et al. Preliminary Study on the Prediction of Pathological Grading of High-Risk Pulmonary Nodules <=3 cm Based on CT Imaging Deep Learning. American Journal of Respiratory and Critical Care Medicine 2022;205(1) doi: https://dx.doi.org/10.1164/ajrccm-conference.2022.205.1_MeetingAbstracts.A2579	Software not commercially available
66. Xia XP, Zhang RF, Yao XF, et al. A novel lung nodule accurate detection of computerized tomography images based on convolutional neural network and probability graph model. Comput Intell 2022;38(5):1728-47. doi: 10.1111/coin.12531	Software not commercially available
67. **Xu J, Ren H, Cai S, et al. An improved faster R-CNN algorithm for assisted detection of lung nodules. Comput Biol Med 2023;153:106470. doi: https://dx.doi.org/10.1016/j.compbiomed.2022.106470**	Software not commercially available
68. Xu R, Luo Y, Du B, et al. LSSANet: A Long Short Slice-Aware Network for Pulmonary Nodule Detection. 25th International Conference on Medical Image Computing and Computer Assisted Intervention (MICCAI); 2022 Sep 18-22; Singapore, SINGAPORE. Springer International Publishing Ag.	Software not commercially available
69. Yang Y, Li XQ, Fu JP, et al. 3D multi-view squeeze-and-excitation convolutional neural network for lung nodule classification. Med Phys:12. doi: 10.1002/mp.16221	Software not commercially available
70. Zhang GL, Zhang HJ, Yao YH, et al. Attention-Guided Feature Extraction and Multiscale Feature Fusion 3D ResNet for Automated Pulmonary Nodule Detection. IEEE Access 2022;10:61530-43. doi: 10.1109/access.2022.3182104	Software not commercially available
71. Zhang H, Zhang H. LungSeek: 3D Selective Kernel residual network for pulmonary nodule diagnosis. Visual Comput 2023;39(2):679-92. doi: https://dx.doi.org/10.1007/s00371-021-02366-1	Software not commercially available
72. Zhang Q, Wang H, Yoon SW, et al. Lung Nodule Diagnosis on 3D Computed Tomography Images Using Deep Convolutional Neural Networks. 25th International Conference on Production Research Manufacturing Innovation (ICPR) - Cyber Physical Manufacturing; 2019 Aug 09-14; Chicago, IL. Elsevier Science Bv.	Software not commercially available
73. Zhao Y, Liu X, Chen Q, et al. Pulmonary Nodule Detection Based on Multiscale Feature Fusion. Computational and Mathematical Methods in Medicine 2022;2022:8903037. doi: https://dx.doi.org/10.1155/2022/8903037	Software not commercially available

Reference	Main reason for exclusion
74. Zheng S, Cornelissen LJ, Cui X, et al. Deep convolutional neural networks for multi-planar lung nodule detection: improvement in small nodule identification. Med Phys 2020 doi: https://dx.doi.org/10.1002/mp.14648	Software not commercially available
75. Zhou Z, Gou F, Tan Y, et al. A Cascaded Multi-Stage Framework for Automatic Detection and Segmentation of Pulmonary Nodules in Developing Countries. IEEE j 2022;26(11):5619-30. doi: https://dx.doi.org/10.1109/JBHI.2022.3198509	Software not commercially available
**Excluded on technology: No AI-based software with appropriate regulatory approval (CE-mark) across the UK and the EU (n=3)**	
76. Chao HS, Tsai CY, Chou CW, et al. Artificial Intelligence Assisted Computational Tomographic Detection of Lung Nodules for Prognostic Cancer Examination: A Large-Scale Clinical Trial. Biomedicines 2023;11(1):06. doi: https://dx.doi.org/10.3390/biomedicines11010147	No CE mark
77. Pinto E, Penha D, Hochhegger B, et al. Variability of pulmonary nodule volumetry on coronary CT angiograms. Medicine (United States) 2022;101(35):E30332. doi: https://dx.doi.org/10.1097/MD.0000000000030332	Not AI-based
78. Smith D, Melville P, Fozzard N, et al. Artificial intelligence software in pulmonary nodule assessment. J R Coll Physicians Edinb 2022;52(3):228-31. doi: https://dx.doi.org/10.1177/14782715221123856	Phillips Lung Nodule software (Build 9.0.3.31331)
**Excluded on outcomes: Clinical trial register, no outcomes reported (n=3)**	
79. CTRI/2022/04/041873. A study to evaluate the effectiveness of computer artificial inteligence in identifying and classifying abnormalites in chest radiographs: http://www.ctri.nic.in/Clinicaltrials/pmaindet2.php?trialid=67663, 2022.	Clinical trial register, no outcomes reported
80. CTRI/2022/11/047695. Development and Validation of Artificial Intelligence Based Tool to read Chest X-rays in order to detect Pulmonary TB and other lung diseases: http://www.ctri.nic.in/Clinicaltrials/pmaindet2.php?trialid=76660, 2022.	Clinical trial register, no outcomes reported
81. ClinicalTrials. Evaluation of Contextflow DETECT Lung CT Nodule Detection Software in Chest CT Scans: https://ClinicalTrials.gov/show/NCT05481762, 2022.	Clinical trial register, no outcomes reported
**Excluded on outcomes: No relevant test accuracy outcomes reported (n=6)**	
82. Lancaster H, Zheng S, Aleshina O, et al. INTER-READER AGREEMENT WHEN USING ARTIFICIAL INTELLIGENCE FOR CLASSIFICATION OF SOLID PULMONARY NODULES IN ULTRA-LOW DOSE CT BASELINE LUNG CANCER SCREENING. Chest 2022;161(6 Supplement):A565. doi: https://dx.doi.org/10.1016/j.chest.2022.04.099	No relevant test accuracy outcomes reported
83. Lv J, Li J, Liu Y, et al. Artificial Intelligence-Aided Diagnosis Software to Identify Highly Suspicious Pulmonary Nodules. Front 2021;11:749219. doi: https://dx.doi.org/10.3389/fonc.2021.749219	No relevant test accuracy outcomes reported
84. Ottilinger T, Martini K, Baessler B, et al. Semi-automated volumetry of pulmonary nodules: Intra-individual comparison of standard dose and chest X-ray equivalent ultralow dose chest CT scans. Eur J Radiol 2022;156:110549. doi: https://dx.doi.org/10.1016/j.ejrad.2022.110549	No relevant test accuracy outcomes reported
85. Shu J, Wen D, Xu Z, et al. Improved interobserver agreement on nodule type and Lung-RADS classification of subsolid nodules using computer-aided solid component measurement. Eur J Radiol 2022;152:110339. doi: https://dx.doi.org/10.1016/j.ejrad.2022.110339	No relevant test accuracy outcomes reported
86. Wataya T, Yanagawa M, Tsubamoto M, et al. Radiologists with and without deep learning-based computer-aided diagnosis: comparison of performance and interobserver agreement for characterizing and diagnosing pulmonary nodules/masses. Eur Radiol 2023;33(1):348-59. doi: https://dx.doi.org/10.1007/s00330-022-08948-4	No relevant test accuracy outcomes reported
87. Yacoub B, Varga-Szemes A, Joseph Schoepf U, et al. Impact of Artificial Intelligence Assistance on Chest CT Interpretation Times: A Prospective Randomized Study. American Journal of Roentgenology 2022;219(5):743-51. doi: https://dx.doi.org/10.2214/AJR.22.27598	No relevant test accuracy outcomes reported
**Excluded on publication type: Conference abstract with no additional data to an also included full-text paper (n=21)**	

Reference	Main reason for exclusion
88. Agarwal P, Mueller-Peltzer K, Kotter E. The next generation of reference books: combining content-based image retrieval with a knowledge-based diagnostic decision support system in chest CT. Insights imaging 2022;14(Supplement 4):248. doi: https://dx.doi.org/10.1186/s13244-022-01337-x	Conference abstract with no additional data reported
89. Ananth S, Navarra A, Mogal R, et al. Machine learning can predict lung cancer using primary care data. Lung Cancer 2022;165(Supplement 1):S23. doi: https://dx.doi.org/10.1016/S0169-5002%2822%2900095-2	Conference abstract with no additional data reported
90. Baudot P, Voyton CM, Francis D, et al. Development and validation of a machine learning based CADx designed to improve patient management in lung cancer screening programmes. Insights imaging 2022;14(Supplement 4):253. doi: https://dx.doi.org/10.1186/s13244-022-01337-x	Conference abstract with no additional data reported
91. Calhoun ME, Hofmanninger J, Wood C, et al. EP13.01-011 Combining Automated Malignancy Risk Estimation with Lung Nodule Detection May Reduce Physician Effort and Increase Diagnostic Accuracy. Journal of Thoracic Oncology 2022;17(9 Supplement):S523. doi: https://dx.doi.org/10.1016/j.jtho.2022.07.931	Conference abstract with no additional data reported
92. Cimini A, Ricci M, Chiaravalloti A, et al. Incisive: a multimodal ai-based toolbox for the empowerment of imaging analysis related to the diagnosis, prediction and followup of cancer. Clinical and Translational Imaging 2022;10(SUPPL 1):S94. doi: https://dx.doi.org/10.1007/s40336-022-00492-x	Conference abstract with no additional data reported
93. De Mattia C, Calderoni F, Colombo PE, et al. Impact of reconstruction algorithm on a Computer Aided Detection (CAD) system: comparison of the lung lesion contouring and of the texture analysis. Phys Med 2021;92(Supplement):S101. doi: https://dx.doi.org/10.1016/S1120-1797%2822%2900215-0	Conference abstract with no additional data reported
94. Gimbel I, Bergsma M, Weijer MVD, et al. Earlier discharge of patients from follow-up for lung cancer screening using artificial intelligence for detection of pulmonary noduli on computed tomography. Insights imaging 2022;14(Supplement 4):125. doi: https://dx.doi.org/10.1186/s13244-022-01337-x	Conference abstract with no additional data reported
95. Huang G, Zhang Y, Xue D. Artificial Intelligence Assisted Diagnosis Technology For Benign-Malignant Lung Nodule Classification On Computerized Tomography Images: A Meta-Analysis. International Journal of Technology Assessment in Health Care 2020;36(Supplement 1):14-15. doi: https://dx.doi.org/10.1017/S0266462320001245	Conference abstract with no additional data reported
96. Kuhl PJ, Gruschwitz P, Heidenreich JF, et al. Performance and dose dependency of computer-aided detection (CAD) of pulmonary nodules in tin-filtered paediatric ultra low dose chest CT. Insights imaging 2022;14(Supplement 4):343. doi: https://dx.doi.org/10.1186/s13244-022-01337-x	Conference abstract with no additional data reported
97. Mandal S, Sathyamurthy S, Govindarajan A, et al. PP01.58 Multi City Opportunistic Screening of Lung Nodules amidst COVID-19. Journal of Thoracic Oncology 2023;18(3 Supplement):e35. doi: https://dx.doi.org/10.1016/j.jtho.2022.09.084	Conference abstract with no additional data reported
98. Pirgulov S. Effectiveness of artificial intelligence in retrospective COVID-19 lung CT analysis for lung cancer detection. Ann Oncol 2022;33(Supplement 2):S79. doi: https://dx.doi.org/10.1016/j.annonc.2022.02.129	Conference abstract with no additional data reported
99. Rosell A, Baeza S, Garcia-Reina S, et al. EP01.05-001 Radiomics to Increase the Effectiveness of Lung Cancer Screening Programs. Radiolung Preliminary Results. Journal of Thoracic Oncology 2022;17(9 Supplement):S182. doi: https://dx.doi.org/10.1016/j.jtho.2022.07.302	Conference abstract with no additional data reported
100. Santos RSD, Teles GBDS, Chate RC, et al. MA11.09 Artificial Intelligence in Lung Cancer Screening: Accuracy and Predictive Value. Journal of Thoracic Oncology 2022;17(9 Supplement):S83-S84. doi: https://dx.doi.org/10.1016/j.jtho.2022.07.142	Conference abstract with no additional data reported
101. Song JY, Kim Y, Lee N, et al. EP01.04-003 Effectiveness of Cloud-based Computer Aided Quality Control System in Korean National Lung Cancer Screening. Journal of Thoracic Oncology 2022;17(9 Supplement):S179-S80. doi: https://dx.doi.org/10.1016/j.jtho.2022.07.298	Conference abstract with no additional data reported

Reference	Main reason for exclusion
102. Stav D, Balcombe J, Aviram G, et al. Detection of unreported clinically significant pulmonary nodules using a combination of computer vision (CV) algorithm and report processing. Insights imaging 2022;14(Supplement 4):125. doi: https://dx.doi.org/10.1186/s13244-022-01337-x	Conference abstract with no additional data reported
103. Takaishi T. THE USE OF ARTIFICIAL INTELLIGENCE SYSTEM TO DETECT LUNG NODULES ON CT SCAN IN REAL CLINICAL SETTINGS. Chest 2022;161(1 Supplement):A319. doi: https://dx.doi.org/10.1016/j.chest.2021.12.349	Conference abstract with no additional data reported
104. Venkadesh KV, Aleef TA, Schreuder A, et al. Deep learning for estimating pulmonary nodule malignancy risk using prior CT examinations in lung cancer screening. Insights imaging 2022;14(Supplement 4):5. doi: https://dx.doi.org/10.1186/s13244-022-01337-x	Conference abstract with no additional data reported
105. Verhoeven R, Van Ooijen PMA, Cnossen F. Designing for appropriate trust in artificial intelligence: making machine learning usable for radiologists. Insights imaging 2022;14(Supplement 4):166. doi: https://dx.doi.org/10.1186/s13244-022-01337-x	Conference abstract with no additional data reported
106. Yuan R, Mayo J, Myers R, et al. MA11.08 Value of Computer Aided Diagnosis on Radiologists' Workflow and Recommendation for Reporting Lung Cancer Screening LDCT. Journal of Thoracic Oncology 2022;17(9 Supplement):S83. doi: https://dx.doi.org/10.1016/j.jtho.2022.07.141	Conference abstract with no additional data reported
107. Zhang Y, Han B, Qian F. EP01.03-010 China Lung Cancer Screening Study (CLUS) Version 2.0: Study Design and Baseline Screening Results. Journal of Thoracic Oncology 2022;17(9 Supplement):S176. doi: https://dx.doi.org/10.1016/j.jtho.2022.07.292	Conference abstract with no additional data reported
108. Zinovev SV. The potential for lung cancer detection in COVID-19 CT scans with AI technologies usage. Ann Oncol 2022;33(Supplement 2):S79. doi: https://dx.doi.org/10.1016/j.annonc.2022.02.130	Conference abstract with no additional data reported
**Excluded on publication type: No primary research article (n=7)**	
109. Boon IS, Teo RPJ, Yap MH, et al. Re: Clinical evaluation of a deep-learning-based computer-aided detection system for the detection of pulmonary nodules in a large teaching hospital. Clin Radiol 2022;77(2):156-57. doi: https://dx.doi.org/10.1016/j.crad.2021.10.025	Letter
110. Forte GC, Altmayer S, Silva RF, et al. Deep Learning Algorithms for Diagnosis of Lung Cancer: A Systematic Review and Meta-Analysis. Cancers (Basel) 2022;14(16):3856. doi: https://dx.doi.org/10.3390/cancers14163856	Systematic review
111. Grenier PA, Brun AL, Mellot F. The Potential Role of Artificial Intelligence in Lung Cancer Screening Using Low-Dose Computed Tomography. Diagnostics (Basel) 2022;12(10):08. doi: https://dx.doi.org/10.3390/diagnostics12102435	Review
112. Jin H, Yu C, Gong Z, et al. Machine learning techniques for pulmonary nodule computer-aided diagnosis using CT images: A systematic review. Biomedical Signal Processing and Control 2023;79:104104. doi: https://dx.doi.org/10.1016/j.bspc.2022.104104	Systematic review
113. Li R, Xiao C, Huang Y, et al. Deep Learning Applications in Computed Tomography Images for Pulmonary Nodule Detection and Diagnosis: A Review. Diagnostics (Basel) 2022;12(2):25. doi: https://dx.doi.org/10.3390/diagnostics12020298	Review
114. Thong LT, Chou HS, Chew HSJ, et al. Diagnostic test accuracy of artificial intelligence-based imaging for lung cancer screening: A systematic review and meta-analysis. Lung Cancer 2023;176:4-13. doi: https://dx.doi.org/10.1016/j.lungcan.2022.12.002	Systematic review
115. Zhang K, Wei Z, Nie Y, et al. Comprehensive Analysis of Clinical Logistic and Machine Learning-Based Models for the Evaluation of Pulmonary Nodules. JTO Clin Res Rep 2022;3(4):100299. doi: https://dx.doi.org/10.1016/j.jtocrr.2022.100299	Review
**Excluded on publication date: Published before 2012 (n=2)**	
116. Beyer F, Heindel W, Wormanns D. Acoustical markers for CAD-detected pulmonary nodules in chest CT: a way to avoid suggestion and distraction of radiologist's attention? Conference on Medical Imaging - Computer-Aided Diagnosis; 2009 Feb 10-12; Lake Buena Vista, FL. Spie-Int Soc Optical Engineering.	Published before 2012
117. Dolejsi M, Kybic J, Polovincak, et al. The Lung TIME-Annotated Lung Nodule Dataset and Nodule Detection Framework. Conference on Medical Imaging - Computer-Aided Diagnosis; 2009 Feb 10-12; Lake Buena Vista, FL. Spie-Int Soc Optical Engineering.	Published before 2012

Reference	Main reason for exclusion
**Excluded on language: Non-English (n=1)**	
118. Zhang K, Wei ZH, Wang X, et al. The diagnostic value of machine-learning-based model for predicting the malignancy of solid nodules in multiple pulmonary nodules. Zhonghua wai ke za zhi [Chinese journal of surgery] 2022;60(6):573-79. doi: 10.3760/cma.j.cn112139-20211101-00511	Chinese language
**Excluded: Already identified via other sources (n=2)**	
119. Hall H, Ruparel M, Quaife SL, et al. The role of computer-assisted radiographer reporting in lung cancer screening programmes. Eur Radiol 2022;32(10):6891-99. doi: https://dx.doi.org/10.1007/s00330-022-08824-1	Already identified via other sources
120. **Lancaster HL, Zheng S, Aleshina OO, et al. Outstanding negative prediction performance of solid pulmonary nodule volume AI for ultra-LDCT baseline lung cancer screening risk stratification. Lung Cancer 2022;165:133-40. doi: https://dx.doi.org/10.1016/j.lungcan.2022.01.002**	Already identified via other sources
**Excluded: Duplicate (n=1)**	
121. Song JY, Kim Y, Lee N, et al. Effectiveness of Cloud-based Computer Aided Quality Control System in Korean National Lung Cancer Screening. Journal of Thoracic Oncology 2022;17(9):S179-S80.	Duplicate
**Excluded: Concerns related to reported methods and results, and no author reply (n=1)**	
122. **Wang D, Cao L, Li B. Computer-aided diagnosis system versus conventional reading system in low-dose (< 2 mSv) computed tomography: comparative study for patients at risk of lung cancer. Sao Paulo Medical Journal = Revista Paulista de Medicina 2022;28:28. doi: https://dx.doi.org/10.1590/1516-3180.2022.0130.R1.29042022**	Concerns related to reported methods and results, and no author reply.

Supplementary material 6: Quality assessment results based on QUADAS-2 and QUADAS-C tools (10 studies)

	Test	Risk of bias (QUADAS-2)						Applicability concerns (QUADAS-2)				Risk of bias (QUADAS-C)					
		P	I	R		F&T		P	I	R		P	I	R		F&T	
				Nodule	Cancer	Nodule	Cancer			Nodule	Cancer			Nodule	Cancer	Nodule	Cancer
**Hall 2022**	C	Unclear	High	High	Unclear	High	Unclear	Low	High	Low	Unclear	Unclear	High	High		High	
	E	Low	Low	High		Low		Low	High	Low							
**Hsu 2021**	A	High	Unclear	High		Low		High	High	High		High	High	High		Low	
	B	High	High	High		Low		High	High	High							
	C	High	High	High		Low		High	High	High							
	D	High	High	High		Low		High	High	High							
**Hwang 2021a**	A	Unclear	Low	High	High	High	Unclear	High	High	High	High	High	Low		High		Unclear
	C	Unclear	Low		High		Unclear	High	Low		High						
	E	Low	Low		High		Unclear	High	Low		High						
**Lancaster 2022**	A	High	Low	High		Low		High	High	Low		High	High	High		Low	
	C	High	High	High		Low		High	Low	Low							
	D	High	High	High		Low		High	Low	Low							
**Lo 2018**	A	High	Unclear	Low	Low	Low	High	High	High	Low	Low	High	High	Low	Low	Low	High
	C	High	High	Low	Low	Low	High	High	High	Low	Low						
	D	High	High	Low	Low	Low	High	High	High	Low	Low						
**Park 2022**	A	High	Unclear		Unclear		Unclear	High	High		Unclear	High	High		Unclear		Unclear
	C	High	High		Unclear		Unclear	High	High		Unclear						
	D	High	High		Unclear		Unclear	High	High		Unclear						
**Singh 2021**	A	High	Unclear	Low		High		High	High	Low		High	High	Low		High	
	C.1	High	High	Low		High		High	Unclear	Low							
	D	High	High	Low		High		High	Low	Low							
**Zhang 2021**	C	Low	High	High		Low		High	High	High		Low	High	High		Low	
	D	Low	Low	High		Low		High	High	High							
**Non-comparative accuracy studies**																	
**Chamberlin 2021**	A	Low	Low	High		High		High	High	High							
**Hwang 2021b**	C	Unclear	Low		High		High	High	Low		High						

A, Stand-alone AI; B, Assisted 2nd-read AI; C, Concurrent AI; C.1, Concurrent AI for vessel-suppression; D, Unaided reader (Reader study); E, Original radiologist (clinical practice); F&T, Flow & timing; I, Index test; P, Population; R, Reference standard.
One included study (Jacobs et al. 2021) reporting shifts in Lung-RADS categorisation was not assessed using QUADAS tools as it did not include a reference standard.

Further details of risk of bias and applicability assessment

In the *population domain*, the risk of bias was high in 6/10 studies as no consecutive or random sample was included and a case control design was used [1-4] because of inappropriate exclusions [1, 5], or because no fully paired or randomized design was used [6].

In the *index test domain*, 7/10 studies were rated as having high risk of bias as the CT images were not assessed by human readers in clinical practice [1-5, 7, 8].

In the *reference standard domain* (lung nodule presence/absence), 6/8 studies had a high risk of bias as no majority of (at least) three experienced chest radiologists was used as reference standard, and/or the reference standard reader(s) were part of the index test or not blinded to index test markings / decisions [1, 5-9]. For the presence/absence of lung cancer, the reference standard domain was rated as high risk of bias in 2/5 studies as medical records were used, and the clinicians undertaking the diagnostic follow up tests were not blinded to the results of the index test [6, 10].

Study flow & timing was rated as high risk of bias in 4/8 studies evaluating the detection of lung nodules as exclusions from the analysis were higher than 10%[4, 7] or as the number of CT images excluded due to software processing failures (e.g. segmentation failures) has not been reported [6, 9]. For the five studies on the detection of lung cancer, flow & timing was rated as high risk in two studies as not all patients received a reference standard [10] or not all patients received the same reference standard [2].

The applicability to potential European [11] or UK [12] lung cancer screening programmes was low to moderate. All 10 studies had 'high' applicability concerns in at least one of the three domains (i.e. population, index test, reference standard).

Concerns regarding the applicability of the *population* to the situation in Europe were classified as high in 9/10 included studies as the included study populations/CT images were not from Europe but from Asia [5, 6, 8, 10] and the USA [2-4, 9], the sample was not consecutive or random and enriched for nodules [1-4], and the participants were not current or former heavy smokers aged between 50 and 75 years [1, 5, 8, 9].

Concerns regarding the applicability of the *index test(s)* to the situation in Europe were classified as high for at least one index test in 9/10 included studies as the integration of software into the pathway was not applicable to anticipated EU practice (e.g. stand-alone AI performance instead of concurrent or second-read software use) [1-6, 9], the study did not use pre-specified nodule size thresholds that were in line with commonly used nodule management guidelines for screening LDCT

images from the BTS [13] (≥5mm diameter or ≥80mm^3), Lung-RADS[14] (category ≥3) or the European Position Statement (EUPS) [15] (≥5 mm or ≥100 mm^3) [5, 6, 8, 9] more than one human reader was involved per read for AI-assisted reading and/or the unassisted human comparator [7, 8], and human reader's experience and/or specialty was not representative of anticipated European clinical practice (i.e. experienced chest radiologists) [2, 3, 5, 7, 8]. In four studies, the applicability concerns regarding index tests that involve human readers (with or without AI) were rated as low [1, 4, 6, 10].

Applicability concerns regarding the *reference standard for lung nodules* were rated high in 4/8 studies as the definition of "actionable nodules" was not in line with commonly used nodule management guidelines for screening LDCT images from the BTS[13] (≥5mm diameter or ≥80mm^3), Lung-RADS [14] (category ≥3) or the EUPS [15] (≥5 mm or ≥100 mm^3) [5, 6, 8, 9]. Applicability concerns regarding the *reference standard for lung cancer* were high for 2/5 studies as there was no follow-up for at least two years for discharged patients (i.e. not receiving CT surveillance or biopsy/excision) [6, 10].

References for supplementary material 6

1. Lancaster HL, Zheng S, Aleshina OO, et al. Outstanding negative prediction performance of solid pulmonary nodule volume AI for ultra-LDCT baseline lung cancer screening risk stratification. *Lung Cancer* 2022;165:133-40. doi: 10.1016/j.lungcan.2022.01.002

2. Lo SB, Freedman MT, Gillis LB, et al. Journal club: computer-aided detection of lung nodules on ct with a computerized pulmonary vessel suppressed function. *AJR Am J Roentgenol* 2018;210(3):480-88. doi: 10.2214/AJR.17.18718

3. Park S, Park H, Lee SM, et al. Application of computer-aided diagnosis for Lung-RADS categorization in CT screening for lung cancer: effect on inter-reader agreement. *Eur Radiol* 2022;32:1054–64. doi: 10.1007/s00330-021-08202-3

4. Singh R, Kalra MK, Homayounieh F, et al. Artificial intelligence-based vessel suppression for detection of sub-solid nodules in lung cancer screening computed tomography. *Quant Imaging Med Surg* 2021;11(4):1134-43. doi: 10.21037/qims-20-630

5. Hsu HH, Ko KH, Wu YC, et al. Performance and reading time of lung nodule identification on multidetector CT with or without an artificial intelligence-powered computer-aided detection system. *Clin Radiol* 2021;76(8):626. doi: 10.1016/j.crad.2021.04.006

6. Hwang EJ, Goo JM, Kim HY, et al. Implementation of the cloud-based computerized interpretation system in a nationwide lung cancer screening with low-dose CT: comparison with the conventional reading system. *Eur Radiol* 2021;31(1):475-85. doi: 10.1007/s00330-020-07151-7

7. Hall H, Ruparel M, Quaife SL, et al. The role of computer-assisted radiographer reporting in lung cancer screening programmes. *Eur Radiol* 2022;32(10):6891-99. doi: 10.1007/s00330-022-08824-1

8. Zhang Y, Jiang B, Zhang L, et al. Lung nodule detectability of artificial intelligence-assisted CT image reading in lung cancer screening. *Curr Med Imaging* 2021;18(3):327-34. doi: 10.2174/1573405617666210806125953

9. Chamberlin J, Kocher MR, Waltz J, et al. Automated detection of lung nodules and coronary artery calcium using artificial intelligence on low-dose CT scans for lung cancer screening: accuracy and prognostic value. *BMC Med* 2021;19(1):55. doi: 10.1186/s12916-021-01928-3

10. Hwang EJ, Goo JM, Kim HY, et al. Optimum diameter threshold for lung nodules at baseline lung cancer screening with low-dose chest CT: exploration of results from the Korean Lung Cancer Screening Project. *Eur Radiol* 2021;31(9):7202-12. doi: 10.1007/s00330-021-07827-8

11. European Commission. European Health Union: a new EU approach on cancer detection – screening more and screening better. 20 September 2022. https://ec.europa.eu/commission/presscorner/detail/en/ip_22_5562

12. Department of Health and Social Care, Sunak R, Barclay S. Press release: New lung cancer screening roll out to detect cancer sooner. Gov.uk; 26 June 2023. https://www.gov.uk/government/news/new-lung-cancer-screening-roll-out-to-detect-cancer-sooner

13. Callister ME, Baldwin DR, Akram AR, et al. British Thoracic Society guidelines for the investigation and management of pulmonary nodules. *Thorax* 2015;70(Suppl 2):ii1-ii54. doi: 10.1136/thoraxjnl-2015-207168

14. American College of Radiology. Lung CT Screening Reporting & Data System (Lung-RADS). Version 1.1. 2019. https://www.acr.org/Clinical-Resources/Reporting-and-Data-Systems/Lung-Rads

15. Oudkerk M, Devaraj A, Vliegenthart R, et al. European position statement on lung cancer screening. *Lancet Oncol* 2017;18(12):e754-e66. doi: 10.1016/s1470-2045(17)30861-6

Supplementary material 7: Comparative evidence on sensitivity and specificity for AI-assisted reading compared with unaided reading by radiologists for the detection and/or categorisation of lung nodules in CT images for lung cancer screening*

Study and comparison	Sensitivity (95% CI)			Specificity (95% CI)		
	AI-assisted [B] or [C]	Unaided [D] or [E]	Difference (p value)	AI-assisted [B] or [C]	Unaided [D] or [E]	Difference (p value)
**Detection of any nodules**						
Zhang 2021 [C][D]	370/374,<br>0.99 (0.97 to 1.00)	162/374<br>0.43 (0.38 to 0.48)	0.56 (NR)	472/486<br>0.97 (0.95 to 0.98)	486/486<br>1.00 (0.99 to 1.00)	-0.03 (NR)
Hsu 2021 [C][D] [a]	NR<br>0.79 (0.76 to 0.81)	NR<br>(0.63 (0.59 to 0.66)	0.16 (p<0.001)	NR<br>0.81 (0.78 to 0.84)	NR<br>0.77 (0.74 to 0.80)	0.04 (p=0.449)
Hsu 2021 [B][D] [a]	NR<br>0.80 (0.77 to 0.83)	NR<br>0.63 (0.59 to 0.66)	0.17 (p<0.001)	NR<br>0.82 (0.79 to 0.84)	NR<br>0.77 (0.74 to 0.80)	0.05 (p=0.360)
**Detection of actionable nodules**						
Lo 2018 [C][D] [a]	NR<br>0.73 (0.71 to 0.74)	NR<br>0.60 (0.58 to 0.62)	0.13 (p<0.0001)	NR<br>0.84 (0.83 to 0.86)	NR<br>0.90 (0.89 to 0.91)	-0.06 (p=0.0025)
Singh 2021[C.1][D]	NR<br>0.73 (0.70 to 0.77)	NR<br>0.68 (0.64 to 0.72)	0.05 (NR)	NR<br>0.74 (NR)	NR<br>0.77 (NR)	-0.03 (NR)
**Risk classification based on volume**						
Lancaster 2022 [C][D]	213/249<br>0.86 (0.81 to 0.89)	109/166<br>0.66 (0.58 to 0.72)	0.20 (NR)	528/600<br>0.88 (0.85 to 0.90)	379/400<br>0.95 (0.92 to 0.97)	-0.07 (NR)
**Detection of malignant nodules**						
Lo 2018 [C][D] [a]	NR<br>0.80 (0.70 to 0.87)	NR<br>0.65 (0.54 to 0.74)	0.15 (p<0.0001)	NR<br>0.84 (0.79 to 0.89)	NR<br>0.90 (0.85 to 0.93)	-0.06 (p=0.0025)
**Nodule detection plus risk categorisation for lung cancer detection**						
Park 2022 [C][D]	142/155<br>0.92 (0.86 to 0.95)	132/155<br>0.85 (0.79 to 0.90)	0.07 (p=0.004)	645/845<br>0.76 (0.73 to 0.79)	692/845<br>0.82 (0.79 to 0.84)	-0.06 (NR)
Hwang 2021a [C][E]	30/31<br>0.97 (0.84 to 0.99)	15/16<br>0.94 (0.72 to 0.99)	0.03 (NR)	3,853/4,635<br>0.83 (0.82 to 0.84)	1,640/1,805<br>0.91 (0.89 to 0.92)	-0.08 (NR)

[B] Second-read AI: CT scan image was firstly reviewed by an unaided human reader, then was re-interpreted after analysis by AI software was shown; [C] Concurrent AI: CT scan image was reviewed by a human reader assisted by concurrent display of analysis by AI software; [C.1] Concurrent AI with vessel suppression function only; [D] Unaided reader: CT scan image was reviewed by a human reader without assisted by AI software; [E] Original unaided reader: CT scan image was interpreted by a human reader as part of clinical practice, and therefore the reader was different from the human reader who interpret the CT scan image in the reader study.

NR, Not reported.

[a] In multiple reader multiple case laboratory studies where multiple readers asses the same images, there are considerable problems in summing 2x2 test data across readers.

*Hall 2022 was not included here as the nature of comparison (AI-assisted reading by radiographers vs unaided radiologists) was different.

Supplementary material 8: Evidence on AI-assisted reading compared with unaided reading and stand-alone AI for accuracy of detecting and/or categorising any nodules, actionable nodules and malignant nodules

Keys for the figure:

TP: true positive; FP: false positive; FN: false negative; TN: true negative.

Index test and comparators: [A] Stand-alone AI: analysis of CT scan image by AI software without human input; [B] Second-read AI: CT scan image was firstly reviewed by an unaided human reader, then was re-interpreted after analysis by AI software was shown; [C] Concurrent AI: CT scan image was reviewed by a human reader assisted by concurrent display of analysis by AI software; [D] Unaided reader: CT scan image was reviewed by a human reader without assisted by AI software; [E] Original unaided reader: CT scan image was interpreted by a human reader as part of clinical practice, and therefore the reader was different from the human reader who interpret the CT scan image in the reader study.

For Lancaster 2022, the test accuracy data only concerned categorisation of nodules by volume (≥100 mm^3 vs <100 mm^3) rather than ”detection and categorisation”, as all CT scan images included in the study sample had lung nodules.

For Lo 2018, cancer was considered to have been detected if the study radiologists placed a mark within the original marking for actionable nodules determined by experts as reference standard. A judgement of whether the marked nodule was actionable did not appear to be required and therefore the accuracy data seemed to be concerned only with detection rather than “detection and categorisation”.

Studysubset	Country	Software	Reader	TP	FP	FN	TN	Sensitivity (95% CI)	Specificity (95% CI)
Any nodules									
Zhang 2021 [C]	China	InferRead CT Lung	Concurrent AI	370	14	4	472	0.99 (0.97, 1.00)	0.97 (0.95, 0.98)
Zhang 2021 [D]	China	InferRead CT Lung	Unaided reader	162	0	212	486	0.43 (0.38, 0.48)	1.00 (0.99, 1.00)
Hsu 2021 [C]	Taiwan	ClearRead CT	Concurrent AI					0.79 (0.76, 0.81)	0.81 (0.78, 0.84)
Hsu 2021 [B]	Taiwan	ClearRead CT	Second-read AI					0.80 (0.77, 0.83)	0.82 (0.79, 0.84)
Hsu 2021 [D]	Taiwan	ClearRead CT	Unaided reader					0.63 (0.59, 0.66)	0.77 (0.74, 0.80)
Actionable nodules									
Chamberlin 2021 [A]	USA	AI-Rad Companion	Stand-alone AI	69	14	0	34	1.00 (0.93, 1.00)	0.71 (0.56, 0.83)
Lo 2018 [C]	USA	ClearRead CT	Concurrent AI					0.73 (0.71, 0.74)	0.84 (0.83, 0.86)
Lo 2018 [D]	USA	ClearRead CT	Unaided Reader					0.60 (0.58, 0.62)	0.90 (0.89, 0.91)
Lo 2018 [A]	USA	ClearRead CT	Stand-alone AI					0.82	
Singh 2021 [C]	USA	ClearRead CT	Concurrent AI	453	73	167		0.73 (0.70, 0.77)	0.74
Singh 2021 [D]	USA	ClearRead CT	Unaided Reader	423	64	197		0.68 (0.64, 0.72)	0.77
Singh 2021 [A]	USA	ClearRead CT	Stand-alone AI		10			0.58	0.82
Hall 2022 [C] radiographer	UK	Veolity	Concurrent AI	217	82	89	1000	0.71 (0.66, 0.76)	0.92 (0.91, 0.94)
Hall 2022 [E] radiologist	UK	Veolity	Unaided Reader	144	19	14	558	0.91 (0.86, 0.95)	0.97 (0.95, 0.98)
Lancaster 2022 [C]	Russia/Netherlands	AVIEW LCS	Concurrent AI	213	72	36	528	0.86 (0.81, 0.89)	0.88 (0.85, 0.90)
Lancaster 2022 [D]	Russia/Netherlands	AVIEW LCS	Unaided Reader	109	21	57	379	0.66 (0.58, 0.72)	0.95 (0.92, 0.97)
Lancaster 2022 [A]	Russia/Netherlands	AVIEW LCS	Stand-alone AI	75	53	8	147	0.90 (0.82, 0.95)	0.74 (0.67, 0.79)
Cancer									
Huang 2021a [C]	South Korea	A View Lungscreen	Concurrent AI	30	782	1	3853	0.97 (0.84, 0.99)	0.83 (0.82, 0.84)
Huang 2021a [E]	South Korea	A View Lungscreen	Unaided Reader	15	165	1	1640	0.94 (0.72, 0.99)	0.91 (0.89, 0.92)
Huang 2021b [C]	South Korea	A View Lungscreen	Concurrent AI	50	857	2	9515	0.96 (0.87, 0.99)	0.92 (0.91, 0.92)
Park 2022 [C]	USA/South Korea	VUNO Med-LungCT AI	Concurrent AI	142	200	13	645	0.92 (0.86, 0.95)	0.76 (0.73, 0.79)
Park 2022 [D]	USA/South Korea	VUNO Med-LungCT AI	Unaided Reader	132	153	23	692	0.85 (0.79, 0.90)	0.82 (0.79, 0.84)
Park 2022 [A]	USA/South Korea	VUNO Med-LungCT AI	Stand-alone AI	31	63	0	106	1.00 (0.89, 1.00)	0.63 (0.55, 0.70)
Hall 2022 [C] radiographer	UK	Veolity	Concurrent AI	54	245	9	1080	0.86 (0.75, 0.92)	0.82 (0.79, 0.84)
Lo 2018 [C]	USA	ClearRead CT	Concurrent AI					0.80	0.84
Lo 2018 [D]	USA	ClearRead CT	Unaided Reader					0.65	0.90
Lo 2018 [A]	USA	ClearRead CT	Stand-alone AI					0.89	

.3 .6 1 .3 .6 1

Supplementary material 9: Illustrative calculations for the potential impact of introducing AI assistance in a lung cancer screening programme

**Detection / categorisation of actionable nodules**							
**Lo et al. 2018 [1]**		Sensitivity		Specificity		Number of participants (CT scan images)	
**AI-assisted**		0.73		0.84		324	
**Unaided**		0.60		0.90		324	
**Predicted outcome** Per 1,000,000 participants							
**Prevalence of people with actionable nodule**		True positive	False positive	True negative	False negative	PPV	NPV
**50%**	**AI-assisted**	365,000	80,000	420,000	135,000	0.82	0.76
	**Unaided**	300,000	50,000	450,000	200,000	0.86	0.69
**20%[a]**	**AI-assisted**	146,000	128,000	672,000	54,000	0.53	0.93
	**Unaided**	120,000	80,000	720,000	80,000	0.60	0.90
**5%**	**AI-assisted**	36,500	152,000	798,000	13,500	0.19	0.98
	**Unaided**	30,000	95,000	855,000	20,000	0.24	0.98
**Singh et al. 2021 [2]**		Sensitivity		Specificity		Number of participants (CT scan images)	
**AI-assisted**		0.73		0.74		123	
**Unaided**		0.68		0.77		123	
**Predicted outcome** Per 1,000,000 participants							
**Prevalence of people with actionable nodule**		True positive	False positive	True negative	False negative	PPV	NPV
**50%**	**AI-assisted**	365,000	130,000	370,000	135,000	0.74	0.73
	**Unaided**	340,000	115,000	385,000	160,000	0.75	0.71
**20%[a]**	**AI-assisted**	146,000	208,000	592,000	54,000	0.41	0.92
	**Unaided**	136,000	184,000	616,000	64,000	0.43	0.91
**5%**	**AI-assisted**	36,500	247,000	703,000	13,500	0.13	0.98
	**Unaided**	34,000	218,500	731,500	16,000	0.13	0.98

NPV, Negative predicted value; PPV, Positive predicted value.

[a] Based on proportion of baseline scans requiring CT surveillance in the NELSON trial (19.7%)[3].

Detection / categorisation of malignant nodules							
**Hwang et al. 2021a [4]**		Sensitivity		Specificity		Number of participants (CT scan images)	
**AI-assisted**		0.97		0.83		3853	
**Unaided**		0.94		0.91		1640	
**Predicted outcome** Per 1,000,000 participants							
**Prevalence of people with malignant nodule (per 1000)**		True positive	False positive	True negative	False negative	PPV	NPV
**10**	**AI-assisted**	9,700	168,300	821,700	300	0.05	0.99964
	**Unaided**	9,400	89,100	900,900	600	0.10	0.99933
**5[b]**	**AI-assisted**	4,850	169,150	825,850	150	0.03	0.99982
	**Unaided**	4,700	89,550	905,450	300	0.05	0.99967
**1**	**AI-assisted**	970	169,830	829,170	30	0.01	0.99996
	**Unaided**	940	89,910	909,090	60	0.01	0.99993
**Park et al. 2022 [5]**		Sensitivity		Specificity		Number of participants (CT scan images)	
**AI-assisted**		0.92		0.76		200	
**Unaided**		0.85		0.82		200	
**Predicted outcome** Per 1,000,000 participants							
**Prevalence of people with malignant nodule (per 1000)**		True positive	False positive	True negative	False negative	PPV	NPV
**10**	**AI-assisted**	9,200	237,600	752,400	800	0.04	0.99894
	**Unaided**	8,500	178,200	811,800	1,500	0.05	0.99816
**5[b]**	**AI-assisted**	4,600	238,800	756,200	400	0.02	0.99947
	**Unaided**	4,250	179,100	815,900	750	0.02	0.99908
**1**	**AI-assisted**	920	239,760	759,240	80	0.004	0.99989
	**Unaided**	850	179,820	819,180	150	0.005	0.99982
**Lo et al. 2018[1]**		Sensitivity		Specificity		Number of participants (CT scan images)	
**AI-assisted**		0.80		0.84		324	
**Unaided**		0.65		0.90		324	
**Predicted outcome** Per 1,000,000 participants							
**Prevalence of people with malignant nodule (per 1000)**		True positive	False positive	True negative	False negative	PPV	NPV
**10**	**AI-assisted**	8,000	158,400	831,600	2,000	0.05	0.99760
	**Unaided**	6,500	99,000	891,000	3,500	0.06	0.99609
**5[b]**	**AI-assisted**	4,000	159,200	835,800	1,000	0.02	0.99880
	**Unaided**	3,250	99,500	895,500	1,750	0.03	0.99805
**1**	**AI-assisted**	800	159,840	839,160	200	0.005	0.99976
	**Unaided**	650	99,900	899,100	350	0.006	0.99961

NPV, Negative predicted value; PPV, Positive predicted value.

[b] Based on cancer prevalence observed in the NELSON trial (344 cancers detected over 10 years of follow-up minus 44 interval cancers, divided by 6583 participants for baseline scan: 300/6583 = 4.6%).[3]

References for supplementary material 9

1. Lo SB, Freedman MT, Gillis LB, et al. Journal club: computer-aided detection of lung nodules on ct with a computerized pulmonary vessel suppressed function. *AJR Am J Roentgenol* 2018;210(3):480-88. doi: 10.2214/AJR.17.18718

2. Singh R, Kalra MK, Homayounieh F, et al. Artificial intelligence-based vessel suppression for detection of sub-solid nodules in lung cancer screening computed tomography. *Quant Imaging Med Surg* 2021;11(4):1134-43. doi: 10.21037/qims-20-630

3. de Koning HJ, van der Aalst CM, de Jong PA, et al. Reduced lung-cancer mortality with volume CT screening in a randomized trial. *N Engl J Med* 2020;382(6):503-13. doi: 10.1056/NEJMoa1911793

4. Hwang EJ, Goo JM, Kim HY, et al. Implementation of the cloud-based computerized interpretation system in a nationwide lung cancer screening with low-dose CT: comparison with the conventional reading system. *Eur Radiol* 2021;31(1):475-85. doi: 10.1007/s00330-020-07151-7

5. Park S, Park H, Lee SM, et al. Application of computer-aided diagnosis for Lung-RADS categorization in CT screening for lung cancer: effect on inter-reader agreement. *Eur Radiol* 2022;32:1054–64. doi: 10.1007/s00330-021-08202-3

Supplementary material 10: References for the 9 identified non-comparative systematic reviews on stand-alone AI software performance of algorithms that are not commercially available

Discussion, pages 16/17

"Nine of these [systematic reviews] were non-comparative and focused on stand-alone AI performance of algorithms that were not commercially available, so were not informative for our review question…"

Aggarwal R, Sounderajah V, Martin G, et al. Diagnostic accuracy of deep learning in medical imaging: a systematic review and meta-analysis. NPJ Digit Med 2021;4(1):65. doi: 10.1038/s41746-021-00438-z

Forte GC, Altmayer S, Silva RF, et al. Deep learning algorithms for diagnosis of lung cancer: a systematic review and meta-analysis. Cancers (Basel) 2022;14(16):3856. doi: 10.3390/cancers14163856

Huang G, Wei X, Tang H, Bai F, Lin X, Xue D. A systematic review and meta-analysis of diagnostic performance and physicians' perceptions of artificial intelligence (AI)-assisted CT diagnostic technology for the classification of pulmonary nodules. J Thorac Dis 2021;13(8):4797-811. doi: 10.21037/jtd-21-810

Jin H, Yu C, Gong Z, Zheng R, Zhao Y, Fu Q. Machine learning techniques for pulmonary nodule computer-aided diagnosis using CT images: a systematic review. Biomed Signal Process Control 2023;79:104104. doi: https://doi.org/10.1016/j.bspc.2022.104104

Li D, Mikela Vilmun B, Frederik Carlsen J, Albrecht-Beste E, Ammitzbøl Lauridsen C, Bachmann Nielsen M, Lindskov Hansen K. The performance of deep learning algorithms on automatic pulmonary nodule detection and classification tested on different datasets that are not derived from LIDC-IDRI: a systematic review. Diagnostics (Basel) 2019;9(4). doi: 10.3390/diagnostics9040207

Liu M, Wu J, Wang N, et al. The value of artificial intelligence in the diagnosis of lung cancer: a systematic review and meta-analysis. PLoS One 2023;18(3):e0273445. doi: 10.1371/journal.pone.0273445

Pacurari AC, Bhattarai S, Muhammad A, et al. Diagnostic accuracy of machine learning AI architectures in detection and classification of lung cancer: a systematic review. Diagnostics (Basel) 2023;13(13). doi: 10.3390/diagnostics13132145

Thong LT, Chou HS, Chew HSJ, Lau Y. Diagnostic test accuracy of artificial intelligence-based imaging for lung cancer screening: a systematic review and meta-analysis. Lung Cancer 2023;176:4-13. doi: 10.1016/j.lungcan.2022.12.002

Zhang X, Liu B, Liu K, Wang L. The diagnosis performance of convolutional neural network in the detection of pulmonary nodules: a systematic review and meta-analysis. Acta Radiol 2023;64(12):2987-98. doi: 10.1177/02841851231201514
